# PrimPol toggled by ATR modulates fork slowing and repriming under oncogenic KRAS-induced replication stress

**DOI:** 10.1038/s41467-026-77585-y

**Published:** 2026-10-01

**Authors:** Taichi Igarashi, Takaaki Yasuhara, Bunsyo Shiotani

**Affiliations:** 1 Laboratory of Genome Stress Response, Radiation Biology Center, Graduate School of Biostudies, Kyoto University, Kyoto, Japan; 2 Department of Genome Stress Signaling, Institute of Medical Science, Tokyo Medical University, Tokyo, Japan

**Keywords:** Oncogenes, Stalled forks, Translesion synthesis

## Abstract

During the early phases of oncogenic clonal expansion, precancerous cells experience replication stress that threatens both genomic integrity and viability. How replication stress tolerance (RST) mechanisms are dynamically reprogrammed across the different stages of cancer progression remains elusive. Here, we identify PrimPol as a central regulator that executes two fundamentally distinct RST modes in response to oncogenic KRAS-induced heterochromatin-associated replication stress (K-HaRS). Unexpectedly, during acute K-HaRS, an unphosphorylated form of PrimPol cooperates with the fork remodeler SMARCAL1 to actively slow fork progression, stabilizing challenged forks and limiting engagement of error-prone DNA synthesis. As K-HaRS persists, PrimPol undergoes an ATR-dependent functional switch, whereby its phosphorylated form promotes repriming while fork slowing is attenuated, enabling replication to proceed across heterochromatin barriers despite persistent stress. Strikingly, loss of PrimPol enforces chronic reliance on the error-prone Rev1-Polζ-dependent synthesis and compromises cellular fitness under KRAS-driven stress. Thus, these findings establish PrimPol as a context-dependent regulator of RST that, through ATR-dependent toggling of its functional modes, coordinates fork slowing and repriming to safeguard replication fork stability while permitting an optimal compromise of genome stability that would fuel oncogenic clonal evolution.

## Abstract (web summary)

Oncogenic KRAS causes replication stress that threatens genome stability during cancer development. Here, the authors show that ATR-regulated PrimPol switches between fork slowing and repriming modes to help cells tolerate KRAS-induced replication stress while limiting error-prone DNA synthesis.

## Introduction

DNA replication stress (RS) is a central source of genomic instability (GIN)^1^, a hallmark of cancer^2,3^ and a major driver of clonal evolution^4,5^. Oncogene activation frequently perturbs replication fork progression^6^, generating single-stranded DNA (ssDNA) and other vulnerabilities that, if not properly resolved, can lead to mutations and chromosomal alterations^7^. Since acute RS is typically detrimental, triggering replication failure^8,9^, checkpoint activation, and senescence^10,11^, precancerous cells must acquire mechanisms of replication stress tolerance (RST)^12^ to bypass these anti-tumor barriers and survive during clonal expansion^9,13,14^. However, how RST operates across different stages of tumor evolution remains incompletely understood.

Among oncogenes, the Kirsten rat sarcoma viral oncogene homolog (KRAS) is the most frequently mutated in human cancers and is associated with poor prognosis^1,15,16^. Oncogenic RAS mutants, typically harboring a substitution at glycine 12, constitutively activate the RAS cascades^17^. Despite extensive studies on RAS signaling^18^, the molecular basis of oncogenic RAS-induced RS has only recently begun to emerge. We have previously demonstrated that oncogenic KRAS^G12V^ expression in untransformed epithelial cells induces facultative heterochromatin composed of H3K27me3 and HP1 in an EZH2- and transcription-dependent manner^9^, generating persistent replication obstacles that impair replication fork progression and cellular proliferation. Surviving cells adapt to KRAS-induced RS by upregulating ATR^9^, which in turn activates primase-polymerase (PrimPol), implicating PrimPol-dependent repriming^19–21^ as a key source of RST. This pathway enables forks to skip obstacles by repriming downstream, leaving ssDNA behind the replication fork^22,23^, which is closely linked to the GIN. While multiple RST pathways coexist, including repriming, fork reversal^24,25^, and translesion DNA synthesis (TLS)^26^, it remains unclear which specific RST mechanism underlies both the initial survival during oncogenic transformation and the long-term maintenance of cancer cell fitness under KRAS-driven replication stress.

Here, we uncovered a mode transition of PrimPol in response to oncogenic KRAS-induced heterochromatin-associated replication stress (K-HaRS). In normal cells experiencing acute K-HaRS, PrimPol collaborates with the fork remodeler SMARCAL1 to actively slow the replication fork progression, thereby suppressing unscheduled TLS and promoting cell survival. In contrast, in adapted or chronic K-HaRS states following oncogenic transformation, ATR actively reprograms PrimPol function to favor repriming while attenuating the SMARCAL1-mediated fork slowing mode, thereby facilitating sustained replication. These findings establish the RS-RST pairing exemplified by K-HaRS and PrimPol-dependent RST as a context-dependent determinant of GIN, providing insights into how oncogenic KRAS drives cellular survival, precancerous clonal expansion, and tumor heterogeneity.

## Results

### PrimPol is required for active fork slowing during an acute K-HaRS response

**Fig. 1:**
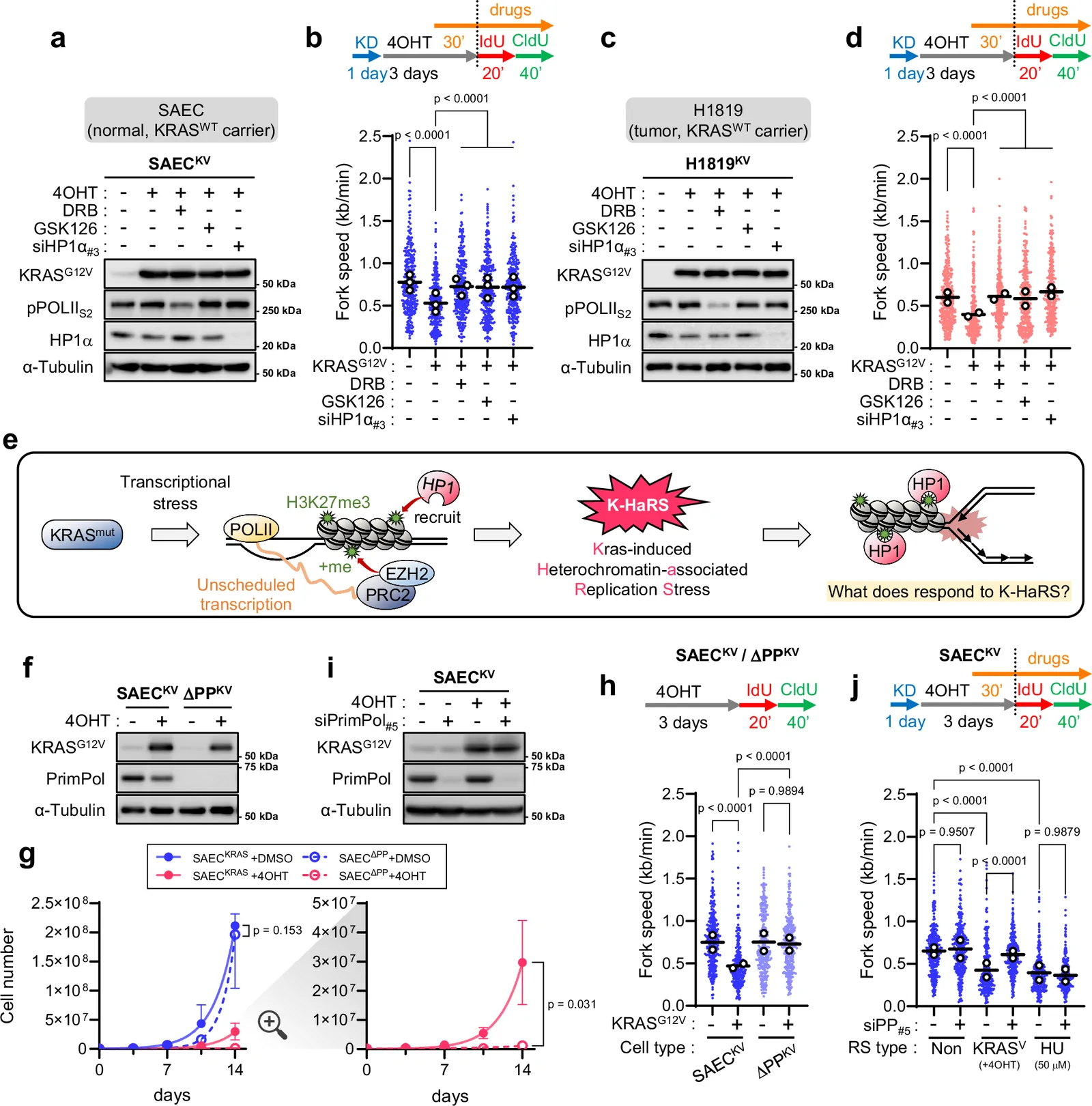
PrimPol is required for active fork slowing during an acute K-HaRS response. **a** Western blot analysis of SAEC harboring 4OHT-inducible KRAS^G12V^ (SAEC^KV^) treated with 100 μM DRB, 2.5 μM GSK126, or 1 nM siRNA targeting HP1α. **b** Scatter dot plot and mean of DNA fiber in SAEC^KV^, performed as in the top scheme. *n* = 3 biological replicates, 100 fibers per replicate. **c** Western blot analysis of H1819 harboring 4OHT-inducible KRAS^G12V^ (H1819^KV^) treated with 100 μM DRB, 2.5 μM GSK126, or 1 nM siRNA targeting HP1α. **d** Scatter dot plot and mean of DNA fiber in H1819^KV^, performed as in the top scheme. n = 2 biological replicates, 150 fibers per replicate. **e** Schematic illustration of KRAS-induced heterochromatin-associated replication stress (K-HaRS). **f** Western blot analysis of ΔPP^KV^ cells, generated by CRISPR-Cas9-mediated PrimPol knockout in SAECs, followed by integration of 4OHT-inducible KRAS^G12V^. **g** Long-term time-to-progression assay of SAEC^KV^ and ΔPP^KV^ for 14 days upon 0.1 μM 4OHT treatment. The results are represented as mean ± SEM of three independent experiments. Two-tailed ratio paired t test. **h** Scatter dot plot and mean of DNA fiber in SAEC^KV^ and ΔPP^KV^, performed as in the top scheme. n = 2 biological replicates, 150 fibers per replicate. **i** Western blot analysis of SAEC^KV^ transfected with 1 nM siRNA targeting PrimPol. **j** Scatter dot plot and mean of DNA fiber in SAEC^KV^ with KRAS^G12V^ expression or 50 μM HU treatment, performed as in the top scheme. n = 2 biological replicates, 150 fibers per replicate. **b, d, h, j** one-way ANOVA Tukey’s test. Source data are provided as a Source Data file.

To clarify how cellular contexts, such as cellular background and transformation status, affect the responses to oncogenic KRAS-induced RS, we first employed immortalized human small airway epithelial cells (SAEC)^9,27^ stably expressing retrovirally integrated 4-hydroxytamoxifen (4OHT)-inducible KRAS^G12V^ as a model for the initial step of oncogenesis (Supplementary Fig. 1a). Using this system, we performed a DNA fiber assay to determine the impact of KRAS^G12V^ expression on replication fork dynamics^28^. Consistent with previous reports^8,9,29–31^, acute KRAS^G12V^ expression restrained replication fork progression and led to a reduction in fork speed by approximately 30% in this system (Supplementary Fig. 1b), highlighting active fork slowing as one of the principal mechanisms of oncogenic KRAS-induced RST. Importantly, active fork slowing was reversed by the inhibition of three mechanistically distinct but functionally linked mechanisms (Fig. 1a, b). First, treatment with the CDK9 inhibitor 5, 6-Dichloro-1-β-D-Ribofuranosyl-1H-Benzimidazole (DRB), which efficiently inhibits RNA polymerase II (POLII) elongation^32^, fully restored the fork speed, suggesting that KRAS^G12V^-induced transcriptional stress is a key source of RS. Second, EZH2 inhibition by GSK126 (GSK), which suppresses the Polycomb repressive complex 2 (PRC2) complex-mediated maintenance of H3K27me3^33^, rescued the fork speed, supporting the role of chromatin dynamics in the cause of RS. Third, HP1α depletion similarly rescued fork speed, consistent with its role in heterochromatin formation via the coordination of H3K9me3 and H3K27me3^34^. Altogether, these results recapitulate our previous model, in which oncogenic KRAS induces heterochromatin-associated RS via transcriptional stress^9^. Notably, neither DRB (100 μM) nor GSK (2.5 μM) at the concentrations used affected the basal replication speed (Supplementary Fig. 1c). Similar phenotypes were observed in hTERT-immortalized retinal pigment epithelial (RPE) cells harboring the 4OHT-inducible KRAS^G12V^ system (Supplementary Fig. 1d, e), indicating that active fork slowing in response to heterochromatin-associated RS is a common RST in at least immortalized normal epithelial cell types.

Next, to examine whether the phenomena observed in immortalized normal epithelial cells also occur in transformed cancer cells, we introduced a 4OHT-inducible KRAS^G12V^ system into lung adenocarcinoma (LUAD) cells harboring wild-type KRAS (H1819 and H322) (Fig. 1c and Supplementary Fig. 1f). Acute KRAS^G12V^ expression markedly restrained fork progression in KRAS^WT^ LUAD cells (Fig. 1d and Supplementary Fig. 1g), mirroring the phenotypes observed in normal epithelial cells. Treatment with DRB or GSK, as well as depletion of HP1α, restored fork speed, indicating that heterochromatin-associated RS was induced even in transformed cells (Fig. 1d and Supplementary Fig. 1g). Taken together, these results demonstrate that KRAS^G12V^ consistently induces oncogenic KRAS-induced heterochromatin-associated RS (hereafter referred to as K-HaRS) in both normal and transformed epithelial cells (Fig. 1e). Thus, K-HaRS represents a stressed replication state imposed by oncogenic KRAS, and our data suggest that active fork slowing constitutes an immediate RST to preserve fork stability under this stress^12^.

PrimPol is known to support tolerance to RS by repriming around KRAS-induced heterochromatin, thereby maintaining fork speed and cellular survival^9^, as well as by DNA damage tolerance that contributes to chemotherapy resistance^35–38^. We next investigated the role of PrimPol in response to K-HaRS, particularly its potential involvement in active fork slowing^39,40^. Using the CRISPR/Cas9 system, we generated PrimPol knockout (KO) cells (ΔPP) harboring 4OHT-inducible KRAS^G12V^ (Fig. 1f) and monitored cellular proliferation. Consistent with our previous report, KRAS^G12V^ expression significantly limited cell proliferation within 14 days (Fig. 1g). However, a small subset of cells survived, potentially representing the K-HaRS-tolerant populations. Interestingly, the loss of PrimPol had no impact on the proliferation of unperturbed cells but substantially impaired growth upon KRAS^G12V^ induction, suggesting that PrimPol is required for cellular tolerance to K-HaRS (Fig. 1g). Surprisingly, ΔPP cells maintained fork speed after KRAS^G12V^ expression, whereas PrimPol-proficient cells exhibited fork slowing, indicating that PrimPol actively contributes to fork slowing (Fig. 1h). These results suggest that, beyond its canonical role in repriming, PrimPol may directly restrain fork progression to promote tolerance to acute K-HaRS.

To corroborate the finding that PrimPol mediates fork slowing, we depleted PrimPol using siRNA to mimic hypomorphic expression (Fig. 1i and Supplementary Fig. 1h), which had no detectable effects on DNA replication under unperturbed conditions (Fig. 1j). Consistently, fork speed was not reduced by KRAS^G12V^ expression in PrimPol-depleted cells (Fig. 1j and Supplementary Fig. 1i). In contrast, PrimPol was not required for fork slowing induced by mild RS induced by a low dose of hydroxyurea (HU) (Fig. 1j), which elicits RS through a mechanism driven by excessive reactive oxygen, distinct from K-HaRS^41^. Taken together, these results indicate that PrimPol specifically mediates active fork slowing in response to acute K-HaRS, revealing an unexpected role for PrimPol in replication fork regulation beyond repriming.

### The unphosphorylated form of PrimPol acts epistatically with SMARCAL1 to promote fork slowing

**Fig. 2:**
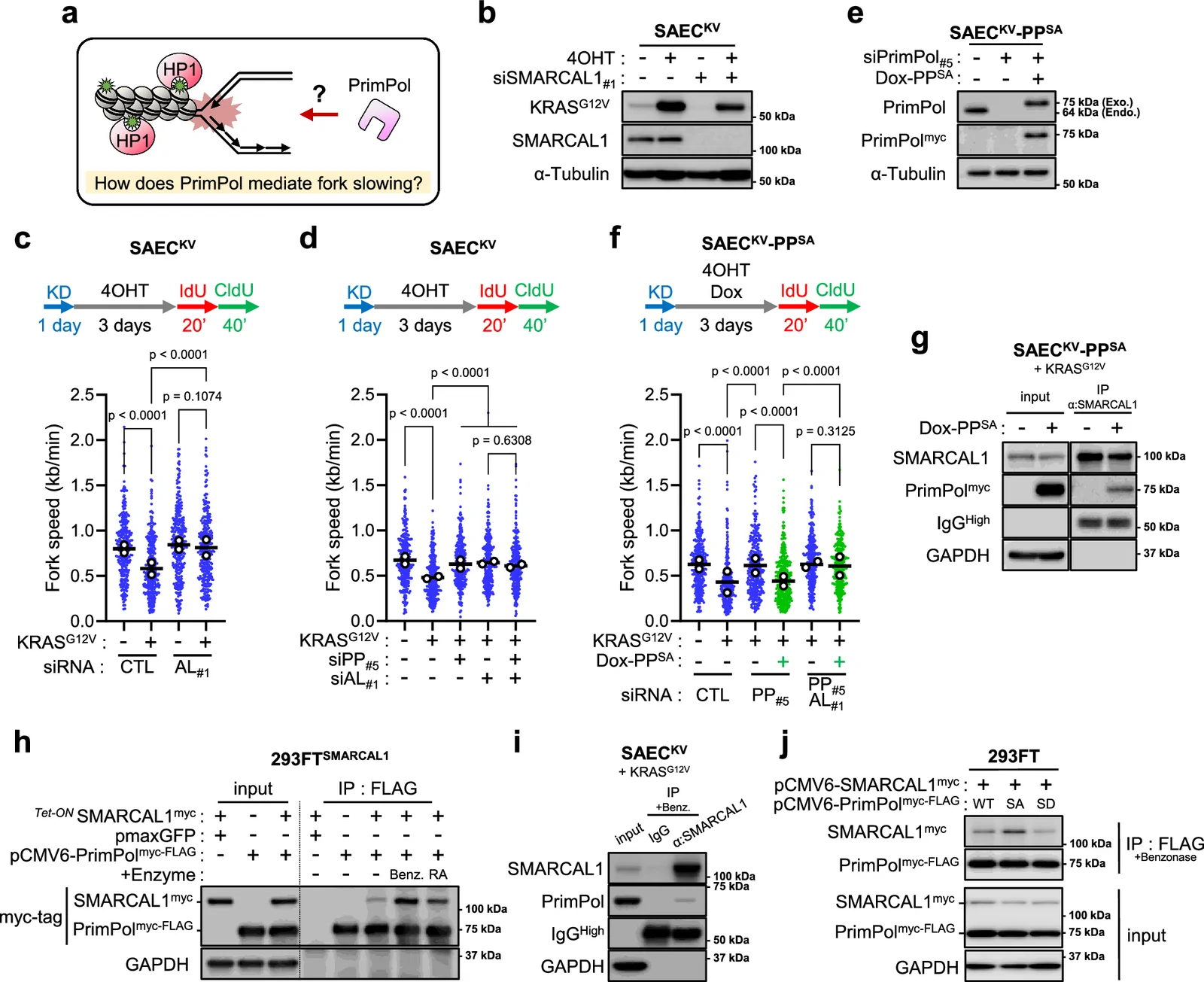
The unphosphorylated form of PrimPol acts epistatically with SMARCAL1 to promote fork slowing. **a** Question scheme: How does PrimPol slow down fork speed in response to acute K-HaRS? **b** Western blot analysis of SAEC^KV^ transfected with 1 nM siRNA targeting SMARCAL1. **c** Scatter dot plot and mean of DNA fiber in SAEC^KV^, performed as in the top scheme. n = 2 biological replicates, 150 fibers per replicate. **d** Scatter dot plot and mean of DNA fiber in SAEC^KV^ transfected with 1 nM siRNA targeting PrimPol and/or SMARCAL1, performed as in the top scheme. n = 2 biological replicates, 150 fibers per replicate. **e** Western blot analysis of SAEC^KV^ harboring Dox-inducible PrimPol^S255A^ (SAEC^KV^-PP^SA^) transfected with 1 nM siRNA targeting PrimPol. Cells were treated with 0.08 μg/mL of Dox for three days. **f** Scatter dot plot and mean of DNA fiber in SAEC^KV^-PP^SA^ transfected with1 nM siRNA targeting PrimPol and/or SMARCAL1 and treated with 0.08 μg/mL of Dox, performed as in the top scheme. n = 2 biological replicates, 150 fibers per replicate. **g** Western blot analysis of endogenous SMARCAL1 immunoprecipitation (IP) in SAEC-PP^SA^ expressing KRAS^G12V^. Cells were treated with 0.1 μM 4OHT and ±1 μg/mL Dox for three days. Representative results of three biologically independent experiments are shown. **h** Western blot analysis of exogenous PrimPol IP in 293FT harboring Dox-inducible SMARCAL1 (293FT^SMARCAL1^). Cells were transfected with pCMV6-PrimPol and treated with 1 μg/mL Dox for three days. Samples were treated with 250 U of Benzonase (Benz.) or 1 mg/mL of RNaseA (RA). Representative results of two biologically independent experiments are shown. **i** Western blot analysis of endogenous SMARCAL1 IP in SAEC^KV^ expressing KRAS^G12V^. Cells were treated with 0.1 μM 4OHT for 3 days. Representative results of two biologically independent experiments are shown. **j** Western blot analysis of exogenous PrimPol IP in 293FT. Cells were transfected with pCMV6-PrimPol (WT, S255A, S255D) and pCMV6-SMARCAL1 (WT). Exogenous proteins were detected by myc-tag antibody. Representative results of three biologically independent experiments are shown. **c**, **d**, **f** one-way ANOVA Tukey’s test. Source data are provided as a Source Data file.

To further delineate how PrimPol mediates active fork slowing in response to acute K-HaRS (Fig. 2a), we focused on its relationship with the fork remodeling translocase SMARCAL1, which is critical not only for stabilizing replication forks through fork reversal, but also for promoting active fork slowing^24,25,42,43^. As to PrimPol depletion, SMARCAL1 depletion did not affect the basal fork speed under unperturbed conditions (Fig. 2b, c). Importantly, SMARCAL1 depletion abolished fork slowing upon KRAS^G12V^ expression, indicating that SMARCAL1 is also required for fork slowing in response to acute K-HaRS (Fig. 2c). When we compared the effects of PrimPol and SMARCAL1 depletion, each reproducibly restored the fork speed to near-basal levels. Strikingly, simultaneous depletion of PrimPol and SMARCAL1 did not further relieve fork slowing, suggesting that these factors operate epistatically (Fig. 2d). This finding was reproduced in the RPE cells (Supplementary Fig. 2a, b). Moreover, PrimPol-dependent fork slowing in response to KRAS^G12V^ expression was also observed in transformed LUAD cells (H1819 and H322), supporting the idea that cellular transformation does not alter the requirement of PrimPol for fork slowing (Supplementary Fig. 2c, d). Taken together, these results suggested that PrimPol cooperates with SMARCAL1 to actively mediate fork slowing through a shared pathway, specifically in response to acute K-HaRS.

Since PrimPol promotes repriming through Ser255 phosphorylation by the ATR-Chk1 axis^9,44^, we examined whether the non-phosphorylated state of PrimPol is required for fork slowing under acute K-HaRS. We established a dox-inducible PrimPol^S255A^ expression system in SAECs (Supplementary Fig. 2e) and found that forced expression of PrimPol^S255A^ had no detectable effect on DNA replication under unperturbed or KRAS^G12V^-induced fork slowing conditions (Supplementary Fig. 2e, f). We then optimized the Dox concentration to express PrimPol^S255A^ close to the endogenous PrimPol level (Fig. 2e) and tested whether it could rescue the loss of fork slowing caused by PrimPol depletion. Surprisingly, the elevated fork speed (canceling fork slowing) caused by PrimPol depletion was markedly reduced by PrimPol^S255A^ expression (Fig. 2f). Importantly, this PrimPol^S255A^-dependent fork slowing was abrogated by SMARCAL1 depletion, indicating that the non-phosphorylatable PrimPol state functions epistatically with SMARCAL1 to mediate active fork slowing (Fig. 2f).

Furthermore, we sought to elucidate the molecular basis of PrimPol-SMARCAL1 axis in active fork slowing. A recent report has suggested a physical interaction between PrimPol and SMARCAL1 (Supplementary Fig. 2g)^39,40^. Consistently, in cells expressing both PrimPol^S255A^ and KRAS^G12V^, SMARCAL1 was found to interact with myc-tagged PrimPol^S255A^ (Fig. 2g). Since PrimPol^S255A^ restores SMARCAL1-dependent fork slowing under acute K-HaRS, this result suggested that the non-phosphorylatable PrimPol state physically associates with SMARCAL1 to support the fork-slowing mode. To further validate the PrimPol-SMARCAL1 interaction, we used a 293FT expression system and found that Flag-tagged PrimPol^WT^ co-immunoprecipitated with SMARCAL1. This interaction was enhanced rather than reduced by Benzonase and RNase A, indicating that the detected association is unlikely to be mediated by DNA or RNA (Fig. 2h). Under Benzonase-treated conditions, we also confirmed endogenous PrimPol-SMARCAL1 interaction in cells undergoing acute K-HaRS (Fig. 2i), supporting the physiological relevance of this interaction under fork slowing conditions. We then examined whether the PrimPol Ser255 phosphorylation state affects SMARCAL1 binding. In the 293FT expression system, the non-phosphorylatable PrimPol^S255A^ mutant associated with SMARCAL1 more efficiently than PrimPol^WT^ and the phosphomimetic PrimPol^S255D^ mutant (Fig. 2j). These results suggested that the non-phosphorylated PrimPol state favors SMARCAL1 association, thereby supporting active fork slowing under acute K-HaRS conditions.

### The dysregulation of PrimPol- and SMARCAL1-mediated active fork slowing triggers Rev1-Polζ-dependent DNA replication

**Fig. 3:**
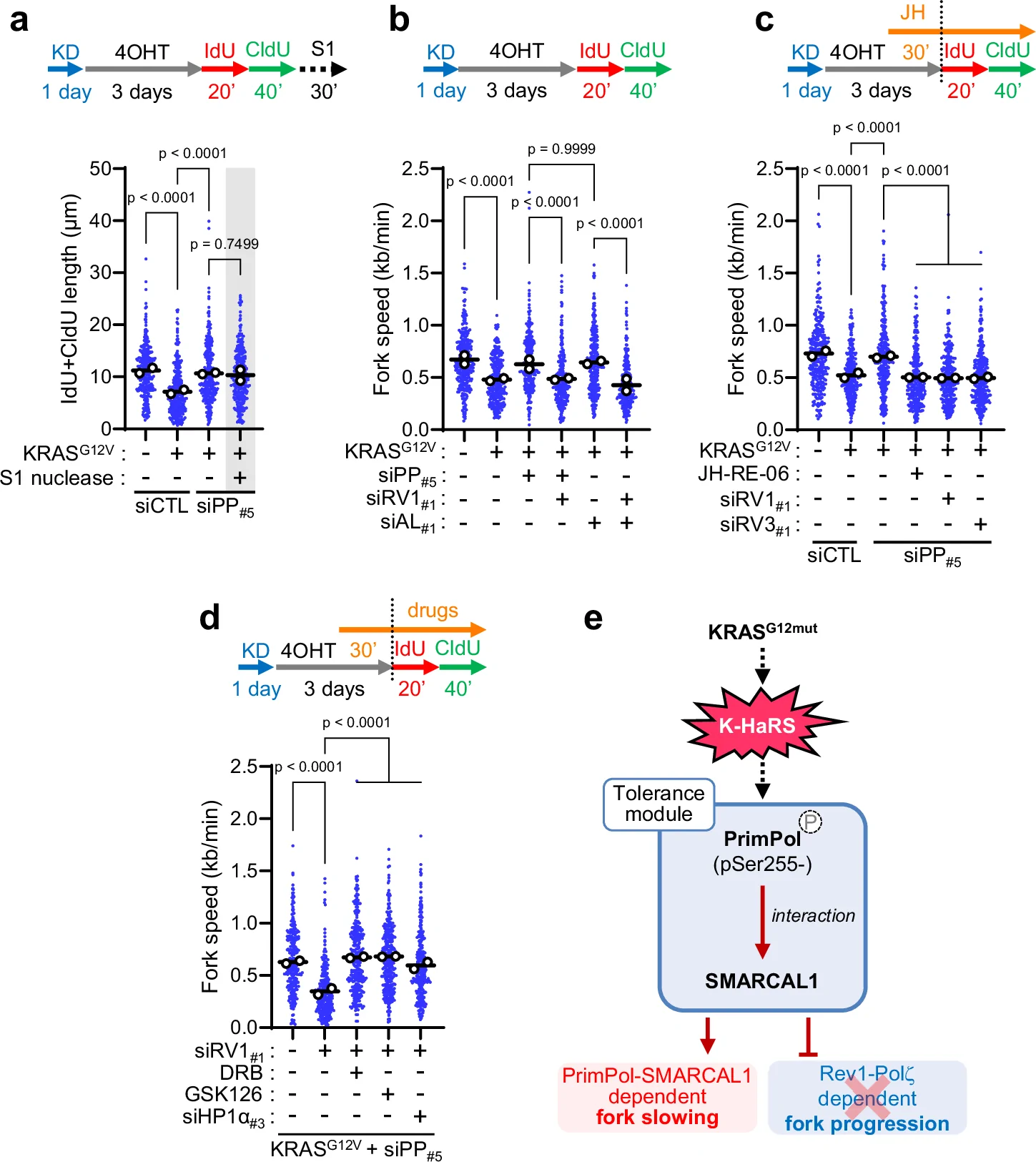
The dysregulation of PrimPol- and SMARCAL1-mediated active fork slowing triggers Rev1-Polζ-dependent DNA replication. **a** Scatter dot plot and mean of S1 DNA fiber in SAEC^KV^ transfected with 1 nM siRNA targeting PrimPol, performed as in the top scheme. Cells were permeabilized and subsequently treated with 10 U/mL S1 nuclease for 30 min. n = 2 biological replicates, 150 fibers per replicate. Also see Fig. (4b). **b** Scatter dot plot and mean of DNA fiber in SAEC^KV^ transfected with 1 nM siRNA targeting PrimPol, SMARCAL1, and/or Rev1, performed as in the top scheme. n = 2 biological replicates, 150 fibers per replicate. **c** Scatter dot plot and mean of DNA fiber in SAEC^KV^ treated with 10 μM JH-RE-06 or 1 nM siRNA targeting PrimPol, Rev1, and Rev3, performed as in the top scheme. n = 2 biological replicates, 150 fibers per replicate. **d** Scatter dot plot and mean of DNA fiber in SAEC^KV^ expressing KRAS^G12V^ transfected with 1 nM siRNA targeting Rev1 and treated with 100 μM DRB, 2.5 μM GSK126, or 1 nM siRNA targeting HP1α, performed as in the top scheme. n = 2 biological replicates, 150 fibers per replicate. **e** Schematic illustration of the acute K-HaRS tolerance module. Unphosphorylated PrimPol slows down the fork speed by interacting with SMARCAL1. Consequently, this tolerance module suppresses Rev1-Polζ-dependent replication in the vicinity of the facultative heterochromatin. **a-d** one-way ANOVA Tukey’s test. Source data are provided as a Source Data file.

Next, we investigated how DNA replication was maintained under acute K-HaRS when the PrimPol-SMARCAL1 axis was compromised. Using the S1 nuclease DNA fiber assay, which detects ssDNA gaps on nascent replication tracks, we found that replication tracks derived from unrestrained fork progression induced by PrimPol depletion upon KRAS^G12V^ expression were insensitive to S1 nuclease treatment, indicating that fork speed is maintained by continuous replication machinery in this context (Fig. 3a and Supplementary Fig. 3a). To explore whether dysregulated fork slowing activates alternative DNA polymerases, such as translesion synthesis (TLS) polymerases, that mediate continuous replication, we analyzed fork progression upon depletion of PrimPol or SMARCAL1 in KRAS^G12V^ expressing cells. As shown in Fig. 2, depletion of either PrimPol or SMARCAL1 restored fork speed in the presence of KRAS^G12V^ expression (Fig. 3b). Strikingly, simultaneous depletion of Rev1, a Y-family TLS polymerase that recruits other TLS polymerases to chromatin, restrained fork speed in the absence of PrimPol or SMARCAL1 in both SAECs and RPE cells (Fig. 3b and Supplementary Fig. 3b). Moreover, treatment with the TLS inhibitor JH-RE-06, which disrupts the interaction between Rev1 and its partner polymerases^45^, similarly abrogated the fork speed maintenance in PrimPol-depleted cells under KRAS^G12V^ expression, without affecting unperturbed replication (Fig. 3c and Supplementary Fig. 3c). Although Rev1 is activated in the absence of PrimPol upon KRAS^G12V^ expression, depletion of Rev1 itself had no impact on the fork speed with or without KRAS^G12V^ expression, suggesting that PrimPol-mediated fork slowing takes precedence over Rev1-mediated fork progression, the latter serving as an alternative pathway when fork slowing is impaired (Fig. 3c, Supplementary Fig. 3d, e).

To determine which TLS polymerases collaborate with Rev1 to promote DNA replication in the absence of PrimPol under acute K-HaRS, we individually depleted Y-family polymerases (Rev1, Polη, Polι, and Polκ) and the B-family polymerase Polζ composed of Rev3 and Rev7^46,47^. Rev3 depletion phenocopied Rev1 loss, suggesting that the Rev1-Polζ axis is responsible for fork speed maintenance in the absence of PrimPol upon K-HaRS (Fig. 3c and Supplementary Fig. 3f). Consistently, depletion of Rev7 also impaired this mechanism (Supplementary Fig. 3g, h), whereas other Y-family polymerases, except Rev1, were dispensable, probably because they primarily function in ssDNA gap filling behind the fork^48^. More importantly, the dysregulated fork speed in PrimPol/Rev1 double-deficient cells upon KRAS^G12V^ expression was rescued by inhibition of POLII or EZH2 as well as depletion of HP1α, supporting that the Rev1-Polζ axis is required to sustain DNA replication when acute K-HaRS occurs in the absence of PrimPol (Fig. 3d). This is consistent with previous findings that Polζ contributes to replication through heterochromatin^49^. Altogether, these results establish that the PrimPol-SMARCAL1 axis orchestrates active fork slowing in response to acute K-HaRS and that this function takes precedence over Rev1-Polζ-mediated fork progression (Fig. 3e).

### ATR prioritizes PrimPol-dependent repriming during adapted/chronic K-HaRS

**Fig. 4:**
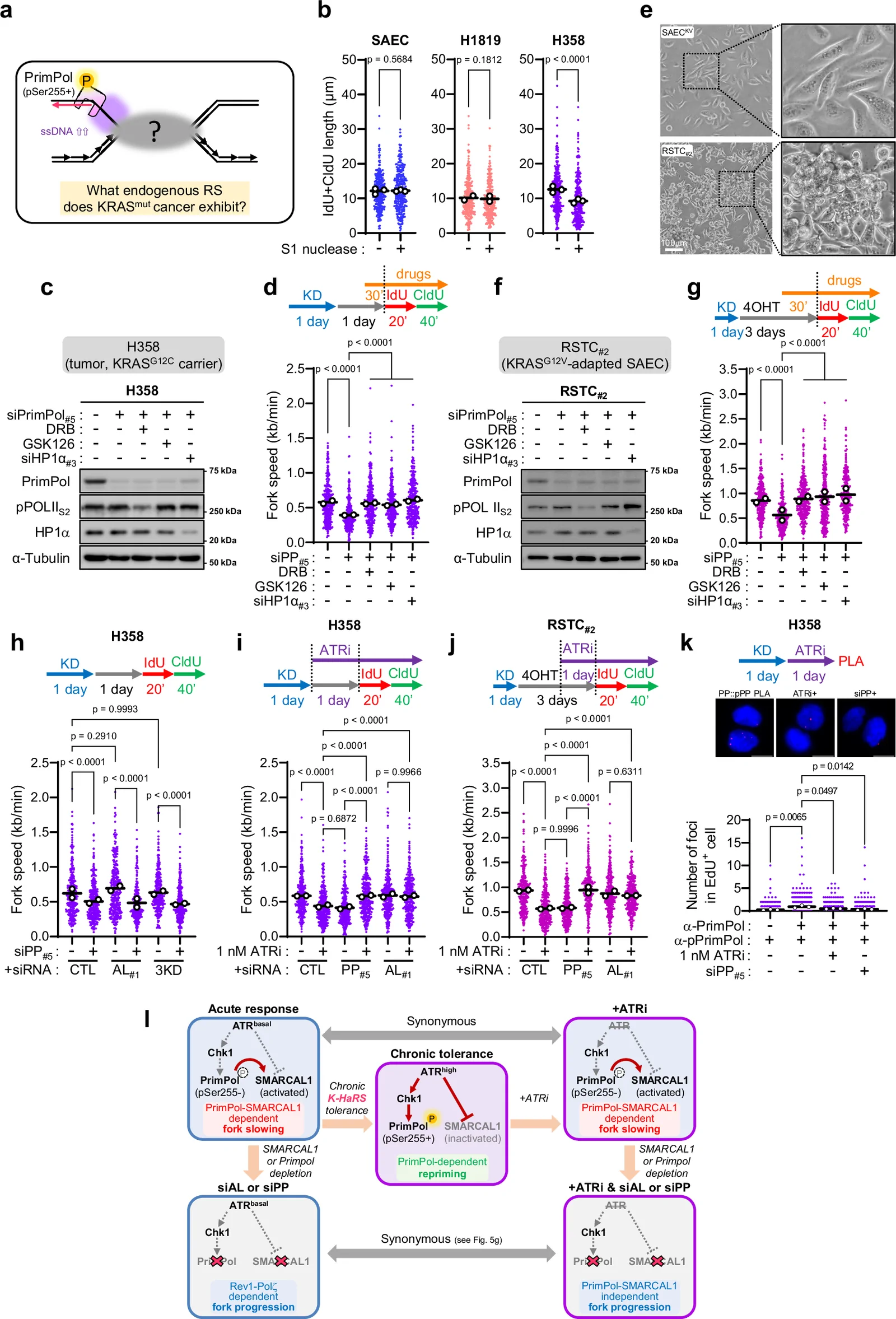
ATR prioritizes PrimPol-dependent repriming during adapted/chronic K-HaRS. **a** Question scheme: Does KRAS^mut^ cancer exhibit K-HaRS? **b** Scatter dot plot and mean of S1 DNA fiber in SAEC, H1819 and H358. Cells were permeabilized and subsequently treated with 10 U/mL of S1 nuclease for 30 min. n = 3 biological replicates, 100 fibers per replicate for SAEC and H358. *n* = 2 biological replicates, 150 fibers per replicate for H1819. **c** Western blot analysis of H358 transfected with 1 nM siRNA targeting PrimPol and treated with 100 μM DRB, 2.5 μM GSK126, or 1 nM siRNA targeting HP1α. **d** Scatter dot plot and mean of DNA fiber in H358, performed as in the top scheme. n = 2 biological replicates, 150 fibers per replicate. **e** Representative image of parental SAEC and a derived replication stress-tolerant cell (RSTC) clone, RSTC_#2_. Scale = 100 μm. **f** Western blot analysis of RSTC_#2_ transfected with 1 nM siRNA targeting PrimPol and treated with 100 μM DRB, 2.5 μM GSK126, or 1 nM siRNA targeting HP1α. **g** Scatter dot plot and mean of DNA fiber in RSTC_#2_, performed as in the top scheme. n = 2 biological replicates, 150 fibers per replicate. **h** Scatter dot plot and mean of DNA fiber in H358 transfected with 1 nM siRNA targeting PrimPol, SMARCAL1, and/or triple translocases (HLTF, ZRANB3, and SMARCAL1; 3KD), performed as in the top scheme. n = 2 biological replicates, 150 fibers per replicate. **i** Scatter dot plot and mean of DNA fiber in H358 treated with 1 nM ATRi (berzosertib) ±1 nM siRNA targeting SMARCAL1 or PrimPol, performed as in the top scheme. n = 2 biological replicates, 150 fibers per replicate. **j** Scatter dot plot and mean of DNA fiber in RSTC_#2_ treated with 1 nM ATRi (berzosertib) ± 1 nM siRNA targeting SMARCAL1 or PrimPol, performed as in the top scheme. n = 2 biological replicates, 150 fibers per replicate. **k** Representative image of proximity ligation assay (PLA) between intact PrimPol and phosphorylated Ser255 on PrimPol in H358 treated with 1 nM ATRi (berzosertib) or 1 nM siRNA targeting PrimPol, performed as in the top scheme. Scale = 10 μm. (top) Scatter dot plot and mean of n = 3 biological replicates, 200 cells per replicate. Tukey RM one-way ANOVA. (bottom) **l** Schematic illustration of ATR-dependent switching among K-HaRS tolerance modules. Acute K-HaRS engages PrimPol–SMARCAL1-dependent fork slowing, whereas adapted/chronic K-HaRS favors ATR-dependent PrimPol repriming. ATR inhibition re-engages the PrimPol–SMARCAL1 fork-slowing mode, and loss of this mode redirects replication toward Rev1-Polζ-dependent fork progression. Also see Figs. (3e) and (5f). **b**, **d**, **g**, **h-j** one-way ANOVA Tukey’s test. Source data are provided as a Source Data file.

Next, we examined whether K-HaRS persists in oncogenic KRAS-driven LUAD cancer cells (Fig. 4a). The S1 fiber assay revealed S1-sensitive replication tracks specifically in oncogenic KRAS-driven LUAD cell lines (H358 and H2009), but not in control SAECs and non-KRAS-driven LUAD cells (H1819) harboring ERBB2 amplification (Fig. 4b and Supplementary Fig. 4a). Consistent with our previous results^9^, depletion of PrimPol restrained the fork speed in oncogenic KRAS-driven cells, indicating that these cells rely on PrimPol-dependent repriming for DNA replication (Fig. 4c, d and Supplementary Fig. 4b, c). Notably, PrimPol depletion decreased fork speed by approximately 30% in H358 cells, a magnitude similar to the fork slowing induced by KRAS^G12V^ expression in SAECs (Supplementary Fig. 4d, 1b), raising the possibility that oncogenic KRAS imposes a consistent replication burden across distinct cellular contexts. Indeed, the fork slowing by PrimPol depletion was significantly alleviated by inhibition of POLII or EZH2, as well as by depletion of HP1α (Fig. 4c, d and Supplementary Fig. 4b, c), indicating that PrimPol-dependent repriming enables KRAS-driven LUAD cells to replicate through transcription- and heterochromatin-associated obstacles. To further address the adapted K-HaRS state, we analyzed replication stress tolerant cells (RSTCs), which were derived from control SAECs after prolonged KRAS^G12V^ expression and had acquired anchorage-independent growth^9^ with distinct transformed morphology compared with control SAECs (Fig. 4e). When PrimPol was depleted in RSTCs, thereby impairing repriming, replication fork progression was compromised. This defect was rescued by DRB, GSK126, or HP1α depletion, indicating that the underlying replication obstacle in the adapted state remains transcription- and heterochromatin-associated (Fig. 4f, g). Collectively, these results demonstrate that oncogenic KRAS continuously generates K-HaRS even after cells have adapted to persistent RS and undergone transformation, thereby enforcing a PrimPol-dependent repriming mode as a dominant RST pathway. This RST mechanism represents a distinctive feature of KRAS-driven cancer cells rather than a universal feature of transformed cells.

Among the multiple RST mechanisms, how oncogenic KRAS-driven cancer cells ensure the dominance of PrimPol-dependent repriming under chronic K-HaRS remains unclear. To address this, we depleted PrimPol and other factors that regulate fork stability in H358 cells and examined how the resulting phenotypes were modulated. The restrained fork speed caused by PrimPol depletion was not rescued by simultaneous depletion of SMARCAL1 (Fig. 4h). We further tested the contribution of other fork remodeling translocases, HLTF, and ZRANB3 (Supplementary Fig. 4e, f). However, triple knockdown (3KD) of SMARCAL1, HLTF, and ZRANB3 failed to restore fork speed (Fig. 4h), suggesting that fork slowing via translocases, particularly SMARCAL1, is suppressed in oncogenic KRAS-driven cancer cells. We next investigated whether ATR or Chk1 activity is required to maintain the repriming-dominant response to chronic K-HaRS. Treatment with a low dose of the ATR inhibitor (ATRi: Berzosertib, 1 nM) or Chk1 inhibitor (Chk1i: Rabusertib, 1 nM), which did not substantially alter cell cycle distribution (Supplementary Fig. 4g), restrained fork speed in H358 cells, indicating that ATR-Chk1 signaling is required to sustain PrimPol-mediated repriming (Fig. 4i and Supplementary Fig. 4h). In contrast, inhibition of ATM or DNA-PK did not phenocopy ATR inhibition under the same conditions (Supplementary Fig. 4i, j). Strikingly, SMARCAL1 depletion restored fork speed in the presence of ATRi (Fig. 4i) but not Chk1i (Supplementary Fig. 4h), suggesting that ATR, but not Chk1, suppresses SMARCAL1 activity in response to chronic K-HaRS in oncogenic KRAS-driven cancers (Supplementary Fig. 4k). Given that the non-phosphorylated state of PrimPol cooperates with SMARCAL1 to actively slow forks during the acute K-HaRS response in normal cells (Fig. 3e), we examined whether PrimPol regains this function under ATR inhibition. Indeed, the fork slowing induced by ATRi was alleviated by simultaneous PrimPol depletion (Fig. 4i). These results support a model in which ATR inhibition suppresses the PrimPol-dependent repriming mode and exposes a PrimPol- and SMARCAL1-dependent active fork slowing mode. A similar phenotype was observed in RSTCs, which represent an adapted K-HaRS state derived from SAEC after prolonged KRAS^G12V^ expression (Fig. 4j).

To further determine whether PrimPol Ser255 phosphorylation is associated with this ATR-dependent repriming mode, we newly generated a phospho-specific antibody against PrimPol Ser255 and validated its specificity using biochemical assays. In a Phos-tag assay using purified full-length PrimPol as a substrate and Chk1 as a kinase, we observed an ATP-dependent mobility shift that was abolished by Chk1 inhibition (Supplementary Fig. 4l). In addition, a peptide kinase assay using a GST-fused PrimPol peptide surrounding Ser255 detected a phosphorylation-dependent signal with the wild-type peptide but not with the S255A mutant peptide (Supplementary Fig. 4m). Since endogenous PrimPol Ser255 phosphorylation was difficult to detect reliably by conventional immunoblotting, likely reflecting its low-stoichiometry and/or transient nature, we applied this antibody to a PLA-based assay in cells utilizing intact PrimPol as a partner. PrimPol Ser255 phosphorylation-associated PLA signals were readily detected in the KRAS-driven LUAD cell lines H358 and H2009, and these signals were reduced by ATR inhibition or PrimPol depletion (Fig. 4k and Supplementary Fig. 4n). In contrast, only limited PLA signals were detected in SAECs or in non-KRAS-driven LUAD cells (H1819 and H322) (Supplementary Fig. 4o). These results indicate that PrimPol Ser255 phosphorylation-associated signals are preferentially enriched in KRAS-driven repriming-active cells and depend on ATR pathway activity.

Taken together, we propose that ATR acts as a molecular switch, toggling PrimPol between an active fork-slowing mode (in cooperation with SMARCAL1 during the acute K-HaRS response in normal cells) and a Ser255 phosphorylation-associated repriming mode (during adapted/chronic K-HaRS tolerance in oncogenic KRAS-driven cells), thereby coordinating fork stability and sustained replication throughout the oncogenic process (Fig. 4l and Supplementary Fig. 4k).

### ATR-dependent PrimPol regulation switches the K-HaRS response between repriming and active fork slowing

**Fig. 5:**
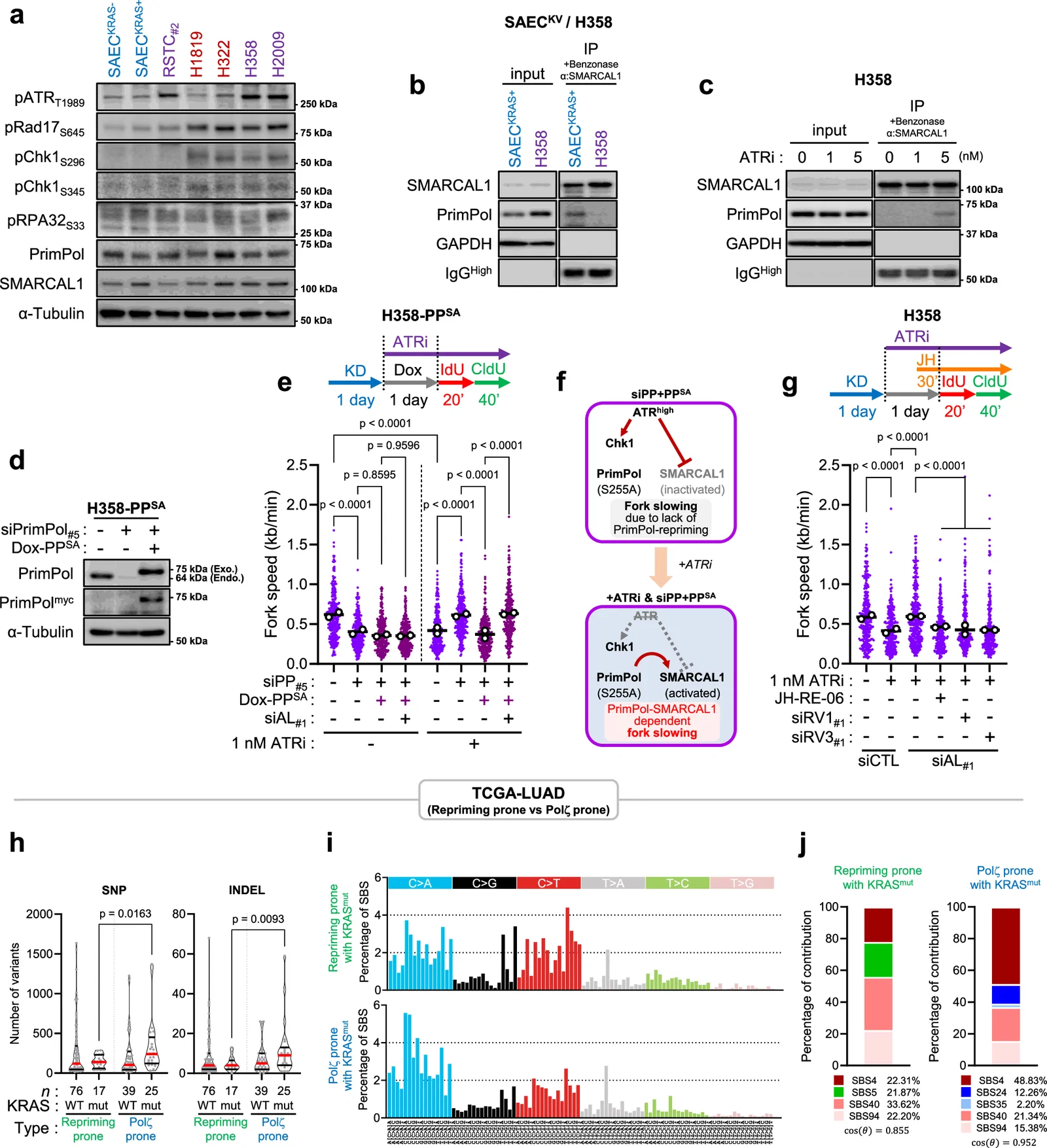
ATR-dependent PrimPol regulation switches the K-HaRS response between repriming and active fork slowing. **a** Western blot analysis of SAEC^KV^ with or without KRAS^G12V^ expression, RSTC_#2_, KRAS^WT^ LUAD cells H1819 and H322, and KRAS^mut^ LUAD cells H358 and H2009. Cells were harvested after three days incubation. **b** Western blot analysis of endogenous SMARCAL1 IP in SAEC^KV^ treated with 0.1 μM 4OHT for 3 days and H358. Representative results of two biologically independent experiments are shown. **c** Western blot analysis of endogenous SMARCAL1 IP in H358. Cells were treated with 1 or 5 nM ATRi for 24 h. Representative results of two biologically independent experiments are shown. **d** Western blot analysis of H358 harboring Dox-inducible PrimPol^S255A^ (H358-PP^SA^) transfected with 1 nM siRNA targeting PrimPol. Cells were treated with 0.04 μg/mL of Dox for one day. **e** Scatter dot plot and mean of DNA fiber in H358-PP^SA^ transfected with 1 nM siRNA targeting PrimPol and/or SMARCAL1 and treated with 0.04 μg/mL of Dox ± 1 nM ATRi, performed as in the top scheme. n = 2 biological replicates, 150 fibers per replicate. **f** Schematic illustration showing that ATR inhibition re-engages a PrimPol^S255A^-SMARCAL1-dependent fork-slowing mode in transformed cells. Also see Figs. (3e) and (4l). **g** Scatter dot plot and mean of DNA fiber in H358 treated with 1 nM ATRi, 10 μM JH-RE-06, or 1 nM siRNA targeting SMARCAL1, Rev1, and Rev3, performed as in the top scheme. n = 2 biological replicates, 150 fibers per replicate. **h** Mutation profiling of the TCGA-LUAD cohort. The number of variations in SNP or INDEL upon Repriming-prone or Polζ-prone character. See also Supplementary Fig. (5c, d). Two-sided Mann-Whitney test. **i** SBS mutational spectrum in the Repriming-prone or Polζ-prone group harboring KRAS mutations. **j** Extracted SBS signature. Respective contribution (%) and cosine similarity; cos(*θ*) between original and reconstructed profile, as in the bottom. **e**,** g** one-way ANOVA Tukey’s test. Source data are provided as a Source Data file.

To investigate how ATR-dependent signaling controls the balance between active fork slowing and repriming, we compared the levels of PrimPol, SMARCAL1, and replication stress response (RSR) markers across cell models representing acute and adapted/chronic K-HaRS states. Total PrimPol and SMARCAL1 levels were broadly comparable among these models, indicating that the functional transition between fork slowing and repriming is unlikely to be explained simply by changes in their abundance (Fig. 5a). In contrast, RSR activation markers showed model-dependent changes. Rad17 Ser645 and Chk1 Ser296/Ser345 phosphorylation were more prominent in established lung cancer cell lines than in SAECs, irrespective of KRAS mutation status, consistent with the idea that cancer cells experience elevated endogenous replication stress and broadly engage ATR-Chk1 signaling^50^. However, ATR Thr1989 phosphorylation was more prominently detected in RSTCs and the KRAS-driven H358 and H2009 cells, which exhibit PrimPol-dependent repriming activity. Thus, global ATR/RSR activation is associated with, but does not fully define, the PrimPol-dependent repriming state.

We next examined whether the PrimPol-SMARCAL1 interaction differs between the acute fork-slowing state and the chronic repriming state. Endogenous PrimPol was co-immunoprecipitated with SMARCAL1 in SAECs undergoing acute K-HaRS, whereas this interaction was barely detectable in H358 cells under basal conditions (Figs. 5b and 2i). These results suggest that the PrimPol-SMARCAL1-associated fork-slowing configuration is available in normal epithelial cells but is suppressed or disengaged in repriming-dominant KRAS-driven cancer cells. Notably, ATR inhibition restored detectable PrimPol-SMARCAL1 association in H358 cells (Fig. 5c). This finding is consistent with the functional re-engagement of SMARCAL1-dependent active fork slowing when ATR-dependent repriming is suppressed (Fig. 4i, j).

We next tested whether the ATRi-induced reappearance of PrimPol-SMARCAL1 association is accompanied by functional restoration of the active fork-slowing mode. To this end, we expressed PrimPol^S255A^ in H358 cells with or without ATR inhibition (Fig. 5d, e). Consistent with previous reports^9,44^, the reduced fork speed caused by PrimPol depletion under basal H358 condition was not rescued by PrimPol^S255A^, in line with the inefficient repriming activity of this mutant in cells (Fig. 5e; lanes 2 and 3). In contrast, the elevated fork speed observed upon combined PrimPol depletion and ATR inhibition was suppressed by PrimPol^S255A^ expression (Fig. 5e; lanes 6 and 7). Furthermore, this fork slowing was alleviated by SMARCAL1 depletion (Fig. 5e; lane 8), reinforcing the notion that ATR inhibition in oncogenic KRAS-driven cancer cells re-engages a PrimPol^S255A^- and SMARCAL1-dependent active fork-slowing mode (Figs. 4l and 5f). Thus, ATR inhibition functionally restores a fork response reminiscent of the acute K-HaRS response in normal cells (Fig. 3e). Next, we investigated how oncogenic KRAS-driven cancer cells sustain fork progression when this re-engaged PrimPol-SMARCAL1 fork-slowing axis is impaired. Since Rev1-Polζ maintains fork speed when PrimPol–SMARCAL1-dependent fork-slowing is compromised during acute K-HaRS, we depleted Rev1 or Rev3 in H358 cells treated with ATRi and depleted of SMARCAL1. Consistently, the restored fork progression caused by combined ATR inhibition and SMARCAL1 depletion was restrained by Rev1 inhibition or depletion, as well as by Rev3 depletion (Fig. 5g). Thus, Rev1-Polζ-dependent DNA synthesis maintains fork progression when ATR-dependent repriming and the PrimPol-SMARCAL1 fork-slowing axis are both compromised in oncogenic KRAS-driven cancer cells. Altogether, these findings reveal that ATR-dependent PrimPol regulation controls the switch between repriming and active fork slowing in KRAS-driven cancer cells. Under basal chronic K-HaRS conditions, ATR-dependent repriming predominates, whereas PrimPol-SMARCAL1-associated active fork slowing is re-engaged when ATR activity is inhibited. If this fork slowing mode is also impaired, cells shift toward Rev1-Polζ-dependent fork progression (Figs. 4l, 5f and Supplementary Fig. 4k).

Finally, to assess whether chronic reliance on PrimPol-dependent repriming or Polζ-dependent replication leaves genomic imprints in cancer cells, we analyzed LUAD samples from The Cancer Genome Atlas (TCGA). Consistent with a previous discussion^3^, *ATR, PrimPol*, and *SMARCAL1* showed rare genetic alterations and virtually no nonsense mutations and showed no significant co-occurrence or mutual exclusivity with *KRAS* alterations (Supplementary Fig. 5a). Therefore, we stratified the tumors according to the expression levels of each gene into high- and low-expression groups (Supplementary Fig. 5b). Based on the model that ATR-PrimPol activity enforces repriming, whereas its dysfunction together with SMARCAL1 downregulation promotes Polζ-dependent bypass (Fig. 4l), we defined the “repriming-prone” group (ATR^high^, PrimPol^high^, SMARCAL1^low^) and “Polζ-prone” group (ATR^low^, PrimPol^low^, SMARCAL1^low^) (Supplementary Fig. 5c). While the KRAS^mut^ exhibited an increased burden of genomic alterations compared to KRAS^WT^ samples, the Polζ-prone group with KRAS^mut^ carried significantly more variants than the repriming-prone group with KRAS^mut^, consistent with the error-prone nature of Polζ-mediated synthesis (Fig. 5h). Importantly, this difference was retained after excluding *TP53*-mutant tumors, indicating that the increased variant burden in the KRAS^mut^ Polζ-prone group is not simply explained by *TP53* mutation status (Supplementary Fig. 5d). Mutational profiling revealed a higher proportion of C>A/G>T substitutions and a reduced proportion of C>T/G>A substitutions, specifically in the Polζ-prone group with oncogenic KRAS mutations (Fig. 5i). These features aligned with COSMIC SBS24, an aflatoxin exposure signature (Fig. 5j), in line with reports that Polζ tolerates aflatoxin in vivo^51^ and induces G>T substitutions in vitro^52^. By contrast, the repriming-prone group was enriched for SBS5, a ubiquitous signature potentially linked to aging, smoking^53^, and early replication timing^54^. Interestingly, SBS4 (smoking-related)^53^ decreased by ~50%, despite comparable smoking exposure (pack-years) between groups (Supplementary Fig. 5e). These results strongly support our model that the downregulation of the ATR-PrimPol axis forces chronic reliance on Polζ, thereby driving excessive mutations. Collectively, our findings reveal that PrimPol, through its mode transition orchestrated by ATR, stabilizes forks during acute and chronic K-HaRS, while suppressing Rev1-Polζ-mediated error-prone DNA synthesis. Importantly, this mechanism could explain our initial observation (Fig. 1g) that loss of PrimPol compromises oncogenic KRAS-driven proliferation, possibly because chronic reliance on error-prone Polζ would otherwise excessively fuel mutagenesis and impair cell fitness.

## Discussion

**Fig. 6:**
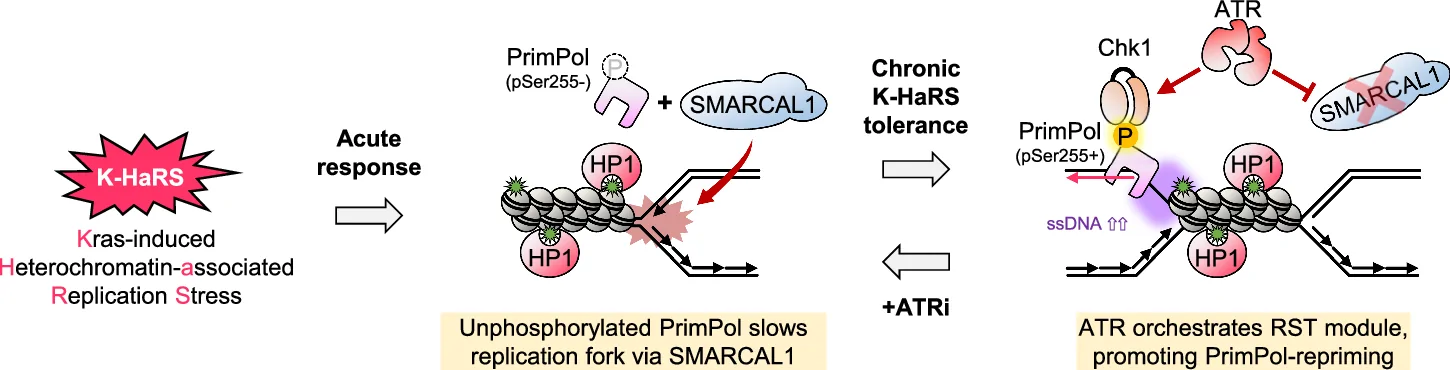
The dual function of PrimPol toggled by ATR during acute and chronic K-HaRS. During the acute response to K-HaRS, the unphosphorylated form of PrimPol associates with SMARCAL1 and slows replication fork progression to ensure fork stability. In a subset of cells that acquire replication stress tolerance (RST) mechanisms through ATR-PrimPol upregulation, ATR limits PrimPol-SMARCAL1 association and prioritizes PrimPol-mediated repriming. Together, these dual modes of PrimPol support the survival of precancerous cells and the progression of cancer cells under persistent K-HaRS, in part by consistently limiting Rev1-Polζ-dependent replication.

The causes and consequences of oncogene-induced replication stress, as well as the cellular response that mitigates it, remain unclear. Here, we demonstrate that PrimPol, through ATR-dependent mode transition, plays a pivotal role in maintaining replication fork stability during both acute and chronic responses to oncogenic KRAS-induced heterochromatin-associated replication stress (K-HaRS) (Fig. 6).

Oncogenic KRAS constitutively promotes the PRC2-dependent formation of H3K27me3-enriched heterochromatin in a transcription-dependent manner^9^, leading to persistent K-HaRS even after transformation. Importantly, oncogenic KRAS-induced heterochromatin may share the properties of typical H3K27me3-marked regions, which exhibit incomplete silencing and leaky transcription compared to other tightly repressed heterochromatin loci^55^. This low-level, abortive transcription, potentially induced by oncogenic KRAS via FOSL1-based AP-1 recruitment^56^, may in turn reinforce PRC2-mediated H3K27me3 deposition through a positive feedback loop^57,58^, thereby sustaining persistent K-HaRS. Supporting this, EZH2 inhibition improves prognosis in KRAS^G12D^-driven non-small-cell lung cancers (NSCLC)^59^, and an inhibitory phosphorylation of EZH2 (Thr311) suppresses KRAS^G12V^-driven ovarian cancer growth in mouse models^60^. Together, these observations suggest that K-HaRS functions as a driver of cancer progression and may be associated with poor clinical outcomes^12^. A deeper understanding of how oncogenic KRAS establishes K-HaRS and which genomic loci are targeted during its acute versus chronic phases is essential to clarify its role in cancer initiation and progression.

While K-HaRS intrinsically imposes a severe replication burden that limits cellular survival, the tolerance mechanisms involved in coping with K-HaRS differ depending on the stage of the oncogenic process. Our data showed that during acute K-HaRS, PrimPol collaborates with SMARCAL1 to actively slow fork progression and promote cell survival, possibly by preventing catastrophic fork collapse. This active fork slowing represents a unique form of RST under K-HaRS, where replication fork stability is maintained at the expense of the replication speed. Recent studies have proposed that translocase-mediated fork slowing is a primary strategy for preserving fork integrity^23,25^, whereas alternative pathways, such as PrimPol-dependent repriming and/or TLS, are engaged upon fork slowing failure or during post-replicative processing^36,41,61–65^. Collectively, these findings highlight that the mechanisms orchestrating the choice between fork slowing, repriming, or TLS have become a central topic in the field^22^. We found that PrimPol and SMARCAL1 act together on the same axis to enforce fork slowing during acute K-HaRS, suppressing Rev1-Polζ-mediated synthesis, and providing an example of such pathway choice mechanisms under acute RS.

In contrast, under adapted/chronic K-HaRS conditions, as observed in SAEC-derived RSTCs and KRAS-driven LUAD cells, PrimPol primarily functions through its repriming activity to sustain fork speed, indicating that both its expression and catalytic activity confer a survival advantage under oncogenic KRAS expression. Notably, SAEC-derived RSTCs retained transcription- and heterochromatin-associated fork barriers, supporting the idea that adapted/transformed cells maintain a KRAS-dependent replication burden rather than a generic transformed-cell replication stress state. Although established cancer cells showed broad RSR activation irrespective of KRAS status, PrimPol Ser255 phosphorylation-associated signals and functional repriming were preferentially linked to KRAS-driven adapted/chronic K-HaRS states. Thus, global RSR activation alone does not define the PrimPol-dependent repriming mode. Across the acute-to-adapted/chronic transition, the balance between PrimPol-dependent repriming and active fork slowing appears to fluctuate in an ATR-dependent manner, with ATR activity shifting this balance toward repriming. Consistent with this idea, the ATR/Chk1 module is required for oncogenic RAS-driven transformation and/or tumorigenesis^9,66–68^, suggesting that the repriming mode is preferentially utilized over active fork slowing during chronic K-HaRS.

Mechanistically, our data support a model in which ATR-dependent PrimPol regulation, including Ser255 phosphorylation, promotes repriming while limiting the PrimPol-SMARCAL1-associated fork-slowing mode. Given that ATR-mediated phosphorylation of SMARCAL1 at Ser652 has been reported to negatively regulate its activity^69^, ATR may also attenuate active fork slowing through SMARCAL1 regulation. However, the precise contribution of SMARCAL1 phosphorylation to the balance between fork slowing and repriming during K-HaRS tolerance remains to be determined. This ATR-dependent mode switching enables cells to tolerate chronic K-HaRS while adjusting the level of genomic instability. These findings suggest that PrimPol does not merely promote replication under stress but modulates the level of genomic instability to support both the survival of precancerous cells and their clonal expansion. It will be of great interest to determine when and how ATR is activated to orchestrate the functional switch of PrimPol and how such regulation shapes the selection of ATR-PrimPol axis-upregulated clones during oncogenic clonal expansion.

Our findings support a dynamic model in which PrimPol orchestrates distinct RST strategies during the acute and chronic phases of the K-HaRS. In naïve epithelial cells, acute K-HaRS is initially managed through PrimPol-SMARCAL1-mediated fork slowing, which might repeatedly reverse and temporarily stabilize impeded forks, allowing controlled restart events. This dynamic modulation likely prevents unchecked fork progression into challenging genomic regions, thereby promoting cell survival. As ATR signaling is established during this initial response, PrimPol is activated while SMARCAL1 is suppressed, shifting the RST from active fork slowing to repriming mode. This transition not only preserves cell viability under persistent stress, but also promotes clonal diversification, fueling oncogenic clonal expansion. Even in the chronic phase, the ability of the ATR-PrimPol axis to flexibly rewire the RST maintains heterogeneity, underpinning malignant progression and therapy resistance. This is consistent with our previous work demonstrating that high ATR-PrimPol expression correlates with poor prognosis in KRAS-mutant cancers^9^. Importantly, ATR-PrimPol activity also limits Rev1-Polζ-prone replication, preventing excessive mutational load and catastrophic genomic collapse. Consequently, oncogenic KRAS-expressing cells with intact PrimPol gain a proliferative advantage over PrimPol-deficient counterparts, despite reduced fitness compared to unstressed normal cells. Thus, these findings suggest that ATR and PrimPol fine-tune replication stress responses to create a state “just right”—*a Goldilocks zone of genome instability*—that allows precancerous cells to survive oncogenic stress, diversify, and evolve.

The distinctive responses to acute versus chronic K-HaRS underscore the importance of “RS-RST pairing diversity.” Because the sources of oncogene-induced RS are not restricted to a single mechanism^6,12^, and shift flexibly depending on the cellular context^30^, the selection of tolerance mechanisms is likely dictated by the nature of RS. For example, oncogenic RAS expression typically induces R-loop-associated transcription-replication conflicts (TRCs) in non-epithelial cells^8^, where topoisomerases provide tolerance^70^, whereas oncogenic KRAS induces heterochromatin-associated RS in epithelial cells, counteracted by ATR-PrimPol axis upregulation^9^. More generally, emerging evidence suggests that RST mechanisms are tailored to the specific nature and source of replication stress, emphasizing the critical role of context-appropriate RS-RST pairing in maintaining genome integrity^36,41,61–65^. This diversity of RS and its paired tolerance mechanisms not only allows cells to adapt to exogenous or endogenous stresses, but also fuels cellular heterogeneity, adding a new layer of complexity to the molecular basis of oncogenesis. Consequently, cellular context and epigenetic background should be considered essential determinants of RS-RST pairing. Taken together, our findings highlight that RS-RST pairing is a dynamic determinant of cancer cell fate. Disrupting one tolerance pathway can redirect replication into another, however, each RS-RST pair carries distinct trade-offs that create selective vulnerabilities. Defining context-specific targeting of individual RS-RST pairings thus represents a critical future direction with the potential not only to propose another therapeutic strategy against KRAS-driven cancers, but also to open avenues toward preventive interventions targeting genomic instability.

## Methods

### Cell culture and supplement

All the cells were cultured at 37 °C in a 5% CO_2_ incubator. 293FT cells (gifted from Dr. Takata) were cultured in Dulbecco’s modified Eagle medium (DMEM; Nacalai Tesque, Cat# 08459-64). Immortalized RPE-1 cells (a kind gift from Dr. Hochegger) were cultured in DMEM-Ham’s F-12 medium (Nacalai Tesque, Cat# 11581-15). H1819, H322, H358, and H2009 cells (purchased from ATCC) were cultured in RPMI 1640 (Nacalai Tesque, Cat# 30264-56). These media were supplemented with 10% fetal bovine serum (FBS; Sigma-Aldrich, Cat#F7524-500ML) and 10 mM HEPES (Nacalai Tesque, Cat#17557-94). SAECs (a kind gift from Dr. Kiyono) were immortalized by the expression of hTERT, CDK4^R24C^ and Cyclin D1^27^. SAECs were cultured in BronchiaLife™ Epithelial Basal Medium (Lifeline Cell Technology, Cat# LM-0007) supplemented with the components of LifeFactors® Kit (Lifeline Cell Technology, Cat# LS-1047). Cells were periodically treated with Plasmocin (InvivoGen, Cat#ant-mpt).

### Transient expression vector

For transient PrimPol expression, pCMV6-PrimPol-myc-FLAG (OriGene, Cat# rc209004) was used. For transient SMARCAL1 expression, pUC19-SMARCAL1^I784F^ (Sino Biological, Cat#HG18854-U) was purchased. After introducing the F784I substitution by site-directed mutagenesis with Pfu Turbo DNA Polymerase, SMARCAL1^WT^ cDNA was amplified and an N´-terminal myc-tag was added. myc-SMARCAL1 was inserted into the BamHI and NotI sites of pCMV6 vector.

For His-tagged PrimPol purification, PrimPol cDNA was amplified by PCR and inserted into the NheI and NotI sites downstream of His-tag in the pETUA vector (BioDynamics Laboratory, Cat#DV200). For purification of GST-conjugated PrimPol 250-260 aa fragments, DNA fragments encoding residues 250-260 of PrimPol^WT^ or PrimPol^S255A^ were synthesized and inserted into the NotI and BamHI sites of GST-2TK (a gift from Dr. Lee Zou). All construct selection and amplification were performed using XL10-Gold.

### 4OHT-inducible KRAS^G12V^ and Dox-inducible PrimPol viral construct

The pQCXIHyg-mERT2-KRAS^G12V^ [pA-] vector (a kind gift from Dr. Kiyono) was utilized for 4OHT-inducible KRAS^G12V^ expression in SAECs, RPE-1, H1819, or H322 cells as previously described^9^. To establish doxycycline-inducible PrimPol H358 cells, PrimPol-myc-FLAG cDNA was amplified by PCR using Pfu Turbo DNA Polymerase, and inserted into the BamHI and NotI sites of the pENTR GateWay vector using the In-Fusion HD cloning protocol (TaKaRa, Cat# 638948). pENTR-PrimPol-myc-FLAG was recombined into CSIV-TRE-RfA-UbC-Puro (a kind gift from Dr. Nakanishi) using the GateWay system (Invitrogen, Cat# 11791-020). Site-directed mutagenesis using Pfu Turbo DNA Polymerase and specific primers was performed to generate the PrimPol mutant (S255A, S255D). To establish doxycycline-inducible PrimPol SAECs and doxycycline-inducible SMARCAL1 293FT, PCR-amplified PrimPol-myc-FLAG and myc-SMARCAL1 cDNA were inserted into the BsmBI and XhoI sites of the pInducer20 (a kind gift from Dr. Zou) using the In-Fusion HD cloning protocol. All construct selection and amplification were performed using XL10-Gold.

### Viral production and infection

293FT cells were co-transfected with 0.5 µg/mL of retroviral pCL-GagPol, pEF6/env (10A1), and pQCXIHyg-mERT2-KRAS^G12V^ [pA-] vector, or 0.5 µg/mL of lentiviral pCAG-HIVgp, pCMV-VSV-G-RSV-Rev and CSIV-TRE-PrimPol-myc-UbC-Puro/pInd20-TRE-PrimPol-myc-UbC-IRES-Neo/pInd20-TRE-myc-SMARCAL1-UbC-IRES-Neo using 2.5 μg/mL of polyethyleneimine (PEI) “MAX” (Polyscience, Cat# 24765). The viral supernatants were collected, filtered (0.45 µm PVDF), and concentrated using a Retro-X Concentrator (TaKaRa, Cat# 631456) or Lenti-X Concentrator (TaKaRa, Cat# 631232). The concentrated virus was resuspended in a medium containing 8 μg/mL polybrene (Sigma-Aldrich, Cat# H9268) for infection of SAECs, RPE-1, H1819, H322, H358 and 293FT cells. 50 μg/mL Hygromycin B (Wako, Cat# 080-07683) or 200 μg/mL G418 (InvivoGen, Cat# ant-gn-1) was used for selection.

### CRISPR/Cas9 knockout of PrimPol in SAECs

The exon 7 region of PrimPol (5´-CAGCCTGCTCTTGACTTGCT-3´, PAM: TGG) was targeted for PrimPol knockout. The annealed gRNA oligo was inserted into the BbsI site of pSpCas9(BB)−2A-GFP (Addgene, Cat# 48138) using a Quick Ligation™ Kit (NEB, Cat# M2200L). pSpCas9(ΔPP)−2A-GFP (2 μg) was transfected into SAECs using Nucleofector™ (Lonza, Cat#VPI-1005) using the program *T-013*. After 4 days of incubation, GFP-positive cells were sorted using a BD FACSMelody™. Single clones were picked up and expanded, and PrimPol depletion was then confirmed by western blotting as described below.

### Exogenous gene expression and drug treatments

4OHT (Abcam, Cat# ab141943, 0.1 mM stock) was dissolved in dimethyl sulfoxide (DMSO). Doxycycline (Sigma-Aldrich, Cat# D9891-1G, 1 mg/mL stock) was dissolved in dH_2_O. To induce KRAS^G12V^ or PrimPol expression, cells were treated with 0.1 μM 4OHT or 0.04–1 μg/mL doxycycline for the indicated time periods, respectively_._

For transient expression of myc-FLAG tagged PrimPol and myc-tagged SMARCAL1, 293FT cells were co-transfected with pmax-GFP (Lonza), pCMV6-PrimPol-myc-FLAG and/or pCMV6-myc-SMARCAL1 using 2.5 μg/mL of PEI “MAX”. After 18–24 h of incubation with DNA, the cell culture medium was replaced with fresh medium.

The ATR inhibitor berzosertib (Selleck, Cat# S7102, 1 mM stock), Chk1 inhibitor rabusertib (Selleck, Cat# S2626, 1 mM stock), 5,6-Dichloro-1-β-D-ribofuranosyl-1H-benzimidazole (DRB; Sigma–Aldrich, Cat# D1916-10MG, 100 mM stock), GSK126 (Selleck, Cat# S7061, 5 mM stock), and JH-RE-06 (Selleck, Cat# S8850, 10 mM stock) were dissolved in DMSO and used at the indicated final concentrations.

### Gene silencing with RNAi

Cells were transfected with 1 nM siRNAs using the reverse transfection method with Lipofectamine RNAiMAX (Invitrogen, Cat# 13778-150) according to the manufacturer’s instructions. After 18–24 h of incubation with siRNAs, the cell culture medium was replaced with fresh medium. The following siRNAs were purchased from Thermo Fisher Scientific (Ambion):　Silencer Select Negative Control siRNA (Ambion, Cat# 4390843), siPRIMPOL#2 (ID# s47418), siPRIMPOL#3 (ID# s47417), siPRIMPOL#5 (custom, sense: GUCUGUGAGAUUUGAUAAAtt, antisense: UUAUCAAAUCUCACAGACaU), siHP1α#3 (custom, sense: GUUGCCCAUCUGUUAAAAAtt, antisense: UUUUUAACAGAUGGGCAACaU; siHP1α#3), siSMARCAL1#1 (ID# s531775), siRev1#1 (ID# s28165), siRev1#2 (ID# s28166), siRev3#1 (ID# s11938), siRev3#2 (ID# s11939), siRev7#4 (custom, sense: GCCCAAACUGUCAGACUUUtt, antisense: AAGUCUGACAGUUUGGGCaU), siPolη#1 (ID# s10790), siPolι#2 (ID# s22123), siPolκ#2 (ID# s28115), siHLTF#2 (ID# s13138), siZRANB3#1 (ID# s224929).

### Western blot analysis

Cells were lysed in the Sampling buffer (120 mM Tris-HCl [pH 6.8], 12% glycerol, 4% SDS, 200 mM DTT, 0.004% bromophenol blue) and denatured at 95 °C for 5 min^9^. The protein concentration in the cell lysate was estimated by the Bradford assay (XL-Bradford, APRO Science, Cat# KY-1031). Equal amounts of protein were resolved by SDS-PAGE (10–15% gel) at 120 V for 90 min and then transferred to polyvinylidene difluoride (PVDF) membranes (Immobilon-P, Millipore, Cat# IPVH00010) at 150 mA for ~16 h at 4 °C. Membranes were blocked with 5% skim milk dissolved in TBST (25 mM Tris-HCl [pH 7.8], 140 mM NaCl, 0.1% Tween 20) for 20 min at room temperature (RT) and probed with the indicated primary antibodies diluted in 5% skim milk (supplemented with 0.02% NaN_3_): anti-pan-RAS (1:1000, Santa Cruz, Cat# sc-166691), anti-phospho POLII Ser2 (1:2000, Novus, Cat#NB100-1805), anti-HP1α (1:1000, Millipore, Cat# 05-689), anti-αTubulin (1:5000, MBL, Cat# PM054), anti-PrimPol (1:3000, Proteintech, Cat# 29824-1-AP), anti-PrimPol (1:1000, Millipore, Cat#MABE2019), anti-SMARCAL1 (1:1000, CST, Cat# 44717), anti-SMARCAL1 (1:200, Santa Cruz, Cat#376377), anti-myc tag (1:500, MBL, Cat# 192-3), anti-GAPDH (1:1000, Wako, Cat#016-25523), anti-GST tag (1:1000, MBL, Cat#PM013), anti-Rev1 (1:100, Santa Cruz, Cat# sc-393022), anti-Rev7 (1:1000, abcam, Cat# ab180579), anti-Polη (1:1000, CST, Cat# 13848), anti-Polι (1:1000, Proteintech, Cat# 13635-1-AP), anti-Polκ (1:1000, Proteintech, Cat# 14455-1-AP), anti-HLTF (1:1000, Proteintech, Cat# 14286-1-AP), anti-phospho ATR Thr1989 (1:1000, CST, Cat#30632), anti-phospho ATM Ser1981 (1:1000, CST, Cat#5883), anti-phospho DNA-PKcs Ser2056 (1:1000, abcam, Cat#124918), anti-phospho Rad17 Ser645 (1:1000, Bethyl, Cat#A300-153A), anti-phospho Chk1 Ser345 (1:1000, CST, Cat#2348), anti-phospho Chk1 Ser296 (1:1000, CST, Cat# 90178), anti-phospho RPA32 Ser33 (1:1000, Bethyl, Cat#A300-246A), anti-phospho PrimPol Ser255 (1:1000, custom antibody raised against LERLG[pS]AEQSSPDLS, Sigma-Aldrich).

After washing with TBST, the membranes were incubated with horseradish peroxidase (HRP)-linked species-specific F(ab´)_2_ fragment (1:5000, Cytiva, Cat# NA9310 or Cat# NA9340) diluted in 5% skim milk for 1 h at RT and then washed with TBST. The blots were developed using Western Lightning Plus-ECL (Revvity, Cat# NEL105001EA) according to the manufacturer’s instructions and imaged using an LAS 4000 luminescent image analyzer (Fujifilm). Uncropped and unprocessed blot and gel images are provided in the Source Data file.

### DNA fiber assay and S1 fiber assay

Standard DNA fiber assay and S1 fiber assay were performed as previously described^9^. In brief, after the indicated specific treatment, cells were sequentially labeled with 100 μM IdU (5-iodo-2´-deoxyuridine, Sigma–Aldrich, Cat# l7125-5G) for 20 min, washed with PBS, labeled with 100 μM CldU (5-chloro-2´-deoxyuridine, Sigma–Aldrich, Cat# C6891-100MG) for 40 min, and washed with PBS. The labeled cells were trypsinized, pelleted at 800 ×  *g* for 5 min at 4 °C, and resuspended in 200 μL PBS. For S1 nuclease treatment, labeled cells were permeabilized with CSK100 (10 mM MOPS [pH 7.0], 100 mM NaCl, 3 mM MgCl_2_, 300 mM sucrose, 0.5% Triton X-100) for 10 min at RT, gently washed twice with S1 buffer (30 mM sodium acetate [pH 4.6], 50 mM NaCl, 10 mM zinc acetate, and 5% glycerol), treated with 10 U/mL of S1 nuclease (Invitrogen, Cat# 18001-016) in S1 buffer for 30 min at 37 °C, and collected in 500 μL of PBS containing 1% BSA (Nacalai Tesque, Cat# 01860-07) with a cell scraper. Cell nuclei were pelleted at 4900 × *g* for 5 min at 4 °C and 300 μL of the supernatant was removed, leaving 200 µL. After careful resuspension by pipetting, 1300 μL of fixative solution (3:1 of MeOH: acetic acid) was added dropwise to 200 μL of the cell suspension. The cells and nuclei were centrifuged at 800 × *g* for 5 min at 4 °C and resuspended in fixative solution to a final concentration of ~1000 cells/μL, and 25 μL of fixed cells were spotted onto slides (Matsunami, Cat# S2112). After drying for 3 min, the slides were immersed in lysis solution (200 mM Tris-HCl [pH 7.5], 50 mM EDTA, and 0.5% SDS) for ~20 min at 37 °C. DNA was spread by tilting the slides in a high-humidity chamber for at least 30 min. The slides were immersed in a fixative solution for 20 min and dried overnight at 4 °C.

After rehydration with PBS wash, DNA was denatured with 2.5 M HCl for 1 h. After washing with HCl, the slides were blocked with 5% BSA and 0.1% Tween 20 in PBS for 1 h, followed by incubation with primary antibodies. Anti-IdU (1:25, BD Biosciences, Cat#347580) and anti-CldU (1:100, Abcam, Cat#ab6326) antibodies were diluted in the blocking solution. After washing with PBST (PBS containing 0.1% Tween 20), the primary antibodies were probed with Alexa Fluor 488-conjugated anti-rat IgG (1:100, Invitrogen, Cat# A11006) and Alexa Fluor 555-conjugated anti-mouse IgG (1:100, Invitrogen, Cat# A21422) diluted in the blocking solution. The stained slides were washed with PBST and mounted using VECTASHIELD Vibrance (Vector, Cat# H-1700-10).

To prevent any potential bias, at least 5–6 images were acquired across the whole slide with cellSens standard software (Olympus, v4.1) using a 40×/0.95 objective lens of a BX53 microscope equipped with a DP23M camera and a U-LGPS illuminant (EVIDENT). We manually measured at least ~500 track’s length of continuous red (IdU) and green (CldU) fibers (progressing replication forks) in each sample using Fiji (NIH, v1.54). Fork speed (kb/min) was calculated using the formula (1 μm = 3.5 kb) according to the length (μm) converted from the pixel value and labeling time. Each experiment was independently repeated at least twice. For each sample, 100 or 150 DNA fibers were randomly selected and measured in each independent experiment, and the pooled 300 fibers are shown as superplots. White dots indicate the mean value of independent biological replicates, and black bars indicate the mean of the pooled fiber measurements. Statistical differences were determined using ordinary one-way ANOVA followed by Tukey’s multiple comparison test on mean value of pooled population.

### Long-term time-to-progression assay

For the long-term cell proliferation assay, a total of 8 × 10^4^ cells were plated in 6-cm plates with or without 0.1 μM 4OHT for 14 days. The cells were collected and counted every 3–4 days. The daily growth rate (*μ*) was calculated according to the formula *μ*  =  *(ln N – ln N´) / t*, where *N* is the number of cells in the current week, *N´* is the number of cells plated in the previous week, and *t* is the culture day. Based on the daily growth rate (*μ*) calculated at every split time, the total current cell number (*Nx*) was estimated according to the formula *ln Nx*  =  *ln N*_*0*_  +  μ * T, where *N*_0_ is the number of cells plated at the initial split point and *T* is the total number of incubation days.

### Immunoprecipitation

For endogenous SMARCAL1 immunoprecipitation, 6 × 10^6^ cells were used. After harvesting with trypsin, cells were lysed in 1.5 mL of KCl-NP40 buffer (50 mM Tris-HCl [pH 7.4], 2 mM MgCl₂, 100 mM KCl, 1% Nonidet-P40, and 5% glycerol, 1 mM DTT, cOmplete [Roche, Cat#04693132001], and PhosSTOP [Roche, Cat#04906837001]). Lysates were sonicated at 30% amplitude with a 10 s-ON/10 s-OFF cycle for a total of 1 min ON, repeated twice. After collecting 0.5% input, 250 U of Benzonase (Sigma, Cat#E1014) and 1 μL of Dynabeads Protein A (Invitrogen, Cat#10001D; as pre‑cleaning) were added and rotated for 2 h at 4 °C. The reaction was terminated by adding 1 mM EDTA, and insoluble fraction and beads were removed by centrifugation at 20,000 × *g* for 15 min. After adjusting the volume, SMARCAL1 antibody (1:200, Cell Signaling Technology, Cat#44717) and an equal amount of control rabbit IgG (Cell Signaling Technology, Cat#2729) were added and incubated for 16–20 h at 4 °C with rotation. Immune complexes were captured using 5 μL Dynabeads per 1 mL lysate for 2 h. After magnetic removal of the supernatant, the beads were transferred to a new tube and washed five times with KCl-NP40 buffer. Bound proteins were eluted by denaturation in sampling buffer as described in the “Western blot analysis” section.

For exogenous FLAG-PrimPol immunoprecipitation, 1 × 10^7^ 293FT cells transfected with pCMV6‑PrimPol-myc-FLAG and/or pCMV6‑myc-SMARCAL1 were used. After harvesting by trypsinization, 0.5% input was collected, and the remaining cells were lysed in 0.5 mL of NaCl-NP40 buffer (50 mM Tris-HCl [pH 8.0], 2 mM MgCl_2_, 150 mM NaCl, 1% Nonidet-P40, 1 mM DTT, cOmplete, and PhosSTOP). Lysates were sonicated at 30% amplitude with a 10 s ON/10 s OFF cycle for a total ON time of 1 min, repeated twice. Benzonase (250 U) and 1 μL of Dynabeads Protein G (Invitrogen, Cat#10003D; as pre‑cleaning) were added, and the lysates were rotated for 2 h at 4 °C. The reaction was terminated by adding 1 mM EDTA, followed by centrifugation at 20,000 × *g* for 15 min to remove insoluble fraction and beads. After volume adjustment, FLAG antibody (1:60, MBL, Cat#M185‑3 L) was added and the lysates were incubated for 16–20 h at 4 °C with rotation. Immune complexes were captured using 5 μL of Dynabeads per 1 mL lysate for 2 h. After magnetic removal of the supernatant, beads were transferred to a new tube and washed three times with NaCl-NP40 buffer. Bound proteins were eluted by denaturation in sampling buffer.

### RNA extraction and real-time PCR

After siRNA transfection, total RNA was purified using an RNeasy Mini Kit (QIAGEN, Cat# 74104) according to the manufacturer’s instructions. RNA (1 μg) was reverse-transcribed using SuperScript™ IV VILO™ Master Mix (Invitrogen, Cat#11766050) according to the manufacturer’s instructions. SYBR Green real-time PCR was performed using the THUNDERBIRD^®^ Next SYBR™ qPCR Mix (TOYOBO, Cat# QPX-201). The following primers were used at 250 nM: Beta Actin primer-F: 5´-ATCAAGATCATTGCTCCTCCTGAG, Beta Actin primer-R: 5´-CTGCTTGCTGATCCACATCTG, Rev3 primer-F: 5´-GCAGGACCCAACTCTGAGAC, Rev3 primer-R: 5´-ATAGTGCCTTTCCGCCCTTC, ZRANB3 primer-F: 5´-GCTGCCAAGGAATTGAATACC, ZRANB3 primer-R: 5´-CCTTTACAGCACCTGCCTTGG.

PCR was performed on a QuantStudio3 thermocycler (Applied Biosystems). The exported data were analyzed using the Design & Analysis 2 (DA2) software (Thermo Fisher, v2.8.0) with the comparative Ct (ΔΔCt) method to quantify mRNA expression.

### Propidium iodide (PI) stain and flow cytometry (FCM)

A total of 8 × 10⁵ cells were collected by trypsinization and washed once with PBS. Cells were resuspended in 100 μL of PBS containing 0.1% Triton X‑100 and 300 μg/mL RNase A, and incubated for 10 min at room temperature. After centrifugation at 800 × *g* and washing with PBS, cells were resuspended in 100 μL of PBS containing 10 μg/mL PI and incubated for 10 min at room temperature. Cells were washed again with PBS and finally resuspended in 1 mL PBS for analysis. FCM was performed using a BD FACSCanto II (BD Bio) according to manufacturer’s instructions. Data were analyzed using BD FACSDiva software. Typical flow cytometry gating was applied to exclude obvious debris.

### Bacterial expression and protein purification

For His-tag PrimPol purification, BL21 cells were transformed with pETUA-His‑PrimPol, and expanded into 30 mL of LB medium and grown for 5 h at 30 °C. Cultures were then further expanded into 2 L of LB medium and induced with 1 mM IPTG, followed by incubation for 19 h at 19 °C. Cells were harvested by centrifugation at 2330 × *g* for 5 min and resuspended in 40 mL of Buffer A (50 mM Tris-HCl [pH 8.0], 1 M NaCl, and 10% glycerol supplemented with 1 mM PMSF, 2 mM 2‑mercaptoethanol, 10 mM imidazole, and cOmplete). Cells were sonicated at 40% amplitude with a 30 s ON/30 s OFF cycle for a total ON time of 15 min, and lysates were clarified by centrifugation at 20,000 × *g* for 1 h. The supernatant was incubated with 1 mL Ni‑NTA agarose (Wako, Cat#143‑09763) for 3 h with rotation. Beads were packed into a spin column (Bio‑Rad, Cat#732‑6204) and washed five times with 500 μL Buffer A, followed by three washes with 500 μL Buffer A containing 20 mM imidazole. Beads were subsequently washed five times with 500 μL Buffer B (50 mM Tris-HCl [pH 8.0] and 10% glycerol, supplemented with 1 mM PMSF, 2 mM 2‑mercaptoethanol, and cOmplete) containing 50 mM NaCl. Bound proteins were eluted with 750 μL Buffer B containing 50 mM NaCl and 500 mM imidazole by gravity flow for 30 min. The elution was loaded into a HiTrap Heparin HP column (Cytiva, Cat#17040601). The column was washed three times with 1 mL Buffer B containing 50 mM NaCl, followed by three washes with 1 mL Buffer B containing 100 mM NaCl. His‑PrimPol was eluted with 4 mL Buffer B containing 1 M NaCl, and fractions were collected. An equal volume of glycerol was added to each fraction. The appropriate fraction was selected, and the concentration was determined by SDS-PAGE followed by SYPRO Ruby staining (Invitrogen, Cat#S12001).

For GST-conjugated PrimPol 250-260aa peptides purification, BL21 cells were transformed with pGEX-2TK-GST-PrimPol peptides, expanded into 100 mL of LB medium, and induced with 1 mM IPTG, followed by incubation for 4 h at 37 °C. Bacterial pellets were washed with PBS, resuspended in extraction buffer (20 mM Hepes [pH 7.6], 500 mM NaCl, 0.5 mM EDTA, 10% glycerol, 0.5% Nonidet-P40, cOmplete, 1 mM DTT, and 1 mM PMSF) and sonicated. Following centrifugation, the cleared supernatant was incubated with glutathione-Sepharose beads (GE Healthcare, Cat# 17-0756-01) for 2 h at 4 °C. Beads were washed three times with wash buffer I (20 mM Tris-HCl [pH 8.0], 150 mM NaCl, 1% Triton-X100, 1 mM DTT, and 1 mM PMSF), once with wash buffer II (100 mM Tris-HCl [pH 7.5], 100 mM NaCl, 5% Glycerol, 1 mM DTT, and 1 mM PMSF), and bound proteins were recovered with elution buffer (100 mM Tris-HCl [pH 7.5], 100 mM NaCl, 5% Glycerol, and 42.3 mM glutathione). Purified proteins were dialyzed into wash buffer II.

### Chk1 kinase assay and Phos-Tag SDS-PAGE

Bioactive Chk1 was purchased (SignalChem Cat#C47-10H). Chk1 (100 ng) was mixed with 100 ng of purified His-PrimPol^Full-length^ in Kinase buffer (5 mM MOPS [pH7.0], 2.5 mM β-Glycerol phosphate, 1 mM EGTA, 0.4 mM EDTA, 5 mM MgCl_2_, and 0.05 mM DTT) supplemented with or without 250 μM of ATP and 10 μM Chk1i. The mixture was incubated for 30 min at 37 °C, and reaction was terminated by addition of Sampling buffer, followed by denaturation at 95 °C. Subsequently, the lysate was resolved by 8% polyacrylamide gel supplemented with 50 μM of Phos-Tag (Wako, Cat# 300-93523) and 100 μM MnCl_2_. After PAGE, the gel was washed twice with 5 mM EDTA solution, and WET transfer was performed with 0.1% SDS supplemented transfer buffer. Samples from kinase assays for GST-PrimPol^250-260^ fragments were prepared as described above, subjected to standard SDS-PAGE.

### Proximity ligation assay (PLA)

Following treatment, S-phase cells were pulse-labeled with 10 μM EdU (Invitrogen, Cat#A10044) for 10 min, washed twice with PBS. Cells were then pre-extracted with 0.05 % Triton X-100 in PBS for 5 min on ice, washed twice with PBS and fixed with 4% paraformaldehyde for 10 min at RT. Cells were washed twice with PBS before permeabilization with 0.25% Triton X-100 in PBS for 10 min at RT. Following permeabilization, cells were washed 3 times with PBS and processed with Click-iT Plus EdU Alexa Fluor 488 Imaging Kit (Invitrogen, Cat#C10637) according to the manufacturer’s instructions. Cells were then washed 3 times in PBS, blocked in 3 % BSA with 0.05% Tween 20 in PBS (T-BSA) for 1 h at RT, and were incubated with primary antibody at 4 °C overnight. The following primary antibodies were used: anti-CCDC111 (10-B3) (1:2000, # MA5-32899, Invitrogen) and anti-phospho-PrimPol Ser255 (1:5000, custom antibody raised against LERLG[pS]AEQSSPDLS, Merck) in 5% Blocking One-P (Nacalai Tesque, 05999-84). After incubation, cells were washed three times with PBS, PLA was performed using Duolink In Situ PLA kits (Sigma-Aldrich, Cat#DUO92001, #DUO92005, #DUO92008), according to the manufacturer’s protocol. Nuclei were stained with 1 µg/mL DAPI in PBS for 5 min at RT, washed again with PBS, then slides were mounted with VECTASHIELD PLUS Antifade Mounting Medium (Vector Laboratories, Cat#H-1900). Images were acquired with a fluorescence microscope Celldiscoverer 7 (Zeiss), and analyses were performed using Zen (Zeiss, v2.6). Foci numbers of randomly selected 200 cells were pooled and totally 600 cells are shown as a super plot. White dot represents mean value of each biological replicate, and black bar represents mean value of the pool. Statistical differences were determined using Repeated Measures one-way ANOVA Tukey’s multiple comparison test.

### Acquiring the Cancer Genome Atlas data

Mutational profiles of ATR, PrimPol, and SMARCAL1 were analyzed using cBioPortal (https://www.cbioportal.org/). Simple nucleotide variation data of TCGA-LUAD (618 MAF files) were downloaded from the Genomic Data Commons (NIH, https://portal.gdc.cancer.gov/) using the manifest file, which includes file UUIDs and download paths, and the GDC Data Transfer Tool (NIH, v2.3.0). Smoking profile, RNA expression levels of ATR, PrimPol, and SMARCAL1, and KRAS mutational data from TCGA-LUAD samples were downloaded from UCSC Xena (UCSC, https://xena.ucsc.edu/).

The low and high expression groups for ATR, PrimPol, and SMARCAL1 were defined as follows: the dataset was divided into two groups (High and Low) such that the ratio of average expression levels in the low and high groups was minimal.

### Mutation count from MAF Files with R

To quantify the total number of single-nucleotide variants (SNVs) and their subtypes per sample, we analyzed the Mutation Annotation Format (MAF) files using R (v 4.4.1). All MAF files were merged into a single data table using the *data.table* package. We extracted the mutation types from the *Variant_Type* column and sample identifiers from the *Tumor_Sample_Barcode* column. SNVs were aggregated using a*. N* operator function in the *data.table* package, and the counts of individual SNV types (e.g., SNP, INS, and DEL) were tabulated using a *dcast()* function. The extracted SNV counts were unified with the RNA expression profile using the *VLOOKUP()* function of Excel (Microsoft) according to the sample barcode to omit incomplete datasets due to the lack of information.

### Extraction of mutational signatures using *MutationalPatterns* with R

We performed mutational signature analysis using the *MutationalPatterns* package in R^71^. First, all somatic SNVs were extracted from the MAF files of each classified group (repriming-prone and Polζ-prone). Indel and non-SNV variants were filtered out by selecting entries where both the reference and alternate alleles were single bases. The cleaned MAF entries were converted into GRanges objects and annotated with the reference human genome hg38 using the *BSgenome.Hsapiens.UCSC.hg38* package. To characterize mutational patterns, the trinucleotide context of each SNV was retrieved, and a 96-feature single-base substitution (SBS96) mutational profile was constructed using the *mut_matrix()* function. The reference COSMIC v3.2 SBS signatures (for GRCh38) were obtained using a *get_known_signatures()* function. To identify the most relevant mutational signatures in our samples, we computed the cosine similarity between the observed mutational profile and each reference signature using the *cos_sim_matrix()* function. Signatures with a cosine similarity ≥0.65 were selected for downstream analysis. The contributions of the selected signatures to the mutational profiles were then estimated using the *fit_to_signatures() function*. Finally, to assess the fidelity of signature fitting, we reconstructed the mutational profiles using the estimated signature contributions and evaluated the cosine similarity between the original and reconstructed profiles. Cosine similarity >0.85 was considered a reliable signature decomposition.

### Quantification and statistical analysis

Statistical differences in scatter dot plots of the DNA fiber assay or S1 fiber assay were determined using an ordinary one-way ANOVA Tukey’s multiple comparison test. Statistical differences in the boxplot of qPCR presented as the mean ± SD for technical replicates were determined using an ordinary one-way ANOVA Dunnett’s multiple comparison test. Statistical differences in scatter dot plots of the PLA were determined using an RM one-way ANOVA Tukey’s multiple comparison test. Statistical differences in the violin plot of the number of variants or RNA expression levels presented as the median were determined using a nonparametric Mann-Whitney U *t* test. All statistical analyses were performed using Prism 10 (v10.4.2) software, and *p*  <  0.05 was considered as statistically significant.

### Reporting summary

Further information on research design is available in the Nature Portfolio Reporting Summary linked to this article.

## Supplementary information

Supplementary Information

Reporting Summary

Transparent Peer Review file

## Source data

Source Data

## Data Availability

All data supporting the findings of this study are provided in the main manuscript and its Supplementary Information files. Source data are provided with this paper.
