# Supplementary material for: PrimPol toggled by ATR modulates fork slowing and repriming under oncogenic KRAS-induced replication stress
Source: Nat Commun. 2026 Oct 1;17:11277. doi: 10.1038/s41467-026-77585-y (PMC13720195; doi:10.1038/s41467-026-77585-y)
Supplement: Supplementary file 1 [file 41467_2026_77585_MOESM1_ESM.pdf]

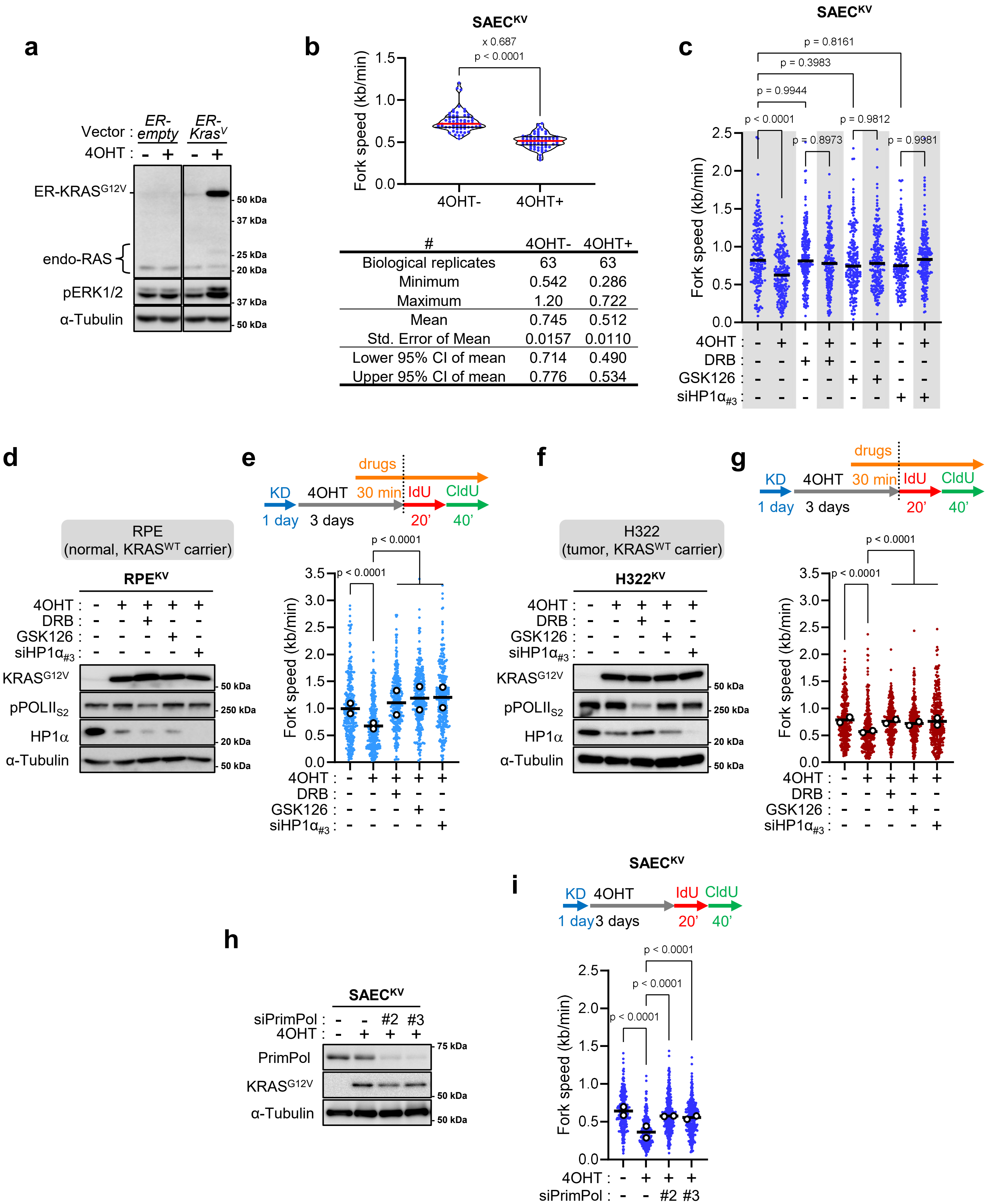

#	4OHT-	4OHT+
Biological replicates	63	63
Minimum	0.542	0.286
Maximum	1.20	0.722
Mean	0.745	0.512
Std. Error of Mean	0.0157	0.0110
Lower 95% CI of mean	0.714	0.490
Upper 95% CI of mean	0.776	0.534

Supplementary Figure 1. PrimPol is required for active fork slowing during an acute K-HaRS response

a Western blot analysis of SAEC harboring estrogen receptor (ER) or ER-conjugated $KRAS^{G12V}$ treated with or without 0.1 μM 4OHT. **b** Scatter dot plot and median values of DNA fibers in $SAEC^{KV}$ treated with 4OHT for 3 days. Data were compiled from 63 independent DNA fiber experiments. Two-sided paired t test. **c** Representative scatter dot plot and mean of DNA fiber in $SAEC^{KV}$ treated with 100 μM DRB, 2.5 μM GSK126, or 1 nM siRNA targeting HP1α. Samples indicated by gray-boxes are also shown in Figure (**1b**). **d** Western blot analysis of RPE harboring 4OHT-inducible $KRAS^{G12V}$ (RPE^{KV}) treated with 100 μM DRB, 2.5 μM GSK126, or 1 nM siRNA targeting HP1α. **e** Scatter dot plot and mean of DNA fiber in RPE^{KV}, performed as in the top scheme. n = 2 biological replicates, 150 fibers per replicate. **f** Western blot analysis of H322 harboring 4OHT-inducible $KRAS^{G12V}$ ($H322^{KV}$) treated with 100 μM DRB, 2.5 μM GSK126, or 1 nM siRNA targeting HP1α. **g** Scatter dot plot and mean of DNA fiber in $H322^{KV}$, performed as in the top scheme. n = 2 biological replicates, 150 fibers per replicate. **h** Western blot analysis of $SAEC^{KV}$ transfected with two independent 1 nM siRNA (#2 and #3) targeting PrimPol. **i** Scatter dot plot and mean of DNA fiber in $SAEC^{KV}$ with or without $KRAS^{G12V}$ expression, performed as in the top scheme. n = 2 biological replicates, 150 fibers per replicate. **c, e, g, i** one-way ANOVA Tukey's test. All source data are provided as a Source Data file.

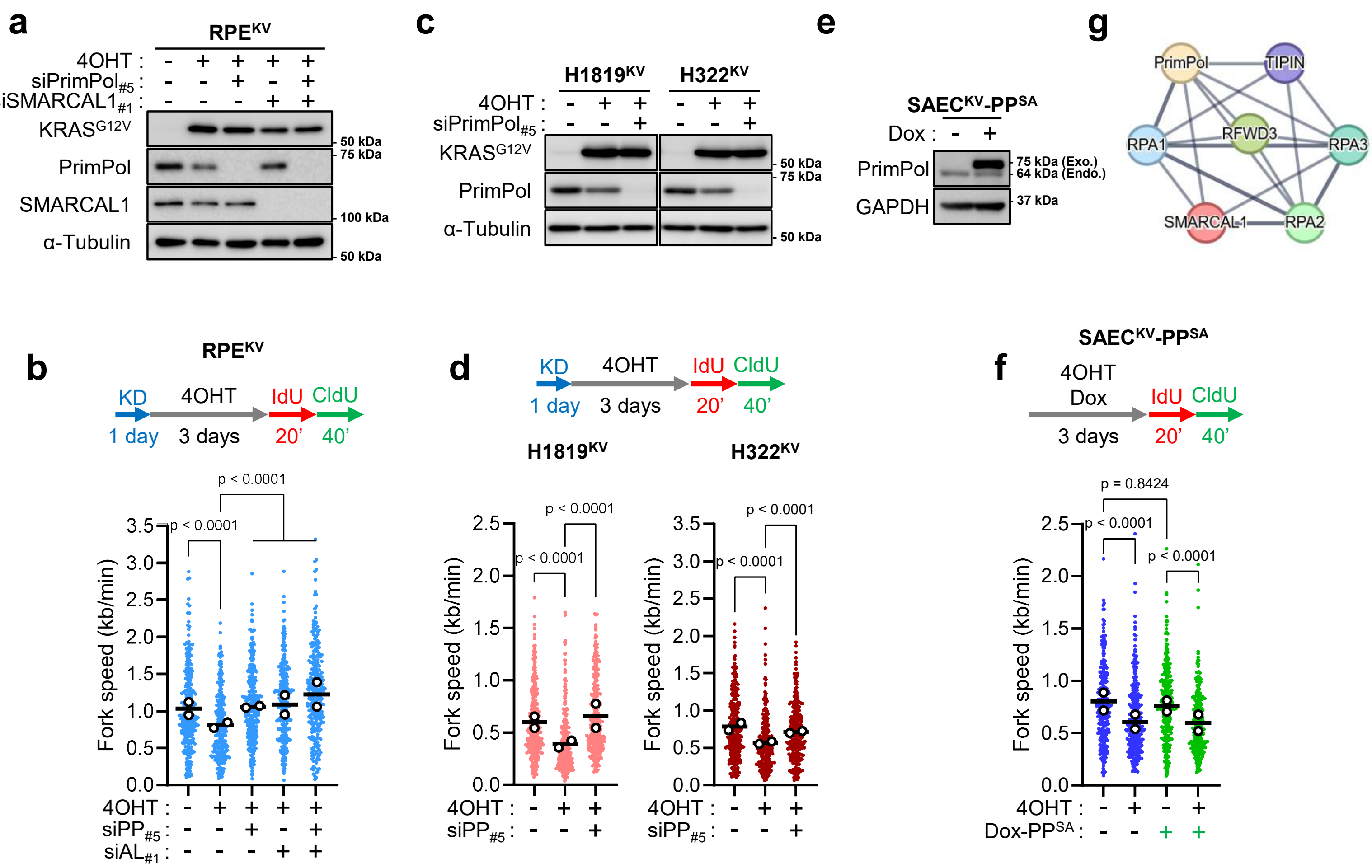

Supplementary Figure 2. The unphosphorylated form of PrimPol acts epistatically with SMARCAL1 to promote fork slowing

a Western blot analysis of RPE^{KV} transfected with 1 nM siRNA targeting PrimPol and/or SMARCAL1. **b** Scatter dot plot and mean of DNA fiber in RPE^{KV} transfected with 1 nM siRNA targeting PrimPol and/or SMARCAL1, performed as in the top scheme. n = 2 biological replicates, 150 fibers per replicate. **c** Western blot analysis of H1819^{KV} and H322^{KV} transfected with 1 nM siRNA targeting PrimPol. **d** Scatter dot plot and mean of DNA fiber in H1819^{KV} and H322^{KV}, performed as in the top scheme. n = 2 biological replicates, 150 fibers per replicate. **e** Western blot analysis of SAEC^{KV}-PP^{SA} treated with 1 μg/mL of doxycycline. **f** Scatter dot plot and mean of DNA fiber in SAEC^{KV}-PP^{SA} treated with 1 μg/mL of Dox, performed as in the top scheme. n = 2 biological replicates, 150 fibers per replicate. **g** Physical subnetwork predicted by STRING (https://string-db.org/). The line thickness indicates the strength of data confidence. **b, d, f** one-way ANOVA Tukey's test. All source data are provided as a Source Data file.

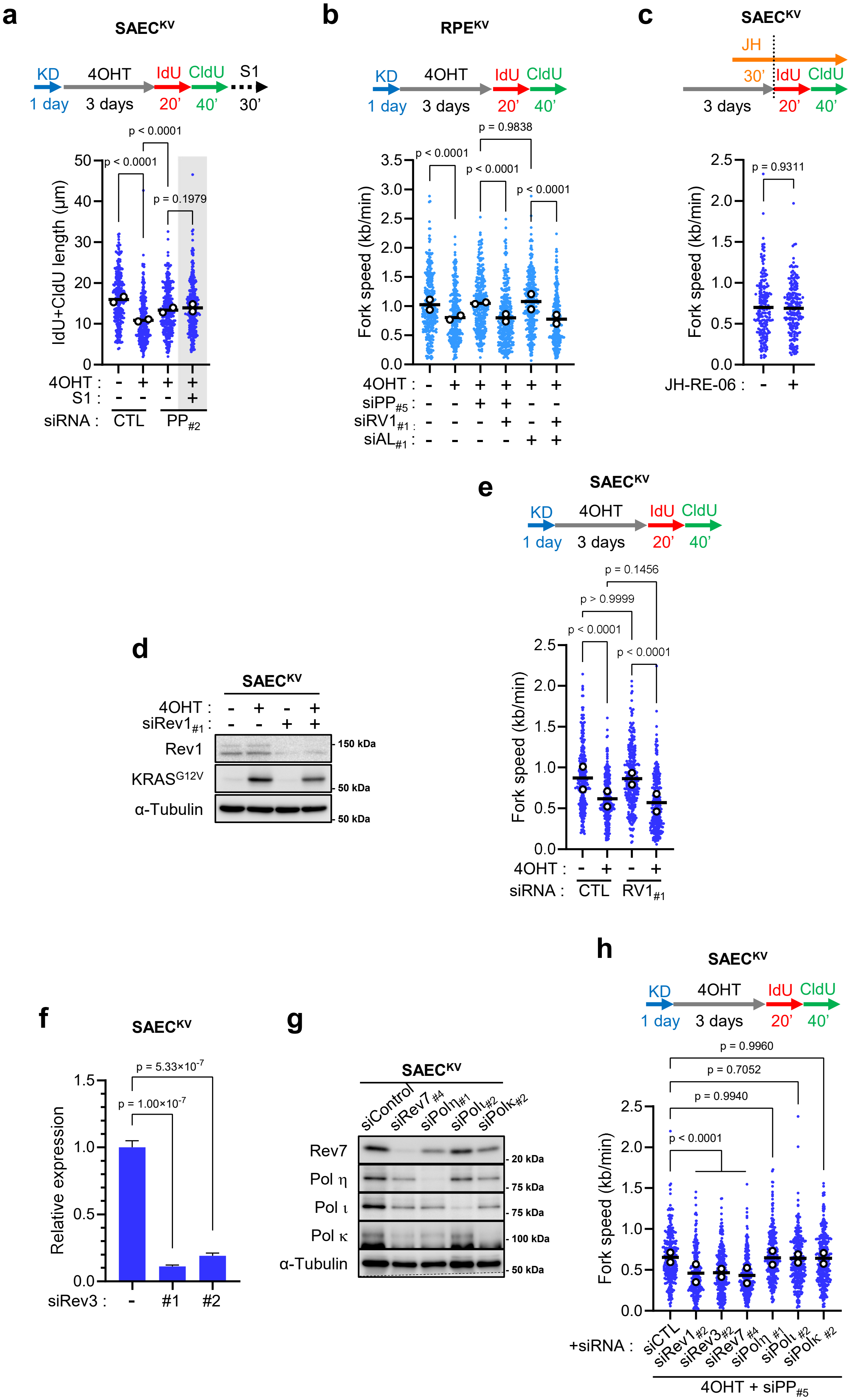

a
SAEC^{KV}
KD 1 day
4OHT 3 days
IdU 20'
CldU 40'
S1 30'
p < 0.0001
p < 0.0001
p = 0.1979
IdU+CldU length (µm)
4OHT :
S1 :
siRNA : CTL PP$_{\#2}$
b
RPE^{KV}
KD 1 day
4OHT 3 days
IdU 20'
CldU 40'
p = 0.9838
p < 0.0001
p < 0.0001
p < 0.0001
Fork speed (kb/min)
4OHT :
siPP$_{\#5}$:
siRV1$_{\#1}$:
siAL$_{\#1}$:
c
SAEC^{KV}
JH 30'
IdU 20'
CldU 40'
3 days
p = 0.9311
Fork speed (kb/min)
JH-RE-06 :
d
SAEC^{KV}
4OHT :
siRev1$_{\#1}$:
Rev1
KRAS^{G12V}
α-Tubulin
150 kDa
50 kDa
50 kDa
e
SAEC^{KV}
KD 1 day
4OHT 3 days
IdU 20'
CldU 40'
p = 0.1456
p > 0.9999
p < 0.0001
p < 0.0001
Fork speed (kb/min)
4OHT :
siRNA : CTL RV1$_{\#1}$
f
SAEC^{KV}
p = 5.33×10^{-7}
p = 1.00×10^{-7}
Relative expression
siRev3 : - #1 #2
g
SAEC^{KV}
siControl
siRev7$_{\#4}$
siPolη$_{\#1}$
siPolι$_{\#2}$
siPolκ$_{\#2}$
Rev7
Pol η
Pol ι
Pol κ
α-Tubulin
20 kDa
75 kDa
75 kDa
100 kDa
50 kDa
h
SAEC^{KV}
KD 1 day
4OHT 3 days
IdU 20'
CldU 40'
p = 0.9960
p = 0.7052
p = 0.9940
p < 0.0001
Fork speed (kb/min)
+siRNA : siCTL siRev1$_{\#2}$ siRev3$_{\#2}$ siRev7$_{\#4}$ siPolη$_{\#1}$ siPolι$_{\#2}$ siPolκ$_{\#2}$
4OHT + siPP$_{\#5}$

Supplementary Figure 3. The dysregulation of PrimPol- and SMARCAL1-mediated active fork slowing triggers Rev1-Polζ-dependent DNA replication

a Scatter dot plot and mean of S1 DNA fiber in $SAEC^{KV}$ transfected with 1 nM siRNA targeting PrimPol, performed as in the top scheme. Cells were permeabilized and subsequently treated with 10 U/mL S1 nuclease for 30 min. n = 2 biological replicates, 150 fibers per replicate. **b** Scatter dot plot and mean of DNA fiber in RPE^{KV} transfected with 1 nM siRNA targeting PrimPol, SMARCAL1, and/or Rev1, performed as in the top scheme. n = 2 biological replicates, 150 fibers per replicate. **c** Representative scatter dot plot and mean of DNA fiber in $SAEC^{KV}$ treated with 10 μM JH-RE-06, performed as in the top scheme. n = 200. Two-sided Mann-Whitney test. **d** Western blot analysis of $SAEC^{KV}$ transfected with 1 nM siRNA targeting Rev1. **e** Scatter dot plot and mean of DNA fiber in $SAEC^{KV}$, performed as in the top scheme. n = 2 biological replicates, 150 fibers per replicate. **f** qPCR analysis of $SAEC^{KV}$ transfected with two independent 1 nM siRNA (#1 and #2) targeting Rev3. Relative expression levels were calculated using ΔΔCT method with ACTB as an endogenous control. Data are shown as mean ± SD of three technical replicates. Ordinary one-way ANOVA followed by Dunnett's multiple comparisons test. **g** Western blot analysis of $KRAS^{G12V}$ expressing $SAEC^{KV}$ transfected with 1 nM siRNA targeting PrimPol, Rev7, Polη, Polι, or Polκ. **h** Scatter dot plot and mean of DNA fiber in $SAEC^{KV}$, performed as in the top scheme. n = 2 biological replicates, 150 fibers per replicate. **a-b, e, h** one-way ANOVA Tukey's test. All source data are provided as a Source Data file.

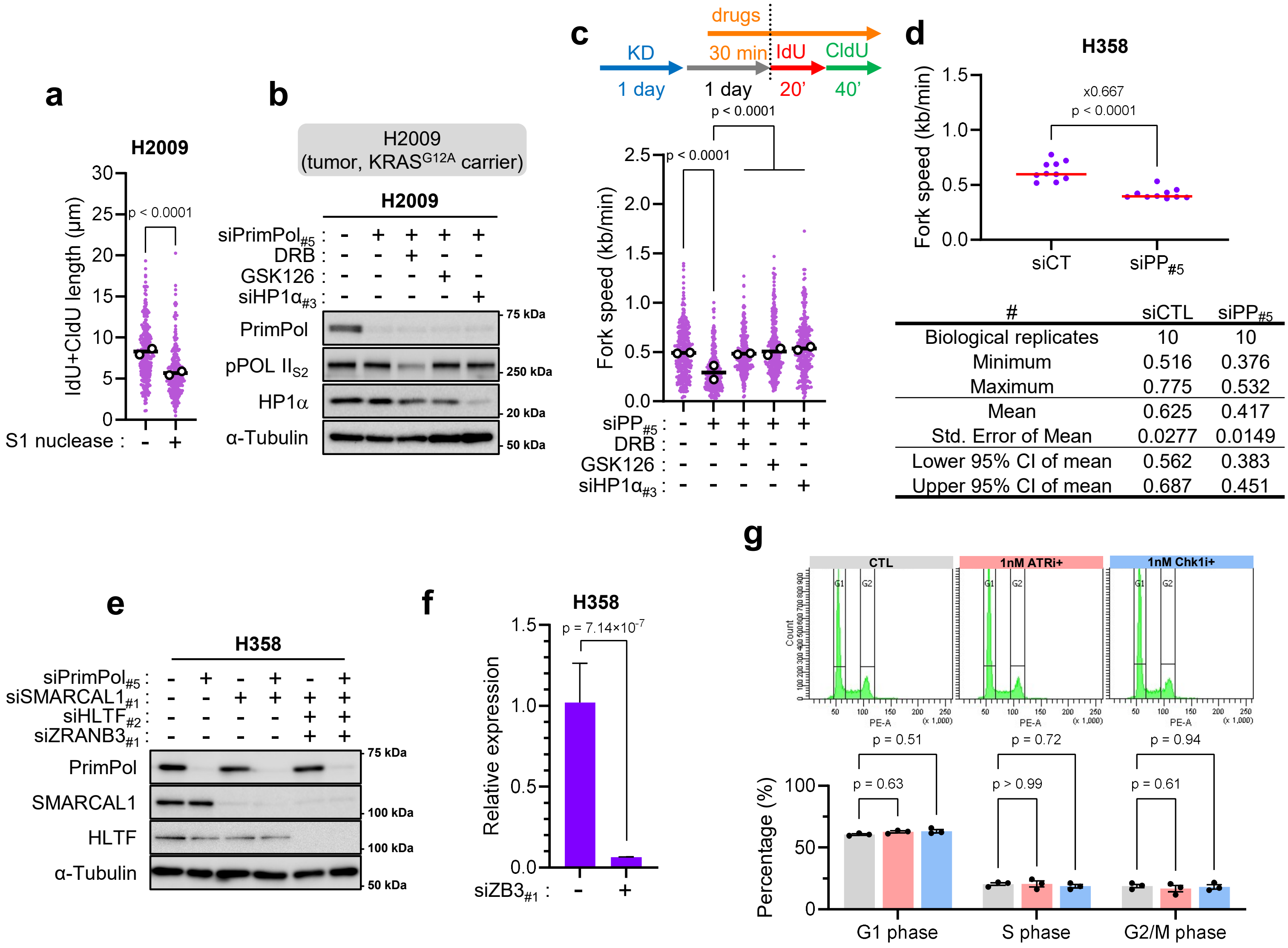

#	siCTL	siPP#5
Biological replicates	10	10
Minimum	0.516	0.376
Maximum	0.775	0.532
Mean	0.625	0.417
Std. Error of Mean	0.0277	0.0149
Lower 95% CI of mean	0.562	0.383
Upper 95% CI of mean	0.687	0.451

Supplementary Figure 4. ATR prioritizes PrimPol-dependent repriming during adapted/chronic K-HaRS

a Scatter dot plot and mean of S1 DNA fiber in H2009. Cells were permeabilized and subsequently treated with 10 U/mL of S1 nuclease for 30 min. n = 2 biological replicates, 150 fibers per replicate. **b** Western blot analysis of H2009 transfected with 1 nM siRNA targeting PrimPol and treated with 100 μM DRB, 2.5 μM GSK126, or 1 nM siRNA targeting HP1α. **c** Scatter dot plot and mean of DNA fiber in H2009, performed as in the top scheme. n = 2 biological replicates, 150 fibers per replicate. **d** Scatter dot plot and median values of DNA fibers in H358 transfected with or without 1 nM siRNA targeting PrimPol. Data were compiled from 10 independent DNA fiber experiments. Two-sided paired t test. **e** Western blot analysis of H358 transfected with 1 nM siRNA targeting PrimPol, SMARCAL1, and/or triple translocases (HLTF, ZRANB3, and SMARCAL1; 3KD), performed as in Figure (**4h**). **f** qPCR analysis of H358 transfected with 1 nM siRNA targeting ZRANB3. Relative expression levels were calculated using ΔΔCT method with ACTB as an endogenous control. Data are shown as mean ± SD of three technical replicates. Ordinary one-way ANOVA followed by Dunnett's multiple comparisons test. **g** Flow Cytometry analysis of H358 cells treated with 1 nM ATRi (berzosertib) or 1 nM Chk1i (rabusertib) for 24 hours. Representative cell cycle profiles are shown at the top, and quantification is shown as mean ± SEM of three biological replicates at the bottom. Ordinary two-way ANOVA followed by Tukey's multiple comparisons test. **a, c** one-way ANOVA Tukey's test. All source data are provided as a Source Data file.

(continued)

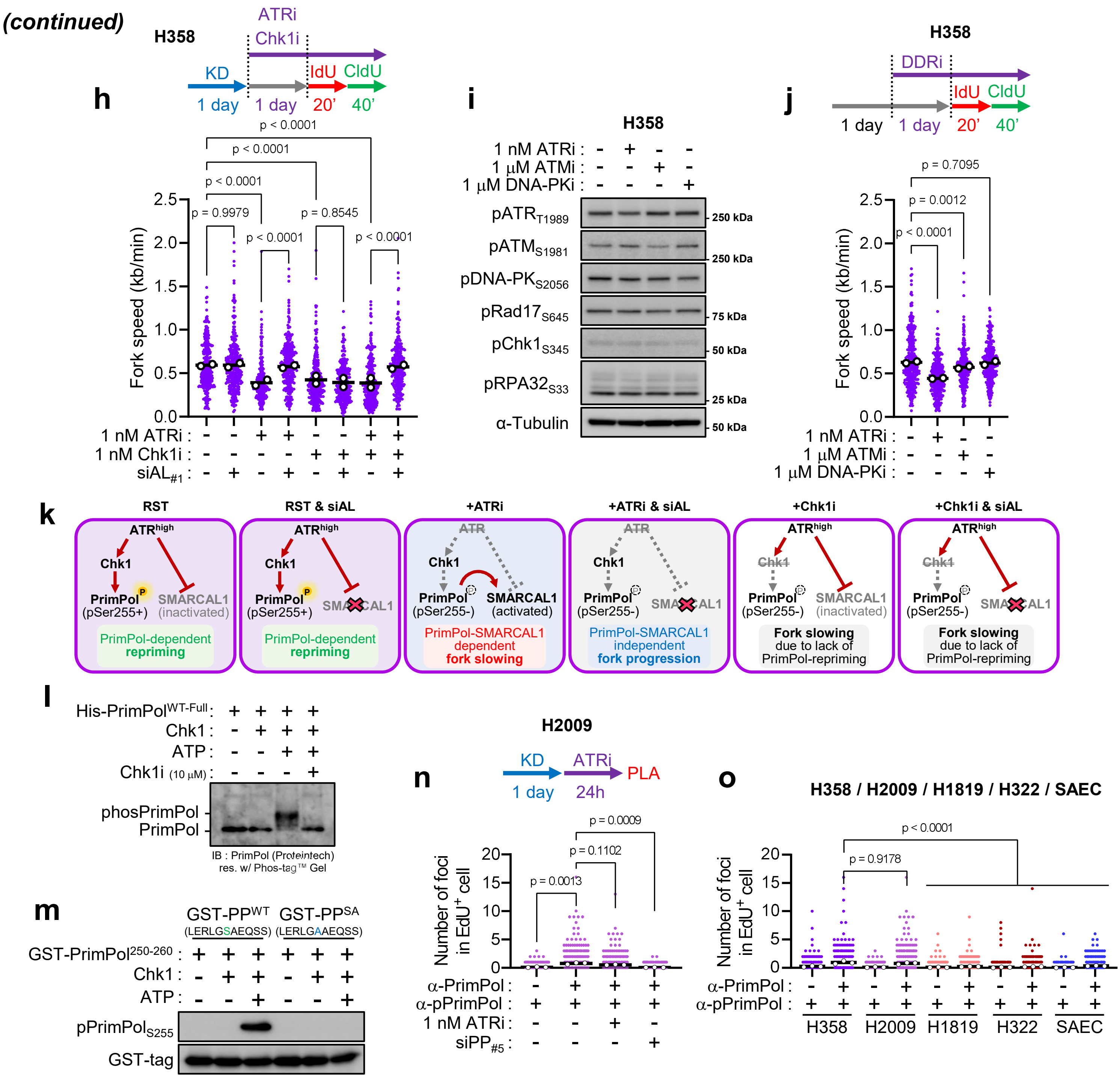

Supplementary Figure 4. (continued) ATR prioritizes PrimPol-dependent repriming during adapted/chronic K-HaRS

h Scatter dot plot and mean of DNA fiber in H358 treated with 1 nM ATRi (berzosertib) and/or 1 nM Chk1i (rabusertib) ± 1 nM siRNA targeting SMARCAL1, performed as in the top scheme. n = 2 biological replicates, 150 fibers per replicate. **i** Western blot analysis of H358 treated with 1 nM ATRi, 1 µM ATMi (KU55933), 1 µM DNA-PKi (NU7441) for 24 hours. **j** Scatter dot plot and mean of DNA fiber in H358, performed as in the top scheme. n = 2 biological replicates, 150 fibers per replicate. **k** Schematic illustration of K-HaRS tolerance module. Also see Figure (**4l**). **l** *In vitro* Chk1-PrimPol kinase assay. Full-length His-PrimPol was resolved by Phos-Tag SDS-PAGE and detected by a PrimPol antibody (Proteintech, Cat#29824-1-AP). **m** Validation of the phospho-Ser255 PrimPol antibody using GST-conjugated wild-type and S255A PrimPol 250-260 aa fragments in an *in vitro* kinase assay. **n** Scatter dot plot and mean values of proximity ligation assay (PLA) foci number per cell between intact PrimPol and phospho-Ser255 PrimPol in H2009 cells treated with 1 nM ATRi or 1 nM siRNA targeting PrimPol, as shown in the schematic above. n = 3 biological replicates, 200 cells per replicate. Tukey RM one-way ANOVA. **o** Scatter dot plot and mean values of PLA foci per cell between intact PrimPol and phospho-Ser255 PrimPol in indicated cells. Tukey RM one-way ANOVA. **h, j** one-way ANOVA Tukey's test. All source data are provided as a Source Data file.

a

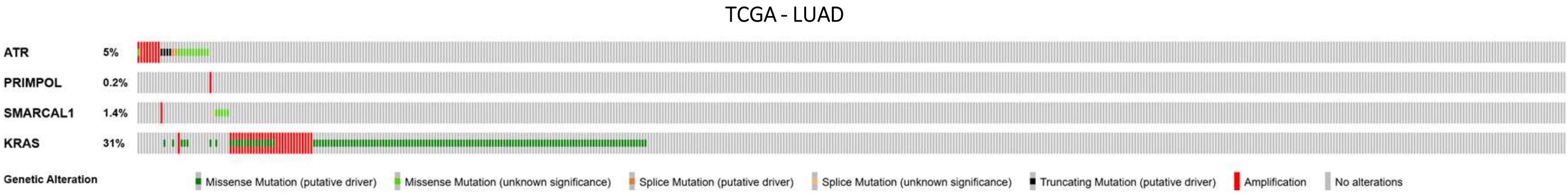

A	B	Neither	A Not B	B Not A	Both	Log2 Odds Ratio	p-Value	Tendency
PRIMPOL	KRAS	344	0	152	1	>3	0.308	N/A
ATR	KRAS	325	19	147	6	-0.518	0.513	N/A
SMARCAL1	KRAS	338	6	152	1	-1.432	0.682	N/A

b

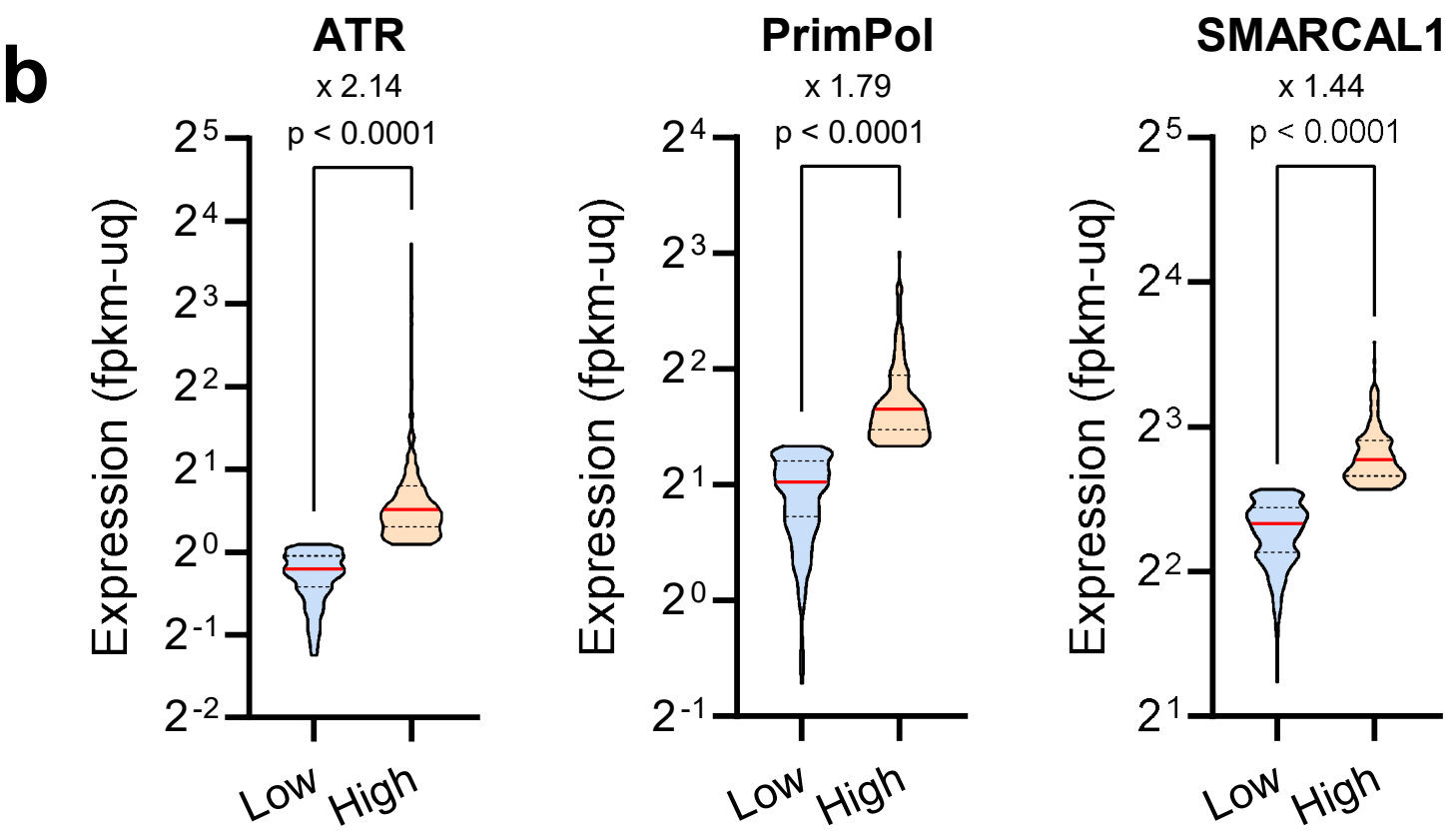

c

ATR	PrimPol	SMARCAL1	Class
High	High	Low	Repriming prone
Low	Low	Low	Polζ prone

d

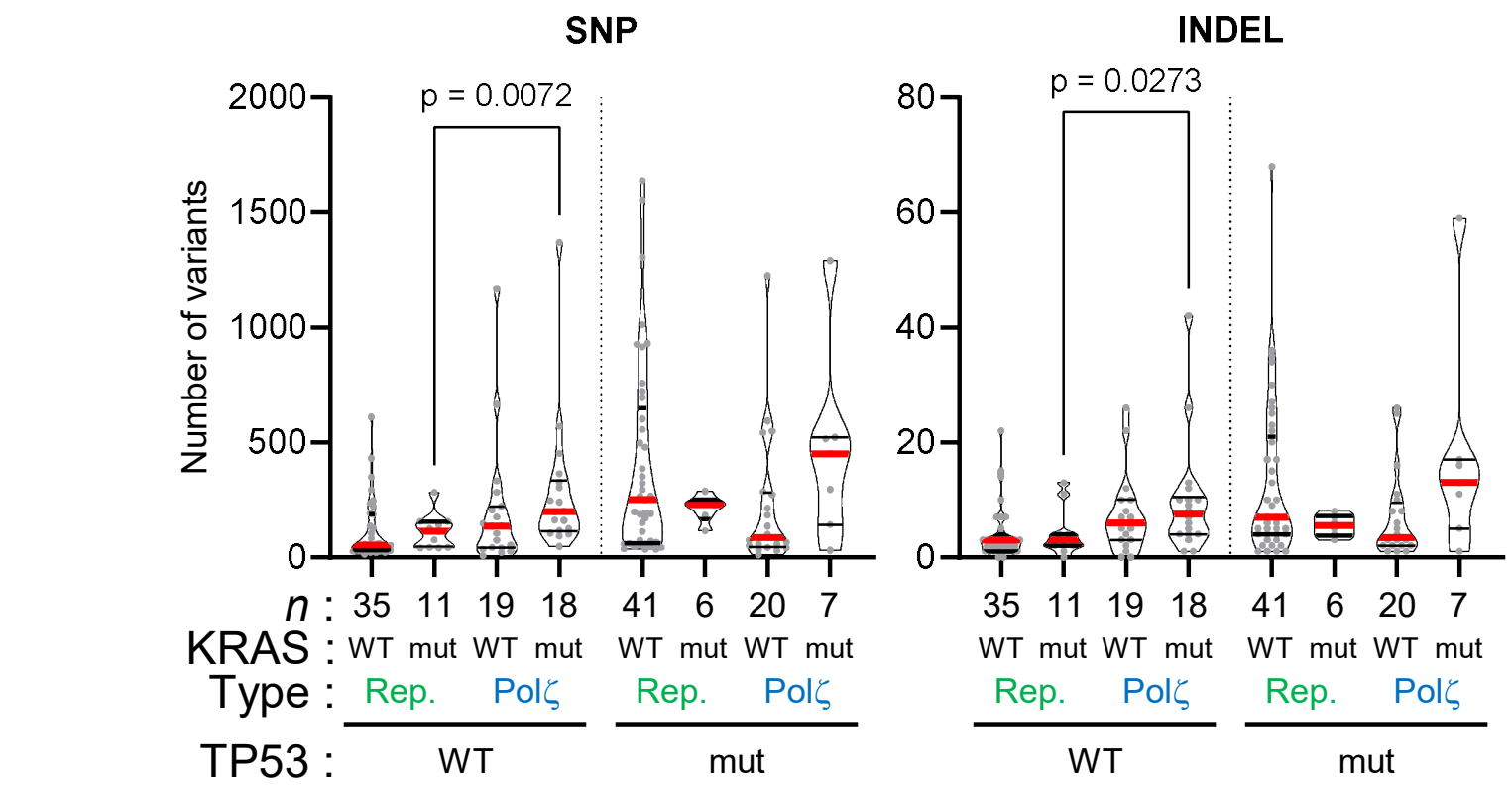

e

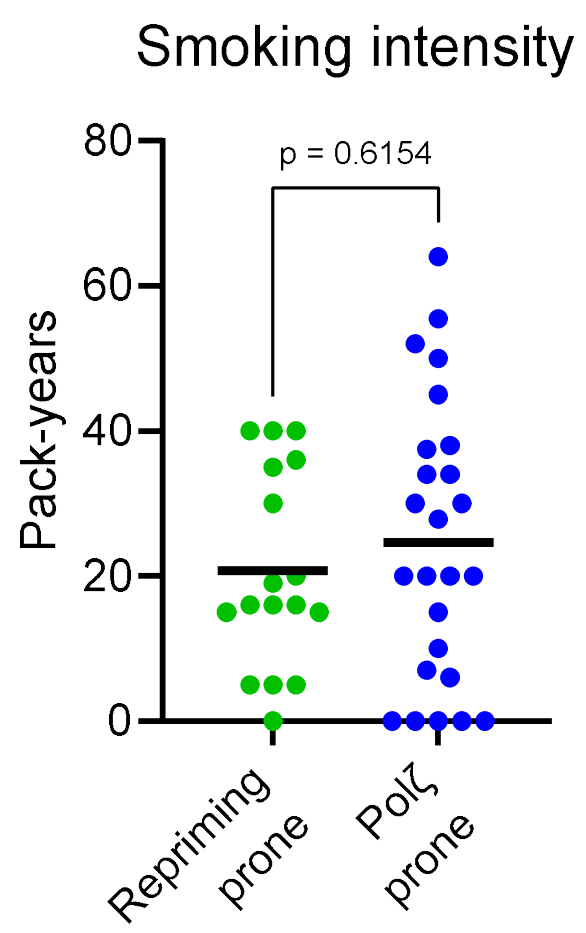

Supplementary Figure 5. ATR-dependent PrimPol regulation switches the K-HaRS response between repriming and active fork slowing

a Genetic alterations in ATR, PrimPol, SMARCAL1, and KRAS in the TCGA-LUAD cohort. Top, OncoPrint of TCGA-LUAD samples analyzed using cBioPortal (http://cbioportal.org). Bottom, co-occurrence and mutual exclusivity analysis between KRAS and the indicated genes. **b** RNA expression levels of ATR, PrimPol, and SMARCAL1 were downloaded from UCSC Xena (https://xena.ucsc.edu/). The low and high expression groups for ATR, PrimPol, and SMARCAL1 gene were defined as follows: the dataset was divided into two groups (High and Low) such that the ratio of average expression levels in the Low and High groups was minimized. Two-sided Mann-Whitney test. **c** The definition of “Repriming-prone” and “Polζ-prone” group. **d** Mutation profiling of the TCGA-LUAD cohort. The number of variations in SNP or INDEL upon Repriming-prone or Polζ-prone character divided by TP53 status. Two-sided Mann-Whitney test. See also Figure (**5h-j**). **e** Smoking intensity (Pack-year) in Repriming-prone and Polζ-prone groups. Two-sided Mann-Whitney test. Smoking profile was downloaded from UCSC Xena. All source data are provided as a Source Data file.
