# Supplementary material for: PrimPol toggled by ATR modulates fork slowing and repriming under oncogenic KRAS-induced replication stress
Source: Nat Commun. 2026 Oct 1;17:11277. doi: 10.1038/s41467-026-77585-y (PMC13720195; doi:10.1038/s41467-026-77585-y)
Supplement: Supplementary file 2 [file 41467_2026_77585_MOESM2_ESM.pdf]

nature portfolio

Corresponding author(s): Bunsyo Shiotani

Last updated by author(s): 2026/09/07

Reporting Summary

Nature Portfolio wishes to improve the reproducibility of the work that we publish. This form provides structure for consistency and transparency in reporting. For further information on Nature Portfolio policies, see our Editorial Policies and the Editorial Policy Checklist.

Statistics

For all statistical analyses, confirm that the following items are present in the figure legend, table legend, main text, or Methods section.

n/a	Confirmed	
☐	☒	The exact sample size (*n*) for each experimental group/condition, given as a discrete number and unit of measurement
☐	☒	A statement on whether measurements were taken from distinct samples or whether the same sample was measured repeatedly
☐	☒	The statistical test(s) used AND whether they are one- or two-sided<br>*Only common tests should be described solely by name; describe more complex techniques in the Methods section.*
☒	☐	A description of all covariates tested
☐	☒	A description of any assumptions or corrections, such as tests of normality and adjustment for multiple comparisons
☐	☒	A full description of the statistical parameters including central tendency (e.g. means) or other basic estimates (e.g. regression coefficient) AND variation (e.g. standard deviation) or associated estimates of uncertainty (e.g. confidence intervals)
☐	☒	For null hypothesis testing, the test statistic (e.g. *F*, *t*, *r*) with confidence intervals, effect sizes, degrees of freedom and *P* value noted<br>*Give P values as exact values whenever suitable.*
☒	☐	For Bayesian analysis, information on the choice of priors and Markov chain Monte Carlo settings
☒	☐	For hierarchical and complex designs, identification of the appropriate level for tests and full reporting of outcomes
☒	☐	Estimates of effect sizes (e.g. Cohen's *d*, Pearson's *r*), indicating how they were calculated

Our web collection on statistics for biologists contains articles on many of the points above.

Software and code

Policy information about availability of computer code

Data collection	LAS 4000 luminescent image analyzer (Fujifilm), BX53 microscope with DP23M camera & U-LGPS illuminant (EVIDENT), QuantStudio3 thermocycler (Applied Biosystems), BD FACSCanto II (BD Bio), Celldiscoverer 7 (Zeiss).
Data analysis	cellSens standard software (Olympus, v4.1), Fiji (NIH, v1.54), Design & Analysis 2 software (Thermo Fisher, v2.8.0), BD FACSDiva software (BD Bio), ZEN (Zeiss, v2.6) GDC Data Transfer Tool (NIH, v2.3.0), R (v 4.4.1), Prism 10 (v10.4.2).

For manuscripts utilizing custom algorithms or software that are central to the research but not yet described in published literature, software must be made available to editors and reviewers. We strongly encourage code deposition in a community repository (e.g. GitHub). See the Nature Portfolio guidelines for submitting code & software for further information.

Data

Policy information about availability of data

All manuscripts must include a data availability statement. This statement should provide the following information, where applicable:

- Accession codes, unique identifiers, or web links for publicly available datasets
- A description of any restrictions on data availability
- For clinical datasets or third party data, please ensure that the statement adheres to our policy

Simple nucleotide variation data of TCGA-LUAD (618 MAF files) were downloaded from the Genomic Data Commons (NIH, https://portal.gdc.cancer.gov/). Smoking profile, RNA expression levels of ATR, PrimPol, and SMARCAL1, and KRAS mutational data from TCGA-LUAD samples were downloaded from UCSC Xena

(UCSC, https://xena.ucsc.edu/).
Source data are provided with this paper.
This study does not report the original code. All software packages and the standard bioinformatics approaches used to analyze the data are described in the methods section.
Any additional information required to reanalyze the data reported in this paper is available from the lead contact upon request.

Research involving human participants, their data, or biological material

Policy information about studies with human participants or human data. See also policy information about sex, gender (identity/presentation), and sexual orientation and race, ethnicity and racism.

Reporting on sex and gender	N/A
Reporting on race, ethnicity, or other socially relevant groupings	N/A
Population characteristics	N/A
Recruitment	N/A
Ethics oversight	N/A

Note that full information on the approval of the study protocol must also be provided in the manuscript.

Field-specific reporting

Please select the one below that is the best fit for your research. If you are not sure, read the appropriate sections before making your selection.

[x] Life sciences [ ] Behavioural & social sciences [ ] Ecological, evolutionary & environmental sciences

For a reference copy of the document with all sections, see nature.com/documents/nr-reporting-summary-flat.pdf

Life sciences study design

All studies must disclose on these points even when the disclosure is negative.

Sample size	As sample size was determined according to standards in the field, no statistical method was used to predict sample size. Sample size was kept similar amongst different conditions. Sample size for all experiments shown (DNA fibers; n=100~150 in 2 or more independent experiments for tract lengths; pooled in n=300 super plots, Long-term time-to-progression assay; 3 independent experiments, qPCR; technical n=3 for siRNA validation) was chosen to obtainstatistical power. Sample size obtained from TCGA-LUAD was shown in figure.
Data exclusions	Outliers in the long-term time-to-progression assay were assessed using Grubbs' test (two-sided, $\alpha = 0.05$). No data were excluded for other assays.
Replication	All experiments were biologically repeated at least twice, with similar results. Typical phenotypes were repeatedly reproduced as experimental controls. Each data details were shown in figure legends.
Randomization	As we used cell lines grown under the same condition, sample randomization is not needed.
Blinding	As data was collected by imaging / objective measurements, blinding was not relevant to this study.

Reporting for specific materials, systems and methods

We require information from authors about some types of materials, experimental systems and methods used in many studies. Here, indicate whether each material, system or method listed is relevant to your study. If you are not sure if a list item applies to your research, read the appropriate section before selecting a response.

Materials & experimental systems

n/a	Involved in the study
☐	☒ Antibodies
☐	☒ Eukaryotic cell lines
☒	☐ Palaeontology and archaeology
☒	☐ Animals and other organisms
☒	☐ Clinical data
☒	☐ Dual use research of concern
☒	☐ Plants

Methods

n/a	Involved in the study
☒	☐ ChIP-seq
☐	☒ Flow cytometry
☒	☐ MRI-based neuroimaging

Antibodies

Antibodies used

anti-pan-RAS (1:1000, Santa Cruz, Cat# sc-166691)
anti-phospho POLII Ser2 (1:2000, Novus, Cat# NB100-1805)
anti-HP1α (1:1000, Millipore, Cat# 05-689)
anti-αTubulin (1:5000, MBL, Cat# PM054)
anti-PrimPol (1:3000, Proteintech, Cat# 29824-1-AP)
anti-PrimPol (1:1000, Millipore, Cat# MABE2019)
anti-CCDC111 (10-B3) (1:2000, Invitrogen, Cat# MA5-32899)
anti-SMARCAL1 (1:1000, CST, Cat# 44717)
anti-SMARCAL1 (1:200, Santa Cruz, Cat# 376377)
anti-myc tag (1:500, MBL, Cat# 192-3)
anti-GST tag (1:1000, MBL, Cat# PM013)
anti-GAPDH (1:1000, Wako, Cat# 016-25523)
anti-Rev1 (1:100, Santa Cruz, Cat# sc-393022)
anti-Rev7 (1:1000, abcam, Cat# ab180579)
anti-Pol h(1:1000, CST, Cat# 13848)
anti-Pol i(1:1000, Proteintech, Cat# 13635-1-AP)
anti-Pol k(1:1000, Proteintech, Cat# 14455-1-AP)
anti-HLTF (1:1000, Proteintech, Cat# 14286-1-AP)
anti-phospho ATR Thr1989 (1:1000, CST, Cat# 30632)
anti-phospho ATM Ser1981 (1:1000, CST, Cat#5883)
anti-phospho DNAPKcs Ser2056 (1:1000, abcam, Cat# 124918)
anti-phospho Rad17 Ser645 (1:1000, Bethyl, Cat# A300-153A)
anti-phospho Chk1 Ser296 (1:1000, CST, Cat# 90178)
anti-phospho Chk1 Ser345 (1:1000, CST, Cat# 2348)
anti-phospho RPA32 Ser33 (1:1000, Bethyl, Cat# A300-246A)
anti-phospho PrimPol Ser255 (1:1000, custom antibody raised against LERLG[pS]AEQSSPDLS, Sigma-Aldrich)
anti-BrdU/IdU (1:25, BD Biosciences, Cat# 347580)
anti-BrdU/CldU (1:100, Abcam, Cat# ab6326)
anti-FLAG tag (MBL, Cat#M185-3L; IP)

Validation

All antibodies were validated by WB, IP, or IF. anti-pan-RAS was validated by expressing exogenous KRAS. anti-phospho POLII Ser2 was validated by DRB (CDK9i) treatment. anti-HP1α, SMARCAL1 (CST), Rev1, Rev7, Pol h, Pol i, Pol k, and HLTF were validated by silencing of the target proteins. PrimPol (Proteintech, Millipore) were validated by gene KO, KD and IP. SMARCAL1 (Santa Cruz) were validated by IP. anti-phospho ATR Thr1989, anti-phospho ATM Ser1981, anti-phospho DNAPKcs Ser2056, anti-phospho Rad17 Ser645, anti-phospho Chk1 Ser296/Ser345, anti-phospho RPA32 Ser33, anti-αTubulin, anti-GAPDH, myc-tag, GST-tag, and FLAG-tag have been used in several publications. House made anti-phospho PrimPol Ser255 were validated with in vitro Chk1 kinase assay. anti-BrdU are critical reagents widely used for DNA fiber assay.

Eukaryotic cell lines

Policy information about cell lines and Sex and Gender in Research

Cell line source(s)

Human: 293FT (Gift from Dr. Takata at Kyoto University, Japan)
Human: SAEC (Lonza, Cat# CC-2547, Gift from Dr. Kiyono at National Cancer Center, Japan)
Human: RPE-1 (Gift from Dr. Hochegger at University of Sussex, UK)
Human: H322 (ATCC, Cat# CRL-5806, RRID:CVCL_1556, *discontinued*)
Human: H1819 (ATCC, Cat# CRL-5897, RRID: CVCL_1497)
Human: H358 (ATCC, Cat# CRL-5807, RRID: CVCL_1559)
Human: H2009 (ATCC, Cat# CRL-5911, RRID: CVCL_1514)

Authentication

N/A

Mycoplasma contamination

All cell lines are free of Mycoplasma contamination. Cells were periodically treated with Plasmocin (InvivoGen) and replaced by younger passage.

Commonly misidentified lines
(See ICLAC register)

N/A

Plants

Seed stocks: N/A

Novel plant genotypes: N/A

Authentication: N/A

Flow Cytometry

Plots

Confirm that:

☒ The axis labels state the marker and fluorochrome used (e.g. CD4-FITC).

☒ The axis scales are clearly visible. Include numbers along axes only for bottom left plot of group (a 'group' is an analysis of identical markers).

☒ All plots are contour plots with outliers or pseudocolor plots.

☒ A numerical value for number of cells or percentage (with statistics) is provided.

Methodology

Sample preparation: A total of 8 × 10^5 cells were collected following trypsinization and washed once with PBS. Cells were resuspended in 100 µL of PBS containing 0.1% Triton X-100 and 300 µg/mL RNase A, and incubated for 10 min at room temperature. After centrifugation and a PBS wash, cells were resuspended in 100 µL of PBS containing 10 µg/mL propidium iodide (PI) and incubated for 10 min at room temperature. Cells were washed again with PBS and finally resuspended in 1 mL PBS for analysis.

Instrument: BD FACSCanto II (BD Bio)

Software: BD FACSDiva software (BD Bio)

Cell population abundance: No sorting experiments is provided; FCM for cell cycle analysis.

Gating strategy: Typical flow cytometry gating was applied to exclude obvious debris.

☒ Tick this box to confirm that a figure exemplifying the gating strategy is provided in the Supplementary Information.
