# Supplementary material for: PrimPol toggled by ATR modulates fork slowing and repriming under oncogenic KRAS-induced replication stress
Source: Nat Commun. 2026 Oct 1;17:11277. doi: 10.1038/s41467-026-77585-y (PMC13720195; doi:10.1038/s41467-026-77585-y)
Supplement: Supplementary file 3 [file 41467_2026_77585_MOESM3_ESM.pdf]

nature portfolio

Peer Review File

PrimPol Toggled by ATR Modulates Fork Slowing and Repriming under Oncogenic KRAS-induced Replication Stress

Corresponding Author: Dr Bunsyo Shiotani

This file contains all reviewer reports in order by version, followed by all author rebuttals in order by version.

Version 0:

Reviewer comments:

Reviewer #1

(Remarks to the Author)
In this manuscript Igarashi et al. expand their previous work on the control of replication stress during malignant transformation induced by constitutively active KRAS. The authors dissect the effects of acute activation of KRAS and compare it to models where KRAS activation is chronic. The replication stress response (RSR) is a barrier for transformation and, at the same time, transformed cells adapt to growing in the presence of increased levels of replication stress. How specific oncogenes activate and modulate the RSR to enable the growth of cancer cells has not been clarified yet. Since the RSR is an emerging target in cancer treatment, the manuscript addresses a very relevant mechanistic question with broad implications in cancer therapy. In particular, KRAS driven tumors are highly prevalent and particularly difficult to treat, making this work interesting to a wide audience.
The authors employ an in vitro model of KRAS induced transformation in airway epithelial cells to study the effects of the acute activation of KRAS, as well as other complementary systems of transformed and non-transformed cells. For the chronic effect of active KRAS the authors use lung cancer cell lines with or without KRAS mutations. They conclude that PRIMPOL is central to deal with replication stress induced by KRAS, as it slows down replication forks in response to the acute activation of KRAS, and promotes re-priming in the presence of chronic activation of KRAS. They propose that the phosphorylation of PRIMPOL and SMARCAL1 by the RSR regulates their interaction, favoring fork slowing or re-priming. The manuscript is clearly written, the results are well presented and the main conclusions are supported by the data. The use of different models confers strength to many of the results but, at the same time, it makes it difficult to connect the results from acute and chronic activation of KRAS. The basis for the proposed model is solid, but additional mechanistic evidence for the interplay between PRIMPOL, SMARCAL1 and the RSR is necessary to offer support for the model. I suggest the authors address the following points:

1. There are no direct comparisons of the different models used regarding the levels of the key components of the pathway. A comparative analysis of the changes of PRIMPOL, SMARCAL1, the RSR and markers of RSR activation is necessary to show how the acute versus chronic effects of active KRAS work. On one hand, to understand the effect of acute expression of active KRAS in SAEC. On the other hand, to understand how the cancer cell lines with active KRAS compare to the cells without KRAS mutation and with the SAEC model. The proposed model implies higher activation of the RSR and a shift in the functions of PRIMPOL-SMARCAL1. This change could be influenced by changes in expression and activation of the three components.
2. Along these lines, the model proposes that PRIMPOL and SMARCAL1 phosphorylation by CHK1 and ATR control their interaction and function. However, their phosphorylation status is not addressed throughout the paper. The use of PRIMPOL-S255A mutant provides some support to the data but it is not enough to actually show that the phosphorylation itself is relevant. In addition, the rescue experiments with PRIMPOL-S255A lead to a very strong overexpression of the protein, which could be leading to indirect effects. Can the authors achieve a more modest expression, similar to the wild type? A similar rescue approach could be explored with SMARCAL1 mutants.
3. Alternatively, the authors could directly analyze the phosphorylation of both proteins and their interaction. I understand that the detection of phosphorylated PRIMPOL and SMARCAL1 may be challenging. Immunoprecipitation and Western blot or mass spectrometry analysis could be carried out in case the available antibodies are not good enough for a direct analysis by Western blot. This is particularly relevant to show how ATR and CHK1 inhibitors differentially affect the pathway. The interaction of PRIMPOL and SMARCAL1 is only assayed once in the context of rescue with the PRIMPOL-S255A

mutant. Co-immunoprecipitation should be confirmed with endogenous proteins and in different models, such as the KRAS wild type and mutant cancer cell lines. Showing that the interaction and phosphorylation of these proteins is actually regulated would further substantiate the proposed model.
4. The authors could also evaluate the presence of PRIMPOL and SMARCAL1 in replication forks using iPOND or SIRF assays. These experiments would support a direct effect on the control of DNA replication.
5. The previous work of the authors showed that mutant KRAS expression leads to the generation of transformed cells. However, they do not analyze these cells as a model of chronic KRAS activation. Is the phenotype in the KRAS SAEC cells similar to lung cancer cell lines with active KRAS?
6. It is not clear how many times the authors repeated the DNA fiber assays. Most of the experiments show consistent reproducibility throughout different experiments. However, the statistical analysis should consider each independent experiment as replicate, not each fiber that has been measured.
7. The analysis of ssDNA accumulation with the S1 DNA fiber assay in Figure 3A is missing the control of S1 digestion in control SAEC without mutant KRAS expression, to confirm that the cells do not present high levels of ssDNA.
8. No cell cycle analysis is performed with the different treatments. In cases as the treatment with ATR and CHK1 inhibitors for 1 day, they can lead to a significant change in the number of cells in S phase that could influence the interpretation of the data.

Reviewer #2

(Remarks to the Author)
I co-reviewed this manuscript with one of the reviewers who provided the listed reports. This is part of the Nature Communications initiative to facilitate training in peer review and to provide appropriate recognition for Early Career Researchers who co-review manuscripts.

Reviewer #3

(Remarks to the Author)
In the study, the authors reported a novel function of PrimPol in regulating acute and persistent heterochromatin-associated replication stress induced by KRAS oncogene activation (K-HaRS). Mechanistically, phosphorylation of PrimPol by ATR signaling provides a molecular switch to coordinate its functions in slowing fork progression through SMARCAL1 regulation in the presence of acute K-HaRS, and in enabling repriming and replication progress across the heterochromatin barrier in the presence of persistent K-HaRS. Furthermore, loss of PrimPol renders a dependence on the error-prone Rev1-Pol -dependent synthesis. These mechanistic findings reveal how PrimPol regulates replication stress tolerance (RST) in two different modes in different K-HaRS contexts (acute vs persistent) via ATR-mediated phosphorylation. In addition, the analysis of TCGA lung cancer datasets further highlights the clinical relevance of these two modes in tumor samples. Overall, this study utilized genetic, cell biology, and molecular biology approaches to dissect the mechanistic details of how PrimPol differentially coordinates cellular responses to K-HaRS at the acute stage and at the chronic stage. The experiments were well-designed and executed. Results from this study are expected to bring new understanding of how cells cope with replication stress, gain an optimal compromise of genome stability, and maintain survival fitness under oncogenic activation. There are some concerns regarding the experiment design and data interpretation.

1. In Figure 1G, acute KRAS activation reduces cell proliferation. Does PrimPolS255A sufficiently restore cell proliferation in cells with acute KRAS-induced replication stress, or does it not? Conversely, does PrimPolS255A not sufficiently restore cell proliferation in cells with chronic KRAS-induced replication stress? It remains to be determined whether the function of PrimPolS255A mutant in regulating fork slowing but not repriming is sufficient to rescue the phenotype of cell proliferation in the presence of acute but not in the presence of chronic KRAS-activation.

2. K-RAS induction can induce acute and persistent activation of DNA damage signaling pathways, such as ATR/ATM, as evidenced by previous studies. The project's premise is that acute K-RAS activation leads to basal ATR activity, while chronic K-RAS leads to high ATR activity. However, no experimental evidence is presented to demonstrate the extent of ATR activation at basal levels (phosphorylated or its downstream kinase substrates) in the cell models tested when acute K-RAS is present. It's intriguing that PrimPol S255 phosphorylation is restricted or prevented during the acute stage to allow its function in its unphosphorylated form if ATR signaling is present at low levels. Does the phosphorylated form of PrimPol abolish its binding to SMARCAL1?

3. The study employed PrimPol phosphor-mutant as a genetic approach to elucidate the function of phosphorylation. Furthermore, in Fig. 4, a very low concentration of ATR inhibitor (1nM) was used to demonstrate the necessity of ATR in PrimPol-dependent repression in response to chronic K-HaRS. However, it remains uncertain whether this low level of ATR inhibitor treatment effectively abolished ATR activation and PrimPol phosphorylation. Additionally, considering the cross-activation of phosphorylation substrates by ATM, ATR, and DNAPK, it is unclear whether ATR inhibition alone is sufficient to eliminate PrimPol phosphorylation. Therefore, control experiments are essential to address these concerns.

4. P53 deactivation frequently occurs in tumor cells as an escape mechanism from DNA damage checkpoint activation triggered by K-RAS activation. In animal models of various tumor types, loss of p53 is commonly used to facilitate tumor development in the presence of Kras mutations. If p53 is inactivated, does PrimPol play a role in slowing or repressing fork progression or cell proliferation in the presence of acute or chronic K-RAS? Does the loss of p53 override the requirement of

PrimPol during the oncogenic transformation? Furthermore, how do these two distinct groups correlate with p53 mutation status, as per the TCGA analysis?

5. In addition, KRAS mutations are almost found in all pancreatic cancer tumors. Can the conclusions drawn from two modes be validated using pancreatic tumor TCGA samples, or are these observations highly specific to lung cancer models?

Reviewer #4

(Remarks to the Author)
This study has identified two distinct roles of PrimPol in replication in response to KRAS-induced replication stress. In an acute KRAS-induced heterochromatin-associated replication stress (K-HaRS), PrimPol functions with SMARCAL1 to slow down the replication fork progression suppressing Rev1 and polymerase zeta-dependent translesion DNA synthesis (TLS). In KRAS-driven tumor cells, however, ATR-dependent PrimPol repriming function is required to maintain replication fork speed. The authors therefore propose two distinct PrimPol-involved replication stress tolerance mechanism which is relying on the ATR activation mode in response to acute and chronic H-HaRS, respectively. While the role of PrimPol in acute KRAS-induced replication stress is clear and convincing, the mechanism in the chronic KRAS-induced response needs to be further strengthened. Overall, the study presents an interesting model for us to overview the dynamic regulation of replication stress response mechanism and genome instability during KRAS-induced tumor progression. However, there are two major concerns that need to be clarified.

The chronic H-HaRS is not well defined. The authors appear to equal two KRAS-mutant cancer cells (mostly just H358 cells) to cells with chronic H-HaRS and argue that these cells possess high ATR activity which leads to PrimPol Ser 255 phosphorylation and suppression of SMARCAL1 to favor PrimPol-dependent repriming. Additional experiments and controls need to be included for the status of ATR, Chk1, PrimPol Ser255 phosphorylation, SMARCAL1, etc.

It is not clear whether only unphosphorylated PrimPol interacts with SMARCAL1. The authors show that S255A mutant of PrimPol interacts with SMARCAL1 and argue that this underlies the choice of fork slowing when ATR activity is low. However, it’s not clear whether ATR activity regulates the interaction of PrimPol with SMARCAL1.

Fig. 2g, does PrimPol WT interact with SMARCAL1? Is the interaction subject to the regulation of ATR?

Fig. 2f, 5b, does PrimPol WT behave similarly as PrimPol S255A?

Fig.5 and S5, it lacks comparison between Kras wt and Kras mt tumor for the status of ATR, PrimPol and SMARCAL1?

Version 1:

Reviewer comments:

Reviewer #1

(Remarks to the Author)
In the revised version of the manuscript, the authors have answered most of the questions raised by us in the original revision. The comparison of the different cellular models and the in-depth analysis of the interaction of PRIMPOL with SMARCAL1, as well as PRIMPOL phosphorylation, provides a very solid model to describe how the RSR changes from acute to chronic activation. The work is of high interest to the field and very high quality, and we support its publication in Nature Communications.

Reviewer #2

(Remarks to the Author)
I co-reviewed this manuscript with one of the reviewers who provided the listed reports. This is part of the Nature Communications initiative to facilitate training in peer review and to provide appropriate recognition for Early Career Researchers who co-review manuscripts.

Reviewer #3

(Remarks to the Author)
The authors have adequately addressed my concerns. The additional experimental data and discussions provided in the revised manuscript are satisfactory, and I have no further questions.

Reviewer #4

(Remarks to the Author)
The authors have addressed all my concerns.

Open Access This Peer Review File is licensed under a Creative Commons Attribution 4.0 International License, which permits use, sharing, adaptation, distribution and reproduction in any medium or format, as long as you give appropriate credit to the original author(s) and the source, provide a link to the Creative Commons license, and indicate if changes were made.
In cases where reviewers are anonymous, credit should be given to 'Anonymous Referee' and the source.
The images or other third party material in this Peer Review File are included in the article's Creative Commons license, unless indicated otherwise in a credit line to the material. If material is not included in the article's Creative Commons license and your intended use is not permitted by statutory regulation or exceeds the permitted use, you will need to obtain permission directly from the copyright holder.
To view a copy of this license, visit https://creativecommons.org/licenses/by/4.0/

REVIEWER COMMENTS

Reviewer #1 (Remarks to the Author):

In this manuscript Igarashi et al. expand their previous work on the control of replication stress during malignant transformation induced by constitutively active KRAS. The authors dissect the effects of acute activation of KRAS and compare it to models where KRAS activation is chronic. The replication stress response (RSR) is a barrier for transformation and, at the same time, transformed cells adapt to growing in the presence of increased levels of replication stress. How specific oncogenes activate and modulate the RSR to enable the growth of cancer cells has not been clarified yet. Since the RSR is an emerging target in cancer treatment, the manuscript addresses a very relevant mechanistic question with broad implications in cancer therapy. In particular, KRAS driven tumors are highly prevalent and particularly difficult to treat, making this work interesting to a wide audience.
The authors employ an in vitro model of KRAS induced transformation in airway epithelial cells to study the effects of the acute activation of KRAS, as well as other complementary systems of transformed and non-transformed cells. For the chronic effect of active KRAS the authors use lung cancer cell lines with or without KRAS mutations. They conclude that PRIMPOL is central to deal with replication stress induced by KRAS, as it slows down replication forks in response to the acute activation of KRAS, and promotes re-priming in the presence of chronic activation of KRAS. They propose that the phosphorylation of PRIMPOL and SMARCAL1 by the RSR regulates their interaction, favoring fork slowing or re-priming. The manuscript is clearly written, the results are well presented and the main conclusions are supported by the data. The use of different models confers strength to many of the results but, at the same time, it makes it difficult to connect the results from acute and chronic activation of KRAS. The basis for the proposed model is solid, but additional mechanistic evidence for the interplay between PRIMPOL, SMARCAL1 and the RSR is necessary to offer support for the model. I suggest the authors address the following points:

Thank you for your understanding and comments. As shown below, we conducted experiments to support our conclusions. We believe that all of your concerns are addressed by the revision.

1. There are no direct comparisons of the different models used regarding the levels of the key components of the pathway. A comparative analysis of the changes of PRIMPOL, SMARCAL1, the RSR and markers of RSR activation is necessary to show how the acute versus chronic effects of active KRAS work. On one hand, to understand the effect of acute expression of active KRAS in SAEC. On the other hand, to understand how the cancer cell lines with active KRAS compare to the cells without KRAS mutation and with the SAEC model. The proposed model implies higher activation of the RSR and a shift in the functions of PRIMPOL-SMARCAL1. This change could be influenced by changes in expression and activation of the three components.

We thank the reviewer for this important comment. To address this point, we have now added a comparative analysis of total PrimPol, SMARCAL1, and multiple RSR activation markers in $SAEC^{KRAS-}$ cells, $SAEC^{KRAS+}$ cells undergoing acute KRAS-induced heterochromatin-associated replication stress (K-HaRS), SAEC-derived RSTCs that have adapted to persistent K-HaRS, and KRAS-driven lung cancer cell lines representing adapted/chronic K-HaRS states, together with non-KRAS-driven lung cancer controls. (Revised Fig. 5a)
In these experiments, total PrimPol and SMARCAL1 levels were broadly comparable across the models, although modest cell-line-dependent differences were observed. Thus, the functional transition proposed in our model is unlikely to be explained simply by changes in PrimPol or SMARCAL1 abundance. In contrast, RSR activation markers showed model-dependent changes. In particular, Rad17 Ser645 phosphorylation was barely detectable in $SAEC^{KRAS-}$ cells, but increased upon acute KRAS activation and in RSTCs, indicating that ATR/RSR signaling is already induced during the acute phase of KRAS-driven replication stress. Rad17 phosphorylation, together with Chk1 Ser296/Ser345 phosphorylation, was also detected in lung cancer cell lines irrespective of KRAS mutation status, consistent with broader endogenous RSR activation in established cancer cells. Notably, ATR Thr1989 phosphorylation was more prominently detected in RSTCs and the KRAS-driven H358 and H2009 cells, which exhibit PrimPol-mediated repriming activity. However, some lung cancer cell lines showed strong RAD17 phosphorylation despite relatively weak ATR Thr1989 phosphorylation. This distinction further supports the idea that global ATR/RSR activation is associated with, but does not fully define, the PrimPol-dependent repriming state.
We therefore further examined PrimPol Ser255 phosphorylation as a potential marker of repriming-active state. We generated a phospho-specific antibody against PrimPol Ser255 and validated its specificity using biochemical assays. In a Phos-tag assay using purified full-length PrimPol as a substrate and Chk1 as a kinase, we observed an ATP-dependent mobility shift, which was abolished by Chk1 inhibition (Revised Supplementary Fig. 4l). In addition, using $GST\text{-}PrimPol^{250\text{-}260}$, which contains a short peptide from PrimPol surrounding Ser255,

an ATP-dependent signal was detected with the wild-type peptide but not with the S255A mutant peptide (Revised Supplementary Fig. 4m). These results indicate that our phospho-PrimPol Ser255 antibody specifically recognizes PrimPol phosphorylated at Ser255 under defined biochemical conditions.

Because endogenous PrimPol Ser255 phosphorylation was difficult to detect reliably by conventional immunoblotting, likely due to its transient and low-stoichiometry nature, we applied this antibody to a PLA-based assay using mouse anti-PrimPol and our rabbit anti-phospho-PrimPol Ser255 antibodies in cells. PrimPol Ser255 phosphorylation-associated PLA signals were readily detected in the KRAS-driven lung cancer cell lines H358 (Revised Fig. 4k) and H2009 (Revised Supplementary Fig. 4n), and these signals were reduced by ATR inhibition and PrimPol depletion. In contrast, only limited PLA signals were detected in $SAEC^{KRAS-}$ cells and in the non-KRAS-driven lung cancer cell lines H322 and H1819 (Revised Supplementary Fig. 4o). Thus, PrimPol Ser255 phosphorylation-associated PLA signals are preferentially enriched in KRAS-driven, repriming-active cells rather than simply reflecting global ATR/RSR activation.

Together, these new data refine our model. Acute KRAS activation induces detectable ATR/RSR signaling, as indicated by RAD17 phosphorylation (Revised Fig. 5a), whereas PrimPol Ser255 phosphorylation-associated PLA signals were preferentially associated with KRAS-driven repriming-active states (Revised Fig. 4k and Revised Supplementary Fig. 4n) and do not simply mirror global ATR/RSR activation. We have revised the manuscript accordingly.

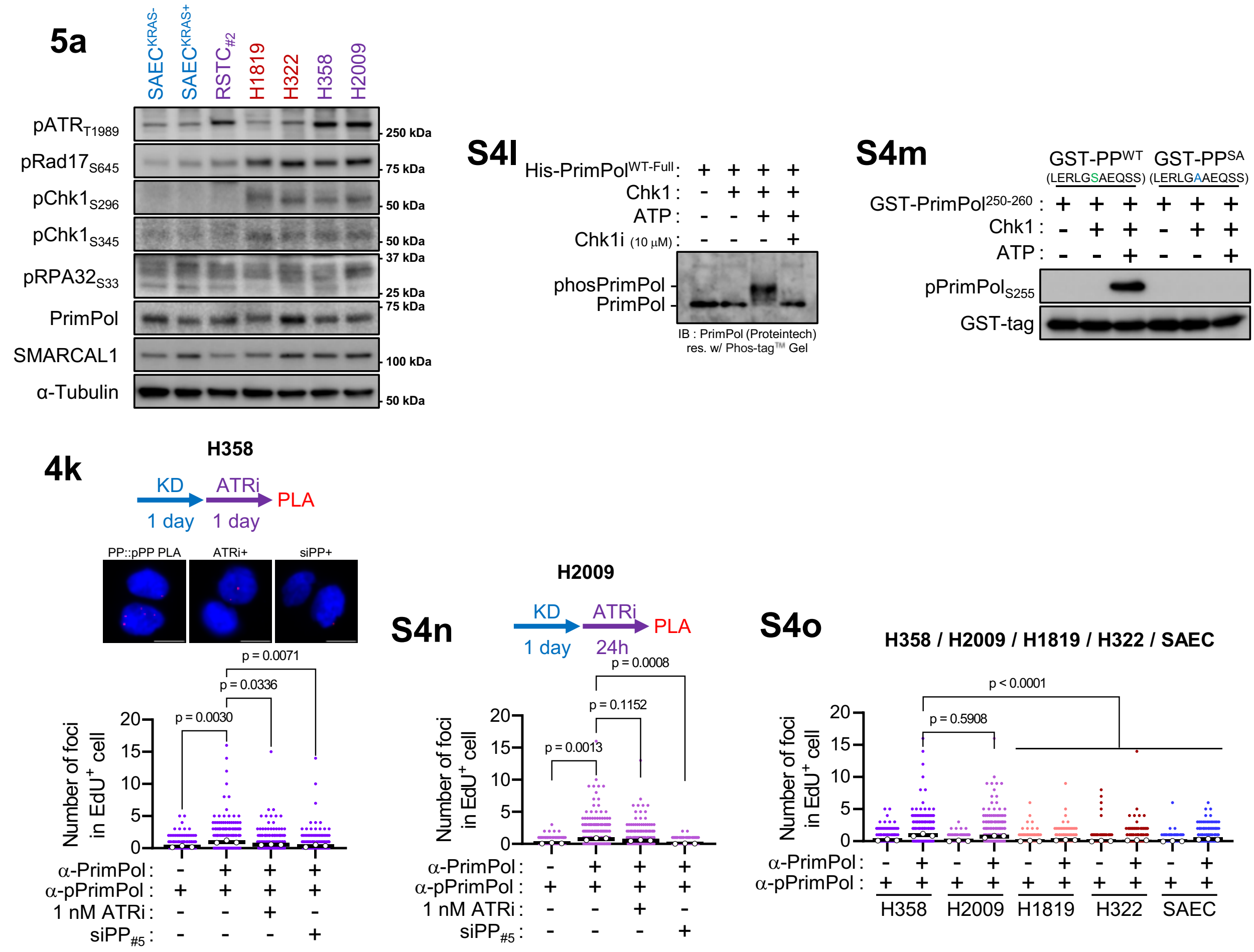

2. Along these lines, the model proposes that PRIMPOL and SMARCAL1 phosphorylation by CHK1 and ATR control their interaction and function. However, their phosphorylation status is not addressed throughout the paper. The use of PRIMPOL-S255A mutant provides some support to the data but it is not enough to actually show that the phosphorylation itself is relevant. In addition, the rescue experiments with PRIMPOL-S255A lead to a very strong overexpression of the protein, which could be leading to indirect effects. Can the authors achieve a more modest expression, similar to the wild type? A similar rescue approach could be explored with SMARCAL1 mutants.

Thank you for raising these important points. As described in our response to Comment 1, we have now added biochemical validation of our newly generated phospho-Ser255 PrimPol antibody using Phos-tag and peptide kinase assays, together with PLA-based detection of PrimPol Ser255 phosphorylation-associated signals in

cells (Revised Fig. 4k and Revised Supplementary Fig. 4l, m, n, o). These data support the specificity of the antibody and provide additional evidence that PrimPol Ser255 phosphorylation is linked to ATR-Chk1 pathway activity and preferentially associated with the repriming-active state.

To directly address the reviewer's concern regarding PrimPol overexpression, we optimized the Dox concentration and repeated the rescue experiment under conditions in which PrimPol^{S255A} was expressed at a much more modest level, close to the endogenous PrimPol levels (Revised Fig. 2e, and 5d). Even under these reduced-expression conditions, PrimPol^{S255A} rescued the active fork slowing defect caused by PrimPol depletion in SAECs undergoing acute K-HaRS (Revised Fig. 2f), and supported active fork slowing in ATR-inhibited H358 cells (Revised Fig. 5e). These results argue against the possibility that the phenotype observed with PrimPol^{S255A} was simply caused by excessive overexpression, and support the functional relevance of the PrimPol Ser255 phosphorylation state.

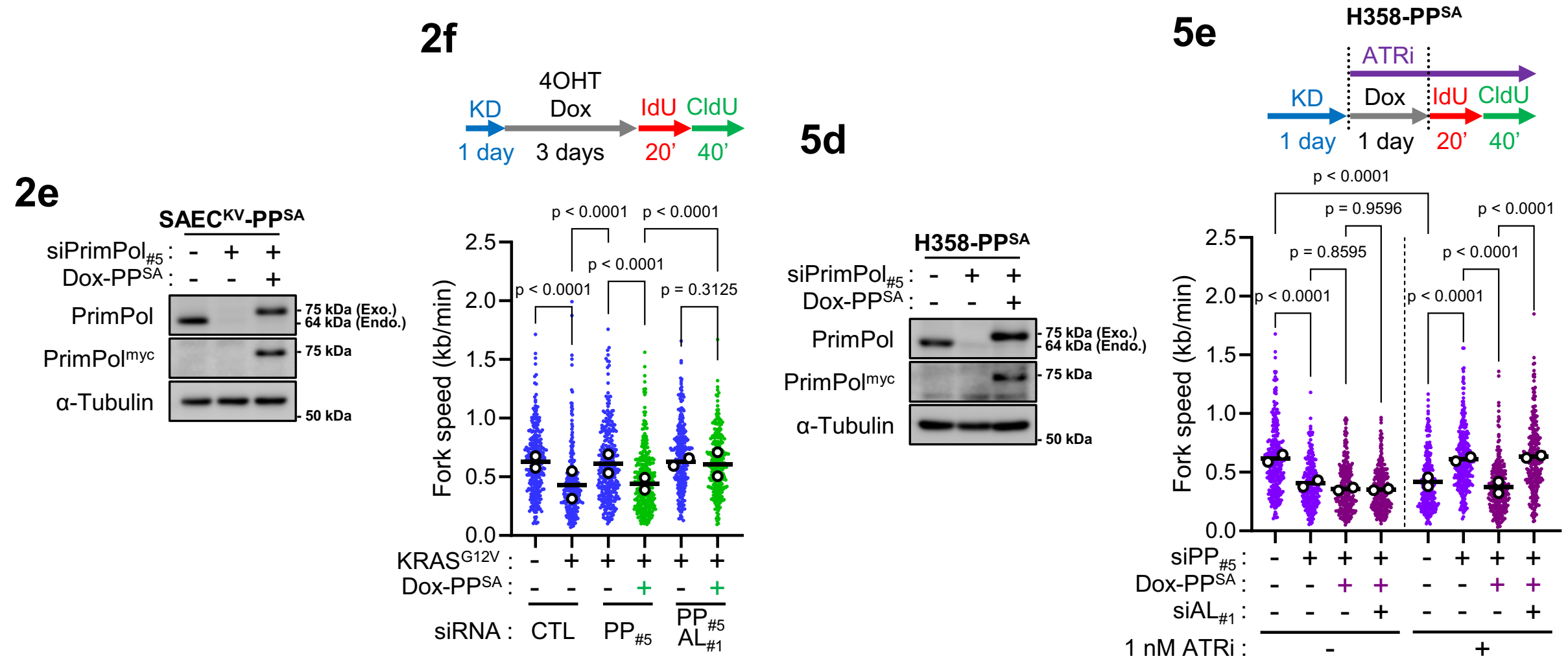

We also added co-immunoprecipitation analyses to test whether the PrimPol Ser255 phosphorylation state affects PrimPol-SMARCAL1 association. In a 293FT transient expression system, Flag-tagged PrimPol co-immunoprecipitated with SMARCAL1. This interaction was enhanced rather than reduced by Benzonase or RNase A treatment, suggesting that the detected interaction is unlikely to be mediated by DNA or RNA (Revised Fig. 2h). Importantly, the non-phosphorylatable PrimPol^{S255A} mutant associated with SMARCAL1 more efficiently than PrimPol^{WT} and the phosphomimetic PrimPol^{S255D} (Revised Fig. 2j). These results are consistent with a model in which the non-phosphorylated state of PrimPol favors association with SMARCAL1 to support active fork slowing.

Regarding SMARCAL1 mutants, we agree that a similar rescue approach would be informative. We have generated SMARCAL1 Ser652 mutants, focusing on a phosphorylation site previously implicated in SMARCAL1 regulation, to begin addressing how this site affects the PrimPol-SMARCAL1 interaction. Because a systematic functional analysis of SMARCAL1 phosphorylation would require additional mapping and validation, we have focused the current revision on the experimentally supported PrimPol Ser255-dependent mechanism, while discussing SMARCAL1 phosphorylation as a potential additional regulatory mechanism.

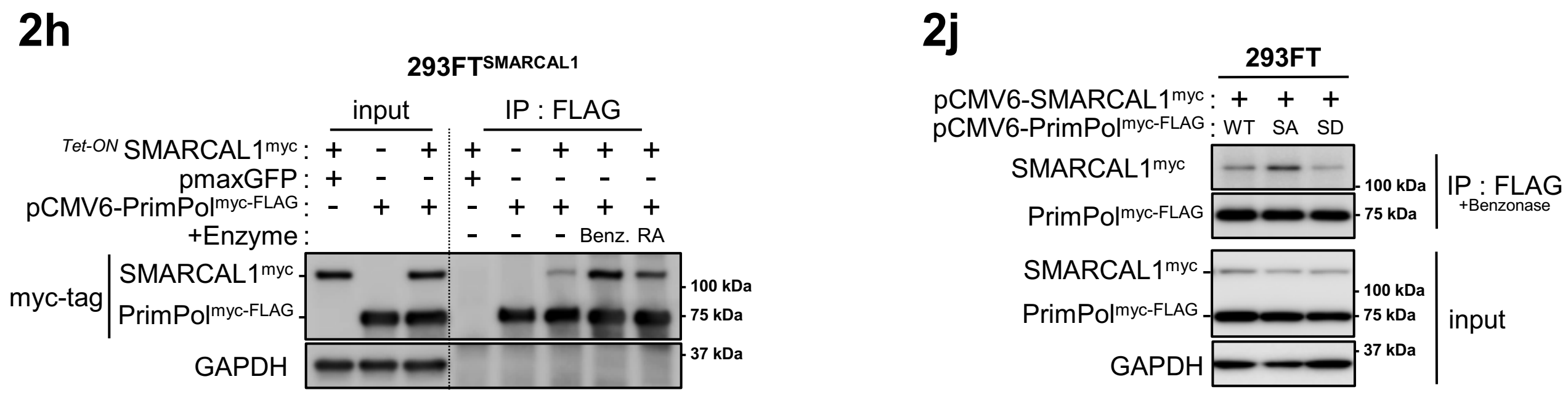

3. Alternatively, the authors could directly analyze the phosphorylation of both proteins and their interaction. I understand that the detection of phosphorylated PRIMPOL and SMARCAL1 may be challenging. Immunoprecipitation and Western blot or mass spectrometry analysis could be carried out in case the available antibodies are not good enough for a direct analysis by Western blot. This is particularly relevant to show how ATR and CHK1 inhibitors differentially affect the pathway. The interaction of PRIMPOL and SMARCAL1 is only

assayed once in the context of rescue with the PRIMPOL-S255A mutant. Co-immunoprecipitation should be confirmed with endogenous proteins and in different models, such as the KRAS wild type and mutant cancer cell lines. Showing that the interaction and phosphorylation of these proteins is actually regulated would further substantiate the proposed model.

As described in our response to Comment 1, we have now added biochemical validation of our phospho-Ser255 PrimPol antibody, as well as PLA-based detection of PrimPol Ser255 phosphorylation-associated signals in cells (Revised Fig. 4k and Revised Supplementary Fig. 4l-o). These experiments address the PrimPol phosphorylation aspect of this comment and show that PrimPol Ser255 phosphorylation is linked to ATR-Chk1 pathway activity and preferentially associated with the repriming-active state.
We also agree that the PrimPol-SMARCAL1 interaction should be validated beyond the original experiment with the $PRIMPOL^{S255A}$ mutant. As described in our response to Comment 2, we therefore added co-immunoprecipitation analyses in a 293FT expression system, showing that the non-phosphorylatable $PrimPol^{S255A}$ mutant associated with SMARCAL1 more efficiently than $PrimPol^{WT}$ and the phosphomimetic $PrimPol^{S255D}$ (Revised Fig. 2j). These results support the idea that the PrimPol Ser255 phosphorylation state modulates PrimPol-SMARCAL1 association.
To further confirm this interaction at the endogenous level, we performed endogenous SMARCAL1 immunoprecipitation in SAECs and H358 cells. Endogenous PrimPol was co-immunoprecipitated with SMARCAL1 in SAECs undergoing acute K-HaRS (Revised Fig 2i), a condition in which active fork slowing is engaged, whereas this interaction was barely detectable in H358 cells under basal conditions (Revised Fig. 5b), suggesting that the PrimPol-SMARCAL1-associated fork-slowing configuration is available in normal epithelial cells but is suppressed or disengaged in repriming-dominant KRAS-driven cancer cells. Notably, ATR inhibition restored detectable PrimPol-SMARCAL1 association in H358 cells (Revised Fig. 5c). This finding is consistent with the functional re-engagement of SMARCAL1-dependent active fork slowing when ATR-dependent repriming is suppressed (Revised Fig. 4i). Together with the enrichment of PrimPol Ser255 phosphorylation-associated PLA signals in repriming-active H358/H2009 cells (Revised Fig 4k and Revised Supplementary Fig 4n), these data support a model in which the non-phosphorylated PrimPol state favors SMARCAL1 association, while Ser255 phosphorylation is associated with the transition toward the repriming-active state.

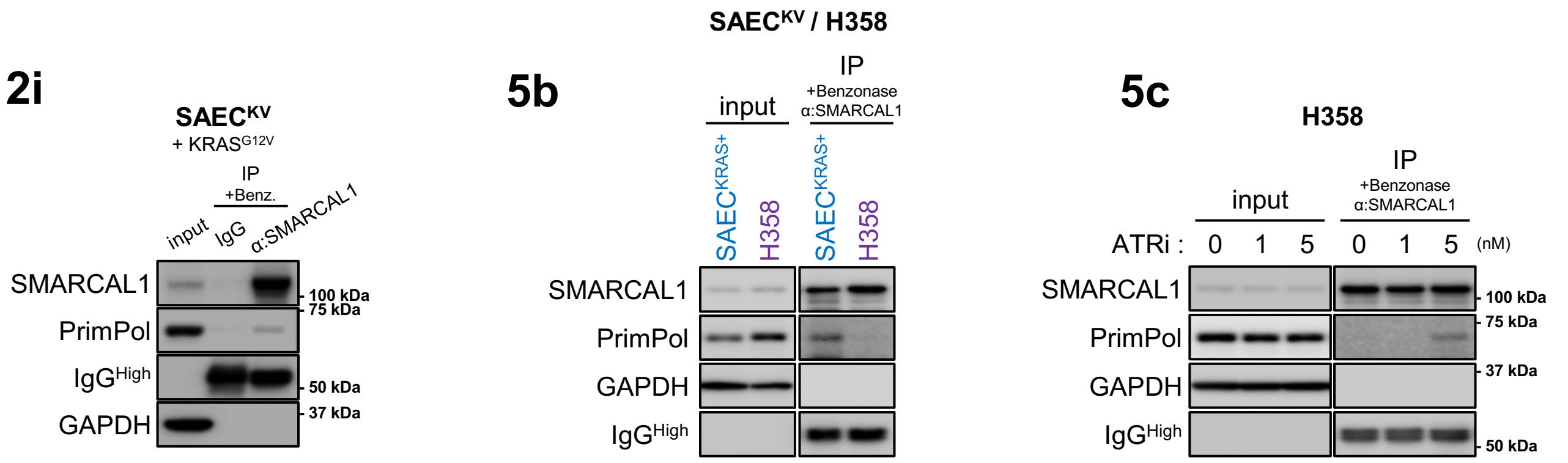

4. The authors could also evaluate the presence of PRIMPOL and SMARCAL1 in replication forks using iPOND or SIRF assays. These experiments would support a direct effect on the control of DNA replication.

We agree that iPOND or SIRF analysis of PrimPol and SMARCAL1 at nascent DNA would provide valuable spatial information and further support a direct role in DNA replication control. In the revised manuscript, we have strengthened the mechanistic evidence by adding endogenous PrimPol–SMARCAL1 co-immunoprecipitation and PLA-based detection of PrimPol Ser255 phosphorylation-associated signals. Nevertheless, iPOND or SIRF analysis require careful optimization and careful interpretation in the context of replication fork remodeling. Proximity to nascent DNA does not necessarily correlate with functional involvement in active fork slowing. For example, Lamin A/C is required for active fork slowing upon mild replication stress, yet Lamin A/C-EdU PLA signals decrease under the same conditions, suggesting altered spatial arrangement or dynamic interaction with nascent DNA during fork remodeling (PMID: 41318603). Similarly, MCM7-EdU PLA signals decrease during SMARCAL1/RAD51-dependent fork reversal despite retention of the CMG helicase at stalled forks (PMID: 37104614). We therefore consider that a detailed iPOND/SIRF-based analysis of PrimPol and SMARCAL1 dynamics at remodeled fork to require a dedicated study and to be more appropriate for future work.

5. The previous work of the authors showed that mutant KRAS expression leads to the generation of

transformed cells. However, they do not analyze these cells as a model of chronic KRAS activation. Is the phenotype in the KRAS SAEC cells similar to lung cancer cell lines with active KRAS?

In our previous study, we established replication stress-tolerant cells (RSTCs) from SAECs after prolonged $KRAS^{G12V}$ expression and showed that RSTCs maintain fork progression through PrimPol-dependent repriming in an ATR-Chk1-dependent manner (PMID: 37591859). Thus, RSTCs represent a SAEC-derived adapted K-HaRS model that arose after cells passed through the acute KRAS-induced replication stress state, enabling us to compare normal replication, active fork slowing, and PrimPol-dependent repriming within the same cellular lineage.

In the revised manuscript, we now clarify this point and include additional characterization of RSTCs. We show representative cell images to illustrate the distinct transformed morphology of RSTCs compared with parental SAECs (Revised Fig. 4e), and provide new data showing that RSTCs retain K-HaRS-associated features (Revised Fig. 4f, g). As described in response to Comment 1, we also added comparative immunoblot analyses of total PrimPol, SMARCAL1, and RSR activation markers across $SAEC^{KRAS-}$ cells, acute $SAEC^{KRAS+}$ cells, RSTCs, and lung cancer cell lines with or without KRAS mutations (Revised Fig. 5a). These analyses indicate that RSTCs share key replication stress-related features with KRAS-driven lung cancer cell lines, including RSR activation and reliance on PrimPol-mediated repriming, as previously characterized in detail (PMID: 37591859).

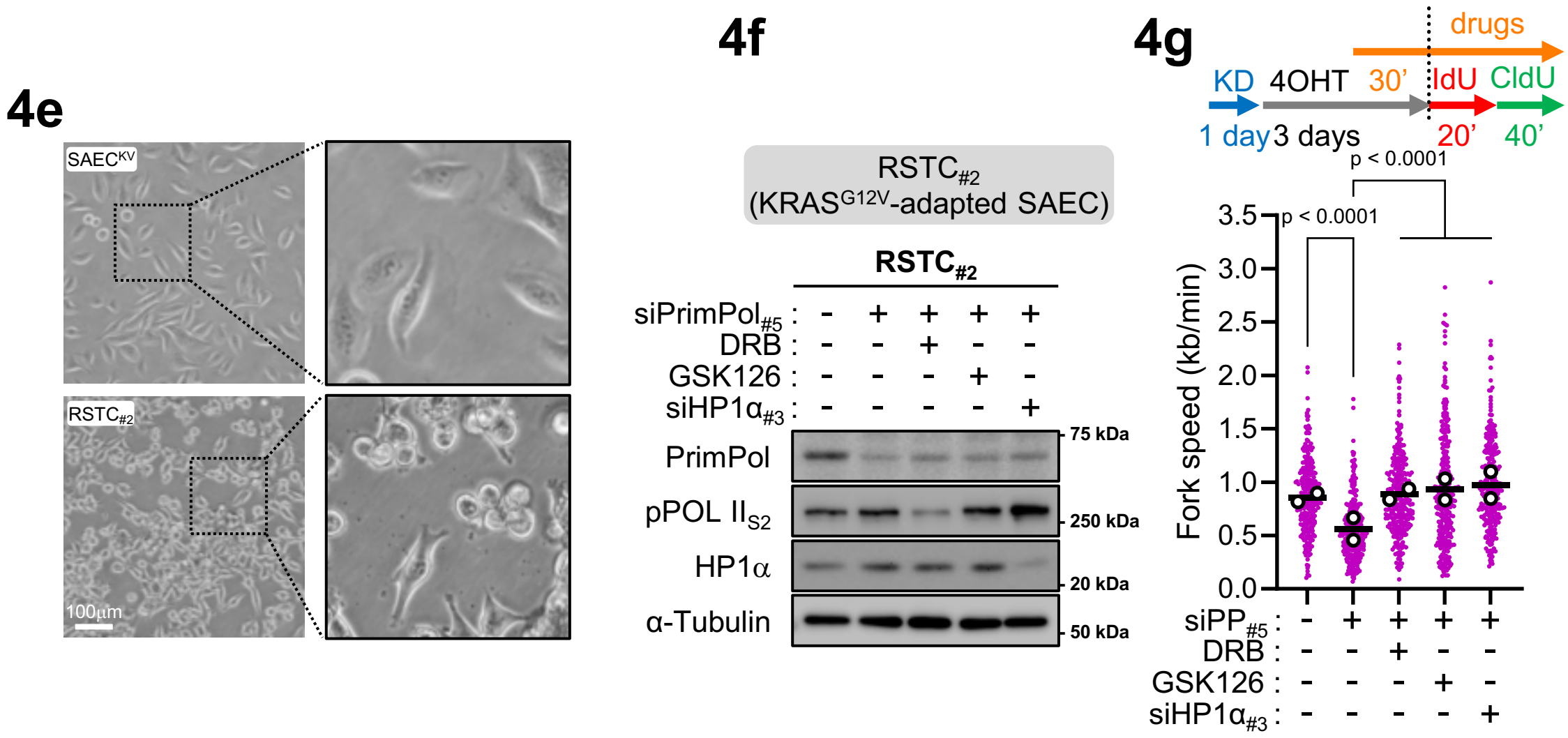

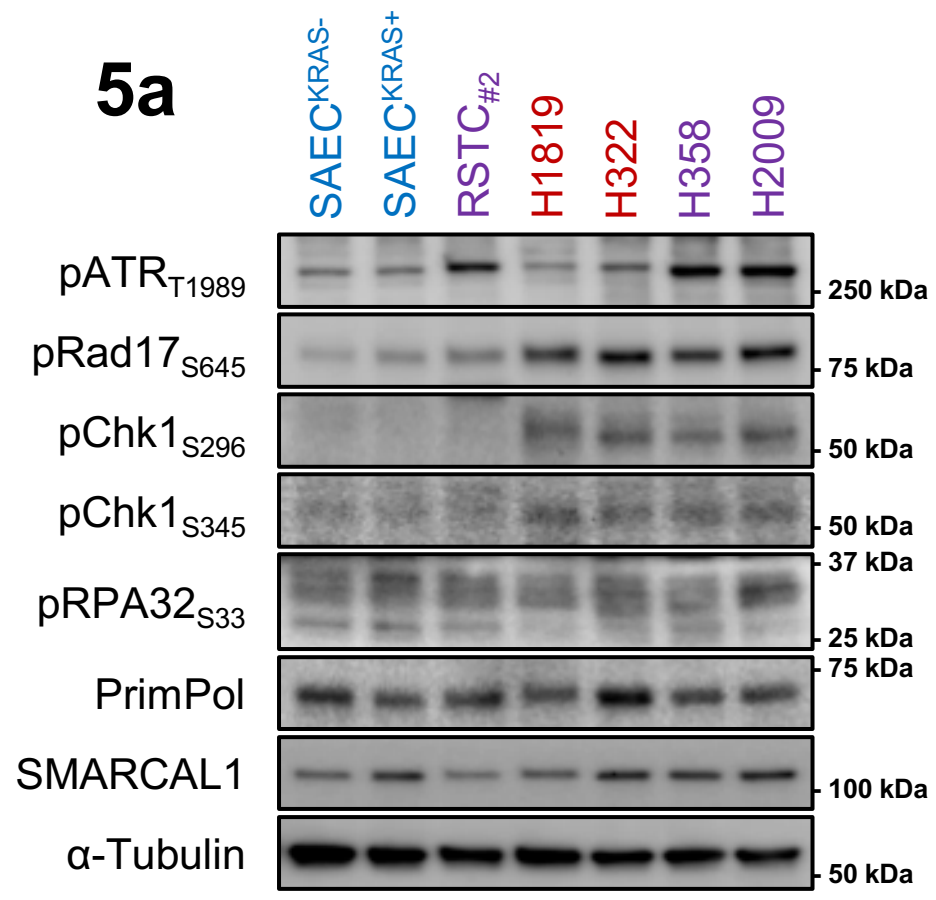

6. It is not clear how many times the authors repeated the DNA fiber assays. Most of the experiments show consistent reproducibility throughout different experiments. However, the statistical analysis should consider each independent experiment as replicate, not each fiber that has been measured.

Thank you for pointing this out. In the revised manuscript, we have clarified the number of independent DNA fiber experiments performed for each assay. For each sample, 100 or 150 DNA fibers were randomly selected and measured in each independent experiment, and the 300 pooled fiber measurements are shown as a

superplots. White dots indicate the mean value of independent biological replicates, and black bars indicates the mean of the pooled fiber measurements. Statistical differences were determined using ordinary one-way ANOVA followed by Tukey's multiple comparison test on mean value of pooled population. We have revised the figure legends and Methods accordingly. This revision does not change the conclusions of the DNA fiber analyses.

7. The analysis of ssDNA accumulation with the S1 DNA fiber assay in Figure 3A is missing the control of S1 digestion in control SAEC without mutant KRAS expression, to confirm that the cells do not present high levels of ssDNA.

Thank you for your suggestion. We have added this control to Revised Fig. 4b. As shown previously, S1 nuclease treatment does not shorten DNA fibers in SAECs without $KRAS^{G12V}$ expression, indicating that control SAECs do not contain high levels of S1-sensitive ssDNA gaps (PMID: 37591859). Consistently, in the revised Fig. 4b, SAECs showed no appreciable reduction in fiber length after S1 digestion. A similar lack of S1 sensitivity was observed in H1819 cells, a non-KRAS-driven lung cancer cell line. In contrast, H358 cells which are KRAS-driven and repriming-active, displayed marked S1 sensitivity, supporting ssDNA gap accumulation. Thus, the S1-dependent fiber shortening observed in H358 is not a general consequence of S1 treatment but reflects elevated ssDNA gap accumulation associated with PrimPol-dependent repriming.

4b

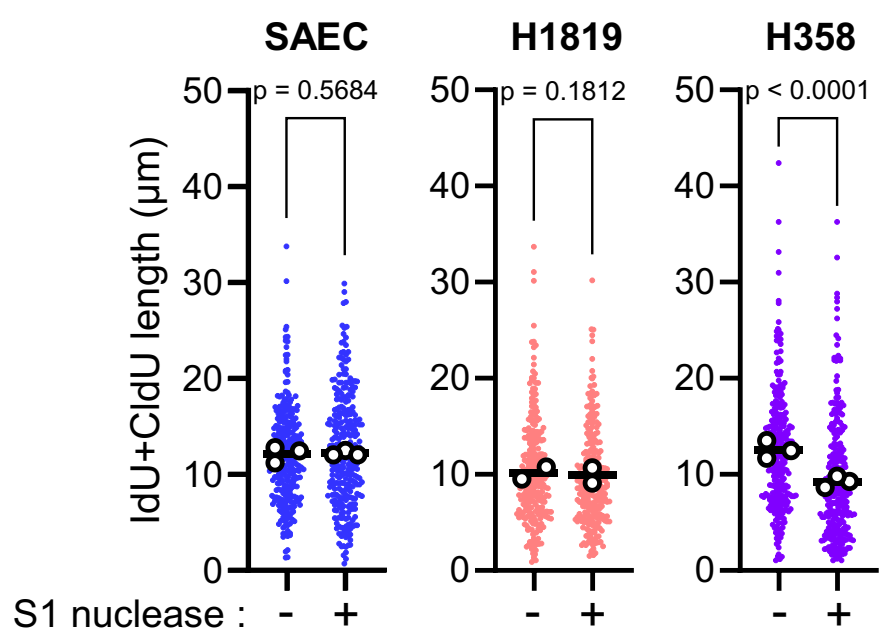

8. No cell cycle analysis is performed with the different treatments. In cases as the treatment with ATR and CHK1 inhibitors for 1 day, they can lead to a significant change in the number of cells in S phase that could influence the interpretation of the data.

We agree that ATR and Chk1 inhibitors can alter cell cycle distribution when used at high concentrations. However, in this study we used very low concentrations of ATRi and Chk1i (1 nM for 24 h), which we previously confirmed do not affect basal fork progression (PMID: 37591859). To directly address the reviewer's concern, we have now added FACS analyses showing that 1 nM ATRi or Chk1i does not cause a significant change in cell cycle distribution or the S-phase fraction (Revised Supplementary Fig. 4g). Thus, the DNA fiber phenotypes observed under these conditions are unlikely to result from changes in the number of S-phase cells.
Notably, despite the lack of detectable cell cycle effects, low-dose ATRi reduced PrimPol Ser255 phosphorylation-associated PLA signals (Revised Fig. 4k and Revised Supplementary Fig. 4n) and decreased fork progression specifically in KRAS-driven LUAD cells or RSTCs that rely on PrimPol-dependent repriming (Revised Fig. 4i, j). These findings support the interpretation that the observed effects reflect inhibition of the ATR-Chk1/PrimPol repriming pathway rather than indirect changes in cell cycle distribution.

S4g

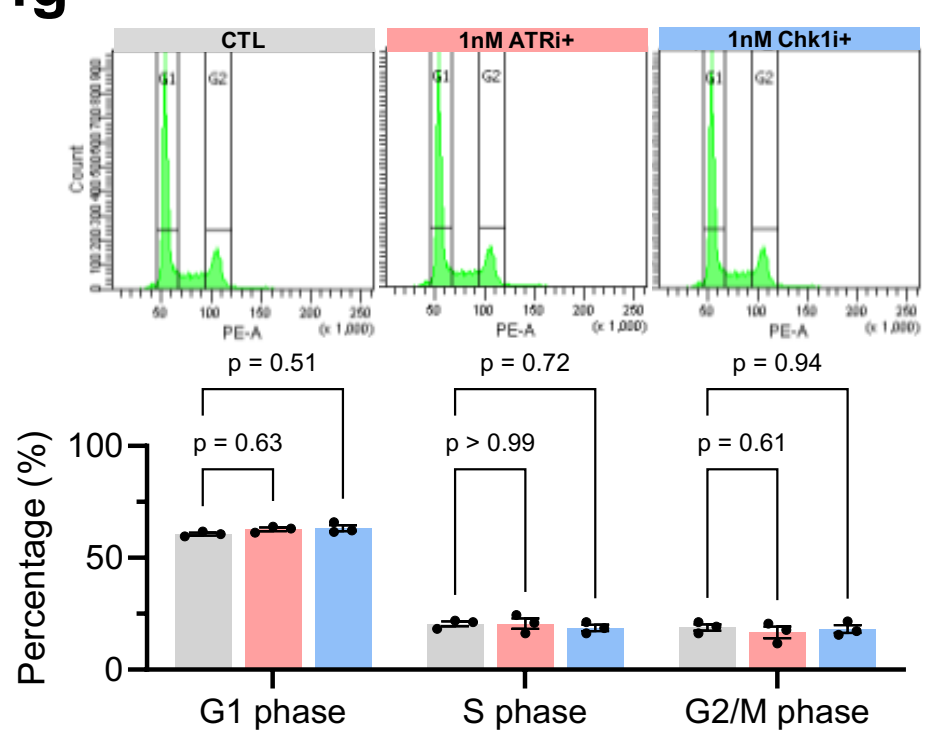

Reviewer #2 (Remarks to the Author):

I co-reviewed this manuscript with one of the reviewers who provided the listed reports. This is part of the Nature Communications initiative to facilitate training in peer review and to provide appropriate recognition for Early Career Researchers who co-review manuscripts.

We appreciate the reviewers' and co-reviewer's careful evaluation of our manuscript.

Reviewer #3 (Remarks to the Author):

In the study, the authors reported a novel function of PrimPol in regulating acute and persistent heterochromatin-associated replication stress induced by KRAS oncogene activation (K-HaRS). Mechanistically, phosphorylation of PrimPol by ATR signaling provides a molecular switch to coordinate its functions in slowing fork progression through SMARCAL1 regulation in the presence of acute K-HaRS, and in enabling repriming and replication progress across the heterochromatin barrier in the presence of persistent K-HaRS. Furthermore, loss of PrimPol renders a dependence on the error-prone Rev1-Pol ζ-dependent synthesis. These mechanistic findings reveal how PrimPol regulates replication stress tolerance (RST) in two different modes in different K-HaRS contexts (acute vs persistent) via ATR-mediated phosphorylation. In addition, the analysis of TCGA lung cancer datasets further highlights the clinical relevance of these two modes in tumor samples. Overall, this study utilized genetic, cell biology, and molecular biology approaches to dissect the mechanistic details of how PrimPol differentially coordinates cellular responses to K-HaRS at the acute stage and at the chronic stage. The experiments were well-designed and executed. Results from this study are expected to bring new understanding of how cells cope with replication stress, gain an optimal compromise of genome stability, and maintain survival fitness under oncogenic activation. There are some concerns regarding the experiment design and data interpretation.

We appreciate the constructive comments, which helped us improve the manuscript. As detailed below, we have performed additional experiments and revised the manuscript accordingly.

1. In Figure 1G, acute KRAS activation reduces cell proliferation. Does PrimPolS255A sufficiently restore cell proliferation in cells with acute KRAS-induced replication stress, or does it not? Conversely, does PrimPolS255A not sufficiently restore cell proliferation in cells with chronic KRAS-induced replication stress? It remains to be determined whether the function of PrimPolS255A mutant in regulating fork slowing but not repriming is sufficient to rescue the phenotype of cell proliferation in the presence of acute but not in the presence of chronic KRAS-activation.

Following this comment, we directly addressed this point by performing additional proliferation assays with PrimPol^{S255A}. In the time-to-proliferation assay (TTP assay shown in original Fig 1G), PrimPol^{S255A} expression in PrimPol knockout cells (ΔPP) did not restore the proliferation defect caused by acute KRAS activation (Reviewer Fig. 1a). We also noted that strong PrimPol^{S255A} expression itself reduced proliferation even in the absence of KRAS activation. Therefore, we further repeated the analysis using reduced dox concentrations that induced PrimPol^{S255A} expression close to the endogenous PrimPol level (0.08 μg/ml) (Reviewer Fig. 1b). Even under these lower-expression conditions, PrimPol^{S255A} did not restore cell proliferation under acute KRAS-induced replication stress. Thus, although PrimPol^{S255A} supports active fork slowing in the DNA fiber assay, this fork-level activity alone is not sufficient to rescue proliferation.
We also examined the chronic KRAS-driven cancer cell context using H358 cells. In contrast to acute KRAS-activated SAECs, PrimPol depletion itself had little effect on short-term proliferation, and PrimPol^{S255A} expression did not substantially alter proliferation (Reviewer Fig. 1c). Therefore, under these short-term assay conditions, we could not detect a proliferation phenotype that would allow us to evaluate rescue by PrimPol^{S255A} in H358 cell.
Overall, these results indicate that PrimPol^{S255A} can support fork-level active slowing, but cannot fully substitute for dynamically regulated PrimPol function during proliferation under acute KRAS-induced replication stress.

Rev. Fig. 1a

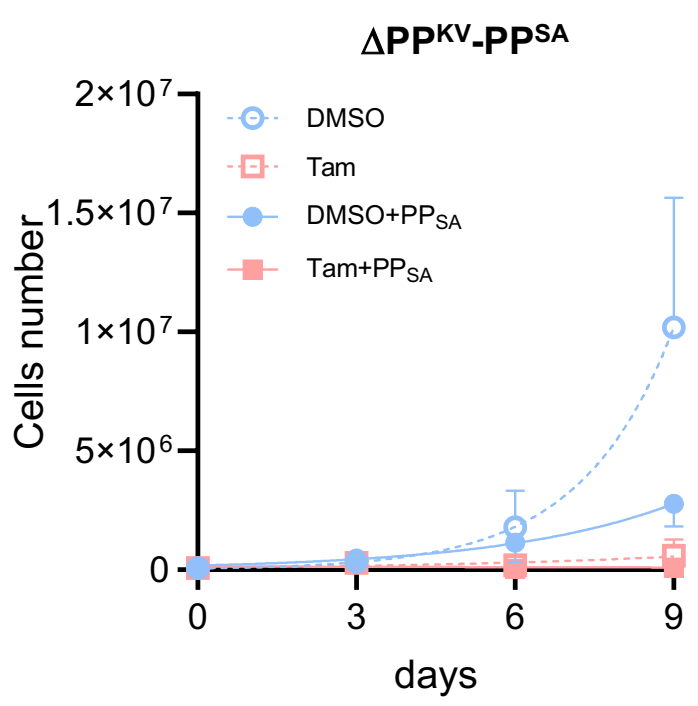

Rev. Fig. 1b

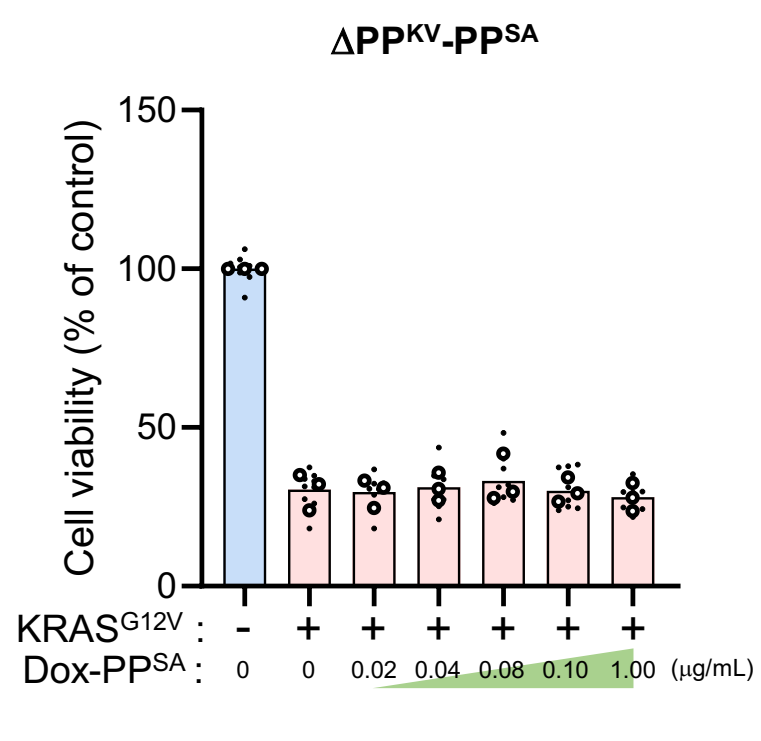

Rev. Fig. 1c

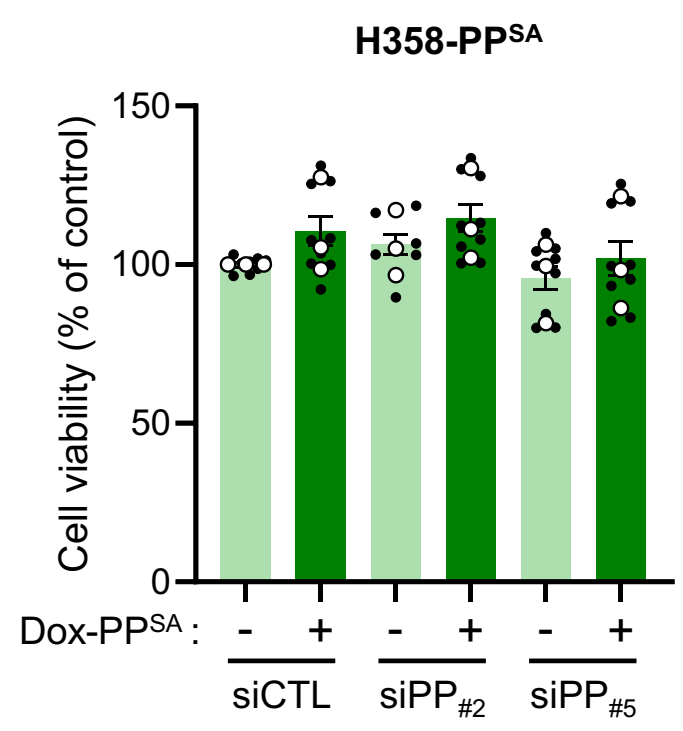

2. K-RAS induction can induce acute and persistent activation of DNA damage signaling pathways, such as ATR/ATM, as evidenced by previous studies. The project's premise is that acute K-RAS activation leads to basal ATR activity, while chronic K-RAS leads to high ATR activity. However, no experimental evidence is presented to demonstrate the extent of ATR activation at basal levels (phosphorylated or its downstream kinase substrates) in the cell models tested when acute K-RAS is present. It's intriguing that PrimPol S255 phosphorylation is restricted or prevented during the acute stage to allow its function in its unphosphorylated form if ATR signaling is present at low levels. Does the phosphorylated form of PrimPol abolish its binding to SMARCAL1?

Thank you for raising this important point. As described in our response to Reviewer #1-Comment 1, we have now added comparative immunoblot analyses of replication stress response (RSR) activation markers across $SAEC^{KRAS-}$ cells, acute $SAEC^{KRAS+}$ cells, replication stress-tolerant cells (RSTCs) derived from $SAEC^{KRAS+}$ cells (PMID: 37591859), and lung cancer cell lines (Revised Fig. 5a). These data show that acute KRAS activation induces detectable ATR/RSR signaling, particularly RAD17 Ser645 phosphorylation, indicating that the acute K-HaRS state is not devoid of ATR/RSR activity.
However, this global RSR activation did not simply mirror the PrimPol Ser255 phosphorylation-associated repriming state. To examine PrimPol phosphorylation, we generated a phospho-Ser255 PrimPol antibody, biochemically validated its specificity using Phos-tag and peptide kinase assays (Revised Supplementary Fig. 4l, m), and applied it to a PLA assay (Revised Fig.4k and Supplementary Fig. 4n, o). PrimPol Ser255 phosphorylation-associated PLA signals were preferentially detected in KRAS-driven H358 and H2009 cells and were reduced by ATR inhibition and PrimPol depletion (Revised Fig.4k and Supplementary Fig. 4n). These data indicate that PrimPol Ser255 phosphorylation is linked to ATR-Chk1 pathway activity but represents a more specific functional state associated with repriming.
We further tested whether the PrimPol Ser255 phosphorylation state affects SMARCAL1 binding. Co-immunoprecipitation analyses showed that the non-phosphorylatable $PrimPol^{S255A}$ mutant associated with SMARCAL1 more efficiently than $PrimPol^{WT}$ and $PrimPol^{S255D}$, whereas the phosphomimetic $PrimPol^{S255D}$ mutant retained detectable SMARCAL1 binding (Revised Fig. 2j). Thus, the phosphomimetic form did not completely abolish SMARCAL1 binding and may reduce the interaction compared with the S255A mutant. These results support a model in which the non-phosphorylated PrimPol state favors SMARCAL1 association during active fork slowing, whereas Ser255 phosphorylation is associated with the transition toward repriming.

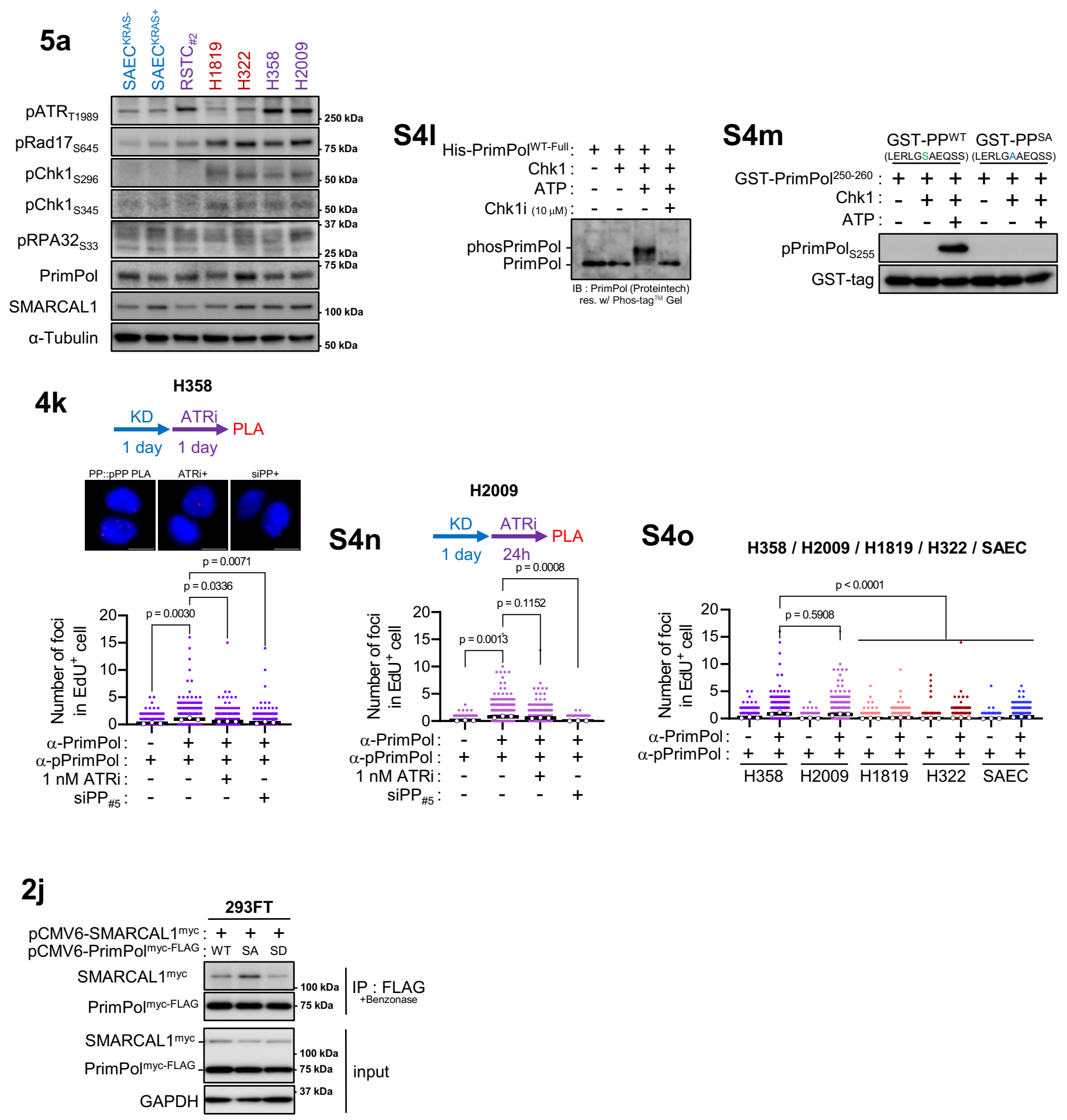

3. The study employed PrimPol phosphor-mutant as a genetic approach to elucidate the function of phosphorylation. Furthermore, in Fig. 4, a very low concentration of ATR inhibitor (1nM) was used to demonstrate the necessity of ATR in PrimPol-dependent repression in response to chronic K-HaRS. However, it remains uncertain whether this low level of ATR inhibitor treatment effectively abolished ATR activation and PrimPol phosphorylation. Additionally, considering the cross-activation of phosphorylation substrates by ATM, ATR, and DNAPK, it is unclear whether ATR inhibition alone is sufficient to eliminate PrimPol phosphorylation. Therefore, control experiments are essential to address these concerns.

We agree that appropriate controls are important to determine whether low-dose ATR inhibition affects the PrimPol phosphorylation-dependent pathway and whether ATM or DNAPK could compensate for ATR inhibition. In this study, we used 1 nM ATRi because this concentration does not substantially affect basal fork progression (PMID: 37591859) or cell cycle distribution (Revised Supplementary Fig. 4g), but suppresses PrimPol-dependent repriming in KRAS-driven LUAD cells and RSTCs that rely on PrimPol-dependent repriming (Revised Fig. 4i, j), as previously reported (PMID: 37591859).
To directly test whether this low-dose ATRi affects PrimPol Ser255 phosphorylation-associated signals, we performed PLA using our phospho-Ser255 PrimPol antibody. Treatment with 1 nM ATRi reduced PrimPol Ser255 phosphorylation-associated PLA signals in H358 and H2009 cells, indicating that this low-dose ATRi is sufficient to suppress the PrimPol Ser255 phosphorylation-associated PLA signals detected in our assay (Revised Fig. 4k, and Supplementary Fig. 4n).
We also examined the potential contribution of ATM and DNAPK. Cells were treated with 1 nM ATRi, 1 µM ATMi, or 1 µM DNAPKi, and pathway effects were monitored by immunoblotting (Revised Supplementary Fig 4i). Under these conditions, broad changes in ATR/RSR marker phosphorylation were not evident, indicating that these short-term inhibitor treatments did not globally collapse the replication stress response. In parallel DNA fiber assays, neither ATMi nor DNAPKi phenocopied the effect of ATRi on fork progression (Revised Supplementary Fig. 4j). These results indicate that ATM and DNAPK are unlikely to be the major kinases driving the PrimPol-dependent repriming phenotype under chronic K-HaRS conditions.
Thus, our conclusion does not rely on complete suppression of global ATR signaling. Rather, the key point is that low-dose ATR inhibition suppresses the PrimPol Ser255 phosphorylation-associated/repriming branch under conditions that do not broadly perturb replication or cell cycle progression.

4k

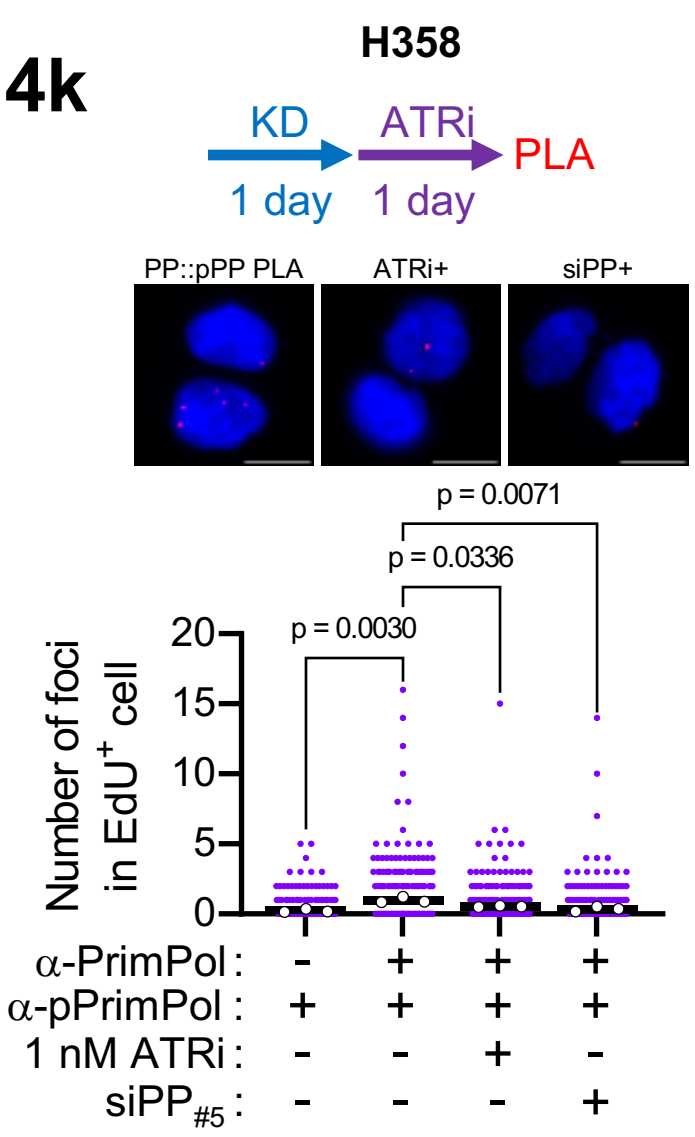

S4i

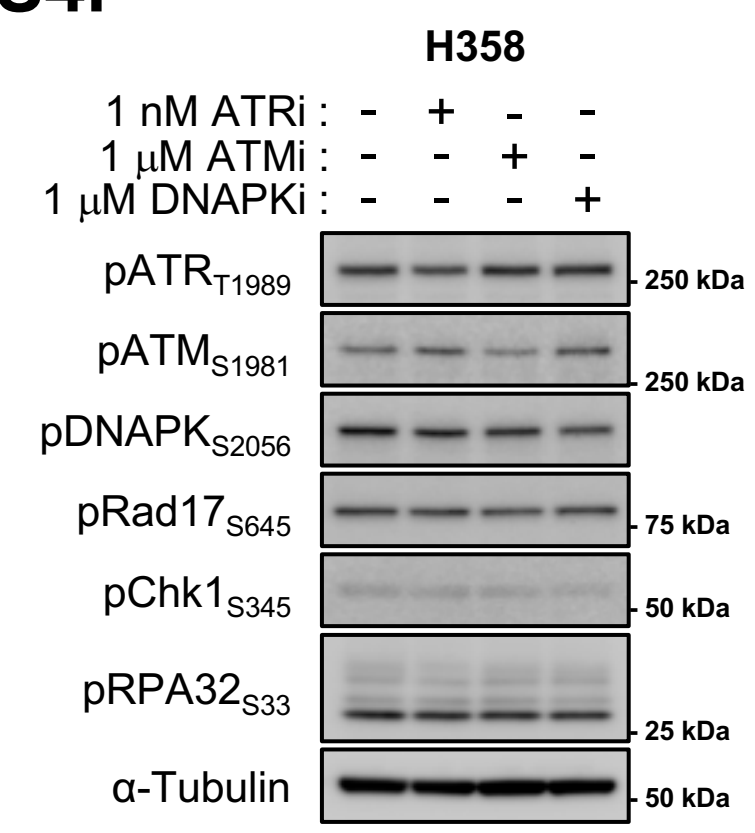

S4j

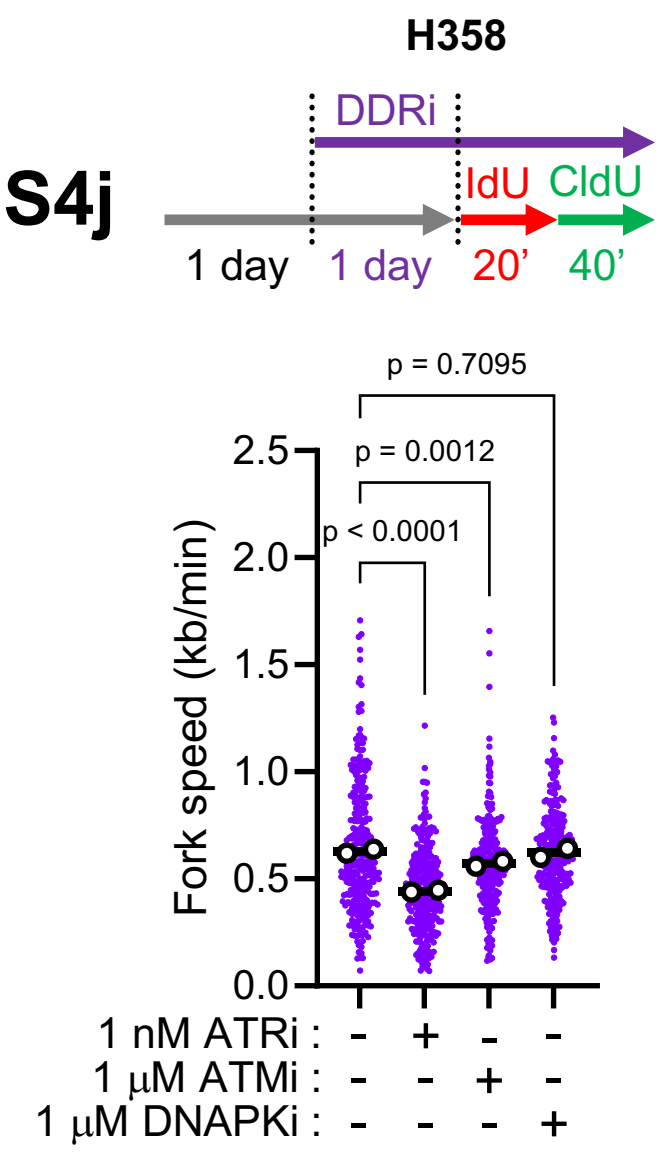

4. P53 deactivation frequently occurs in tumor cells as an escape mechanism from DNA damage checkpoint activation triggered by K-RAS activation. In animal models of various tumor types, loss of p53 is commonly used to facilitate tumor development in the presence of Kras mutations. If p53 is inactivated, does PrimPol play a role in slowing or repressing fork progression or cell proliferation in the presence of acute or chronic K-RAS? Does the loss of p53 override the requirement of PrimPol during the oncogenic transformation? Furthermore, how do these two distinct groups correlate with p53 mutation status, as per the TCGA analysis?

We agree that p53 status is an important consideration in KRAS-driven tumorigenesis. To address this point, we performed additional p53 depletion experiments in SAECs harboring the 4OHT-inducible $KRAS^{G12V}$ system. Efficient p53 depletion was confirmed by immunoblotting (Reviewer Fig. 2a). Under these conditions, p53

depletion did not alter the PrimPol-dependent acute KRAS-induced active fork slowing phenotype in the DNA fiber assay, indicating that the PrimPol-SMARCAL1-associated fork slowing response is not dependent on p53 in this model (Reviewer Fig. 2b).
We also examined the effect of p53 depletion on cell viability under acute KRAS activation. p53 depletion had only a modest effect and did not substantially restore cell viability under acute KRAS-induced replication stress (Reviewer Fig. 2c). These results indicate that p53 loss does not override the requirement for the replication stress response triggered by acute KRAS activation in SAECs.
Consistent with this conclusion, the KRAS-driven lung cancer cell lines used in this study represent p53-deficient contexts: H358 cells are TP53-null, and H2009 cells harbor the TP53 DNA-binding domain hotspot mutation R273L, according to Cellosaurus annotations. Nevertheless, these cells showed PrimPol Ser255 phosphorylation-associated PLA signals and PrimPol-associated repriming phenotypes (Revised Fig. 4b, k and Revised Supplementary Fig. 4a, n). Moreover, when PrimPol-dependent repriming was suppressed by low-dose ATR inhibition, p53-deficient KRAS-driven H358 cells showed an active fork-slowing phenotype (Revised Fig. 4i). These results indicate that the PrimPol-associated fork response is retained in p53-deficient KRAS-driven cancer cells.
Finally, to address whether the increased variant burden observed in the KRAS-mutant Polζ-prone group was confounded by TP53 mutation status, we repeated the TCGA analysis after excluding TP53-mutant tumors. The difference between the KRAS-mutant repriming-prone and Polζ-prone groups was retained after exclusion of TP53-mutant cases, indicating that the elevated variant burden in the Polζ-prone group is not simply explained by TP53 mutation status (Revised Supplementary Fig. 5d).
Together, these data support the conclusion that the PrimPol-associated replication fork response to KRAS-induced replication stress is largely independent of p53 status in our experimental system, and that the cohort-level genomic association is not driven by TP53-mutant tumors.

Rev. Fig. 2a

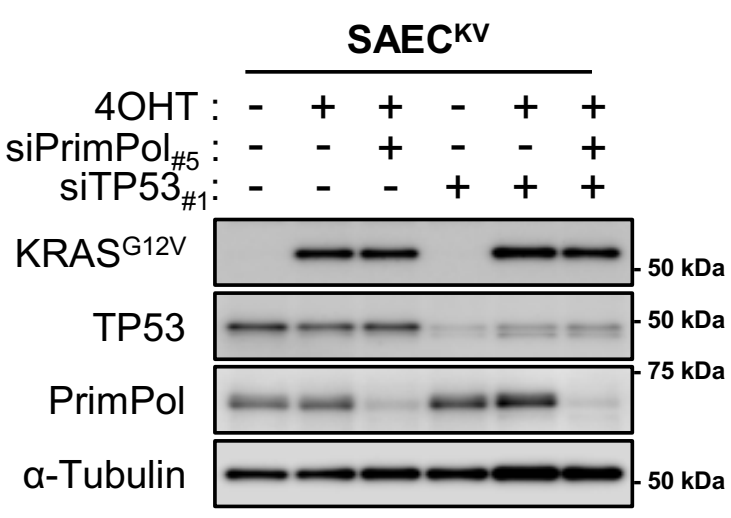

Rev. Fig. 2b

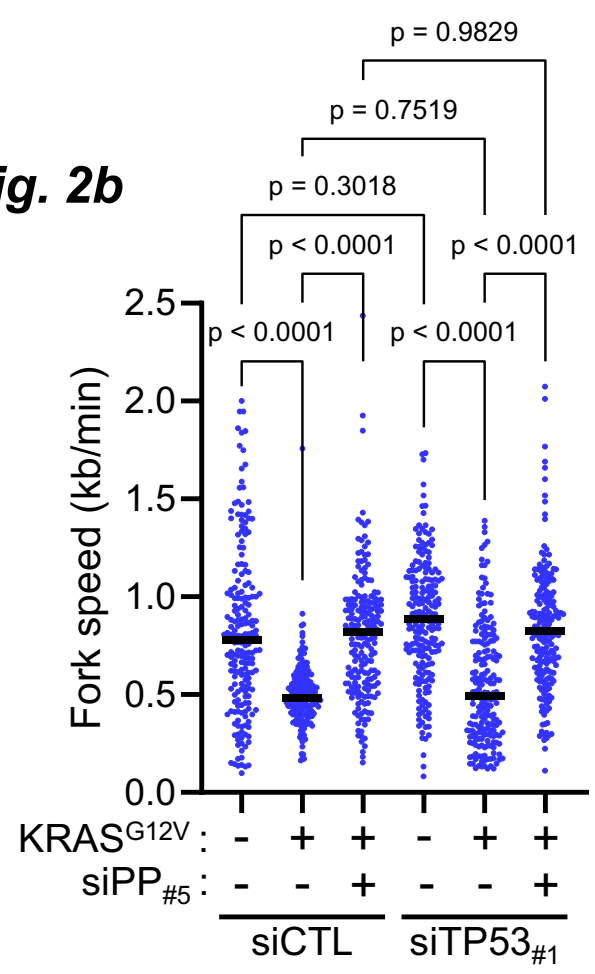

Rev. Fig. 2c

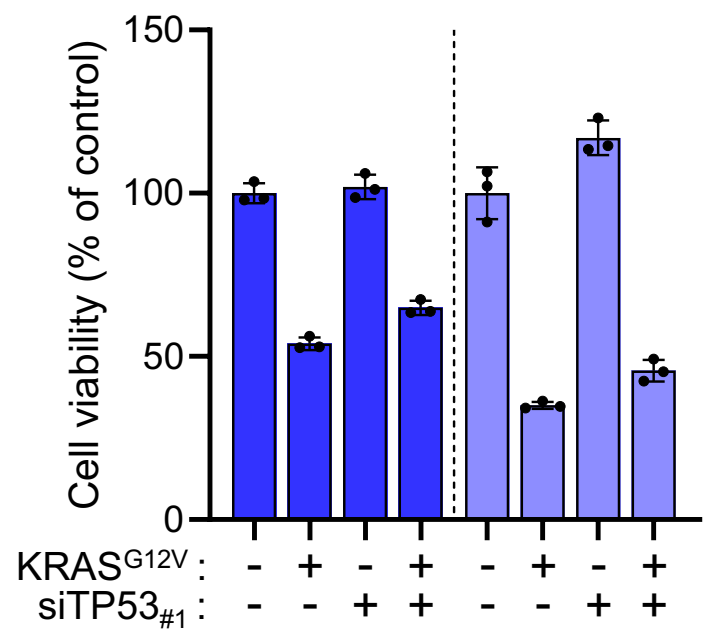

S5d

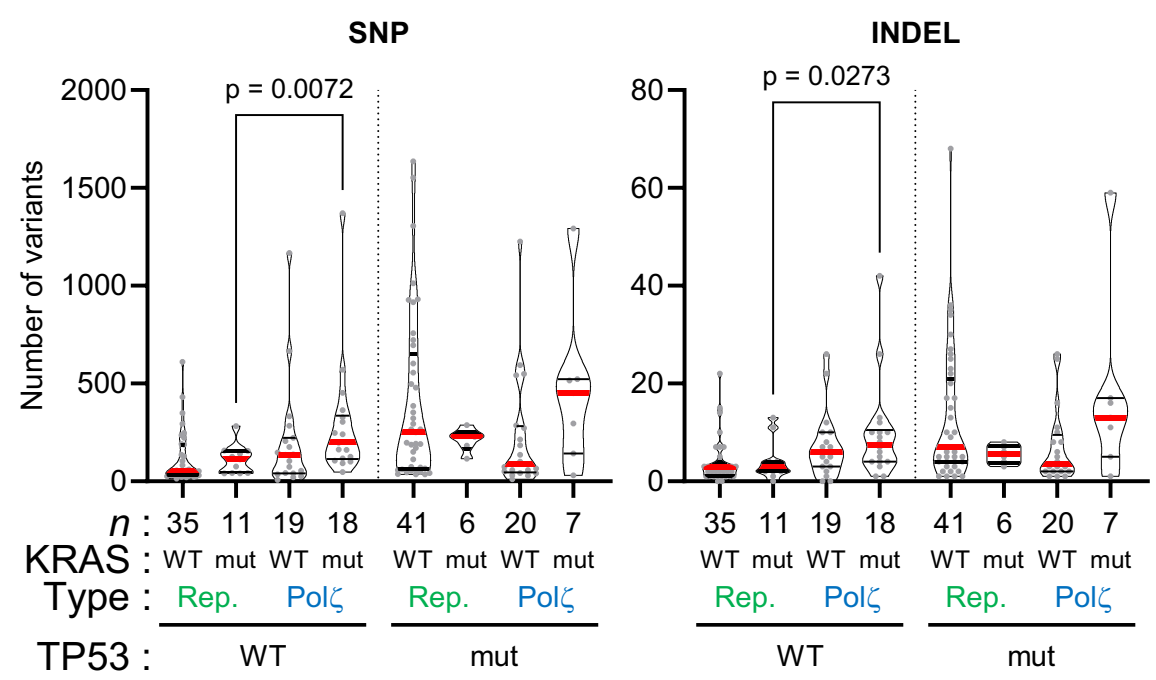

5. In addition, KRAS mutations are almost found in all pancreatic cancer tumors. Can the conclusions drawn from two modes be validated using pancreatic tumor TCGA samples, or are these observations highly specific to lung cancer models?

We agree that this is an important point. To assess whether the two-modes model may extend beyond LUAD, we performed exploratory analyses using TCGA PAAD and COAD samples, two tumor types in which KRAS mutations are frequent. In both cohorts, we observed trends consistent with the LUAD analysis, with the Polζ-prone group tending to show a higher variant burden than the repriming-prone group. However, PAAD has limited sample size and a very high prevalence of KRAS mutations, whereas COAD has substantial molecular heterogeneity, including differences in MSI/MMR status and other mutational processes. We therefore interpret these results as supportive but not definitive and have not included them in the revised manuscript. Instead, we focus the TCGA analysis on LUAD, which directly corresponds to the lung epithelial and lung cancer cell models used in this study.

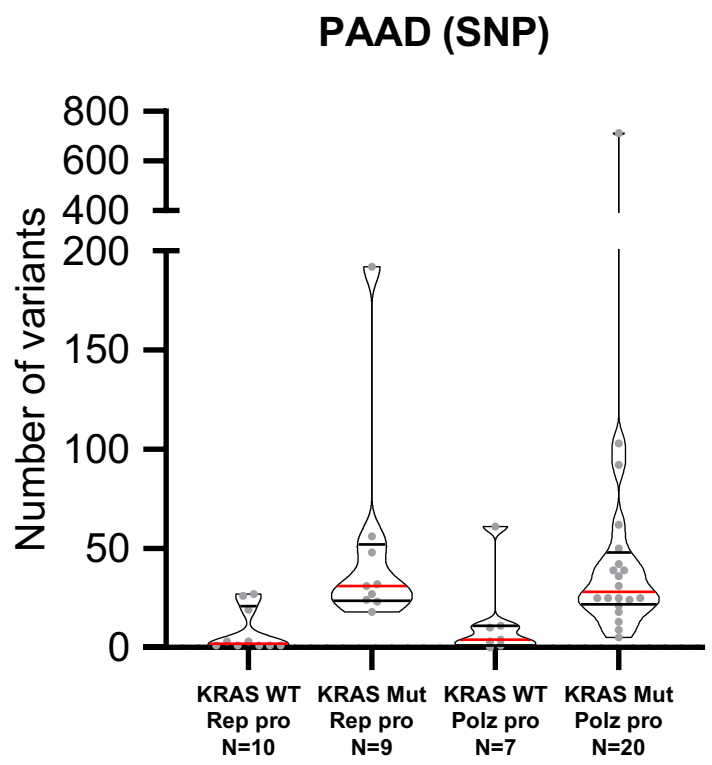

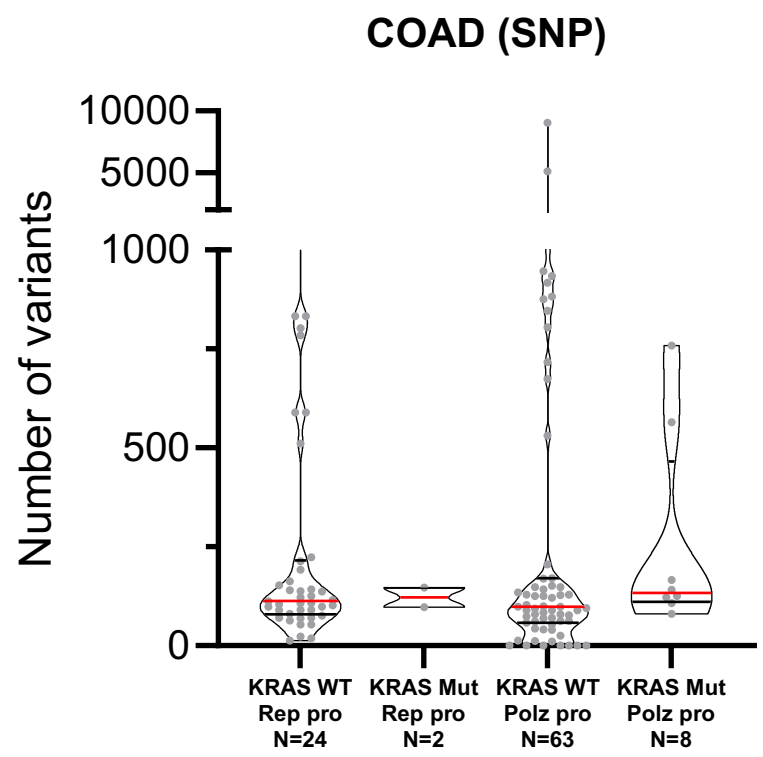

Reviewer #4 (Remarks to the Author):

This study has identified two distinct roles of PrimPol in replication in response to KRAS-induced replication stress. In an acute KRAS-induced heterochromatin-associated replication stress (K-HaRS), PrimPol functions with SMARCAL1 to slow down the replication fork progression suppressing Rev1 and polymerase zeta-dependent translesion DNA synthesis (TLS). In KRAS-driven tumor cells, however, ATR-dependent PrimPol repriming function is required to maintain replication fork speed. The authors therefore propose two distinct PrimPol-involved replication stress tolerance mechanism which is relying on the ATR activation mode in response to acute and chronic H-HaRS, respectively. While the role of PrimPol in acute KRAS-induced replication stress is clear and convincing, the mechanism in the chronic KRAS-induced response needs to be further strengthened. Overall, the study presents an interesting model for us to overview the dynamic regulation of replication stress response mechanism and genome instability during KRAS-induced tumor progression. However, there are two major concerns that need to be clarified.

We thank the reviewer for the positive and thoughtful evaluation of our study. We also appreciate the reviewer's constructive comments regarding the mechanism underlying the chronic K-HaRS response. As detailed below, we have performed additional experiments and revised the manuscript to address these concerns.

The chronic H-HaRS is not well defined. The authors appear to equal two KRAS-mutant cancer cells (mostly just H358 cells) to cells with chronic H-HaRS and argue that these cells possess high ATR activity which leads to PrimPol Ser 255 phosphorylation and suppression of SMARCAL1 to favor PrimPol-dependent repriming. Additional experiments and controls need to be included for the status of ATR, Chk1, PrimPol Ser255 phosphorylation, SMARCAL1, etc.

We appreciate the reviewer's suggestion. In the revised manuscript, we therefore incorporated RSTCs, which were derived from SAECs after prolonged $KRAS^{G12V}$ expression, as a SAEC-derived adapted/chronic K-HaRS model (PMID: 37591859). This addition allows us to connect acute KRAS-induced K-HaRS in SAECs with a SAEC-derived adapted/chronic state, while also comparing these states with established KRAS-driven lung cancer cells.
We now show that RSTCs retain K-HaRS-associated replication stress (Revised Fig.4f). When PrimPol was depleted in RSTCs, thereby impairing repriming, replication fork progression was compromised. This defect was rescued by DRB, GSK126, or HP1 depletion, indicating that the underlying replication obstacle in the adapted/chronic state remains transcription- and heterochromatin-associated (Revised Fig. 4g). Similar K-HaRS-associated fork phenotypes were also observed in KRAS-driven H358 and H2009 cells (Revised Fig. 4c, d, and Revised Supplementary Fig. 4b, c). Thus, adapted/chronic K-HaRS is operationally defined as a persistent oncogenic KRAS-associated replication stress state in which cells rely on PrimPol-dependent repriming to bypass transcription- and heterochromatin-associated replication obstacles.
We further clarified that the repriming-active state in adapted/chronic K-HaRS models is linked to ATR pathway activity and Chk1-dependent PrimPol Ser255 phosphorylation. Our previous study showed that, in ATR-high KRAS-induced repriming models that mimic the RSTC state, PrimPol Ser255 phosphorylation is required for PrimPol-dependent repriming and that low-dose ATR or Chk1 inhibition reduces fork progression in RSTCs (PMID: 37591859). In KRAS-driven H358 and H2009 cells, PrimPol Ser255 phosphorylation-associated PLA signals were detected and reduced by ATR inhibition and PrimPol depletion (Revised Fig. 4k and Revised Supplementary Fig. 4n). In H358 cells, low-dose ATR inhibition also exposed an active fork-slowing phenotype, consistent with suppression of the repriming branch and exposure of the underlying acute K-HaRS-like fork slowing response (Revised Fig. 5e).
Finally, as described in response to Reviewer #1, we added comparative immunoblot analyses of total PrimPol, SMARCAL1, and RSR activation markers across $SAEC^{KRAS-}$ cells, acute $SAEC^{KRAS+}$ cells, RSTCs, and lung cancer cell lines (Revised Fig. 5a). These analyses showed that total PrimPol and SMARCAL1 levels were broadly comparable, whereas RSR activation markers showed model-dependent changes. Importantly, these RSR markers did not simply mirror the PrimPol Ser255 phosphorylation-associated repriming state. Thus, global ATR/RSR activation is associated with, but does not fully define the PrimPol-dependent repriming program in the K-HaRS response.

4f

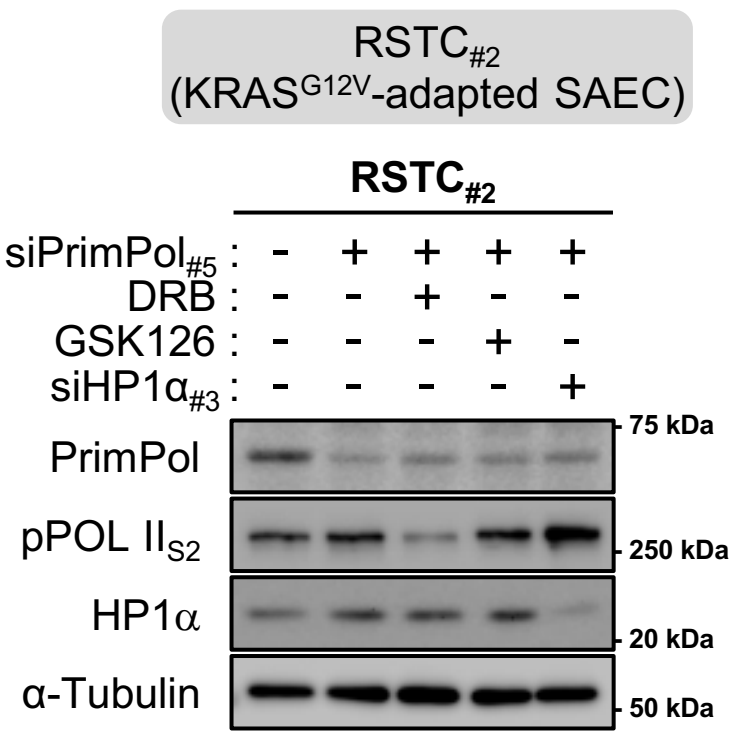

4g

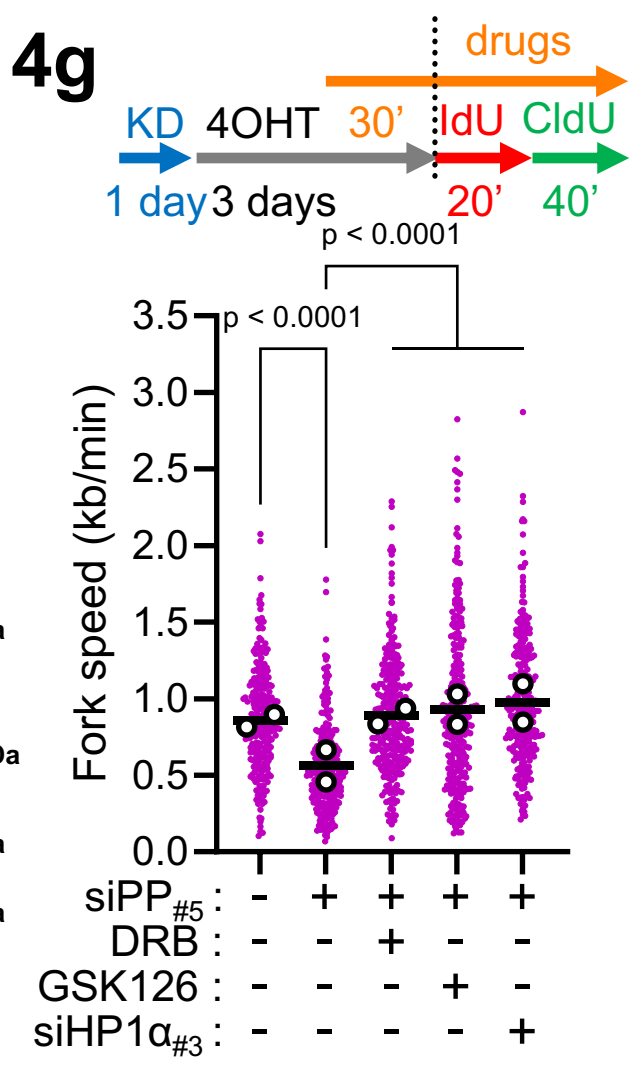

5a

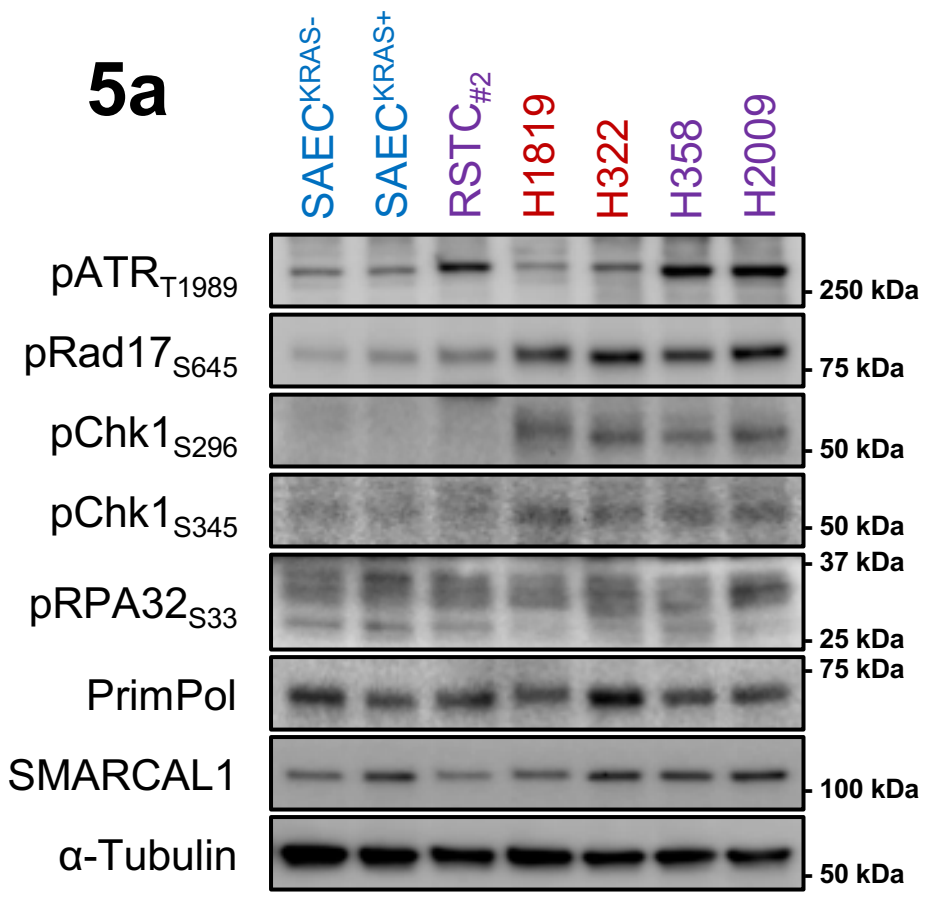

4k

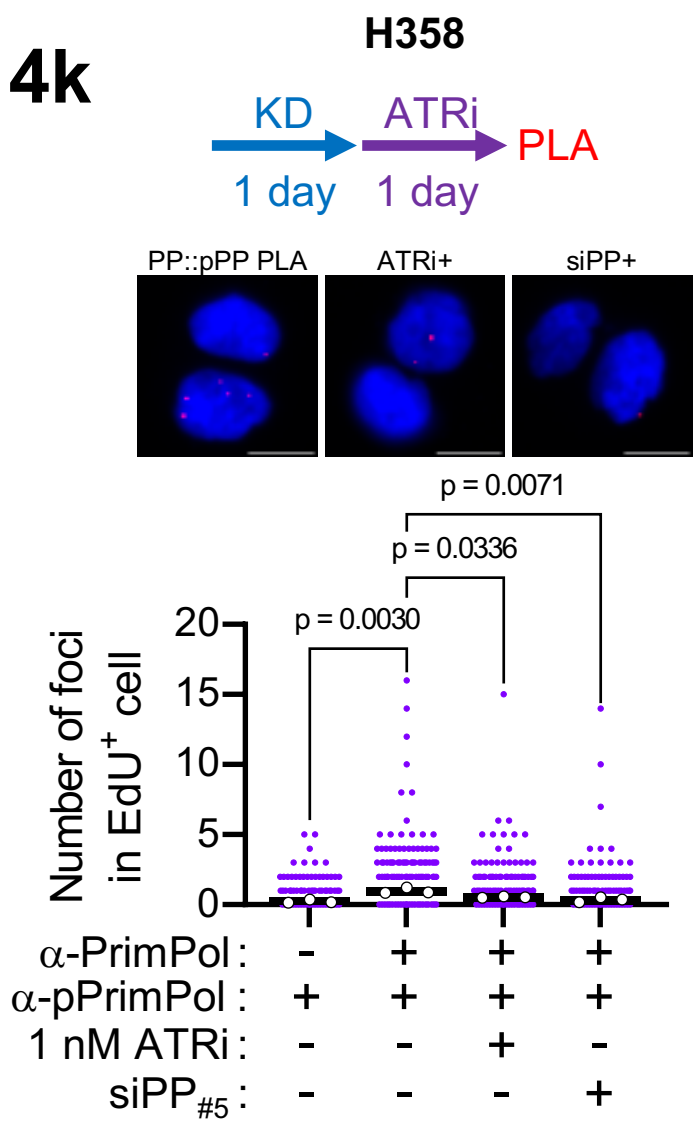

S4n

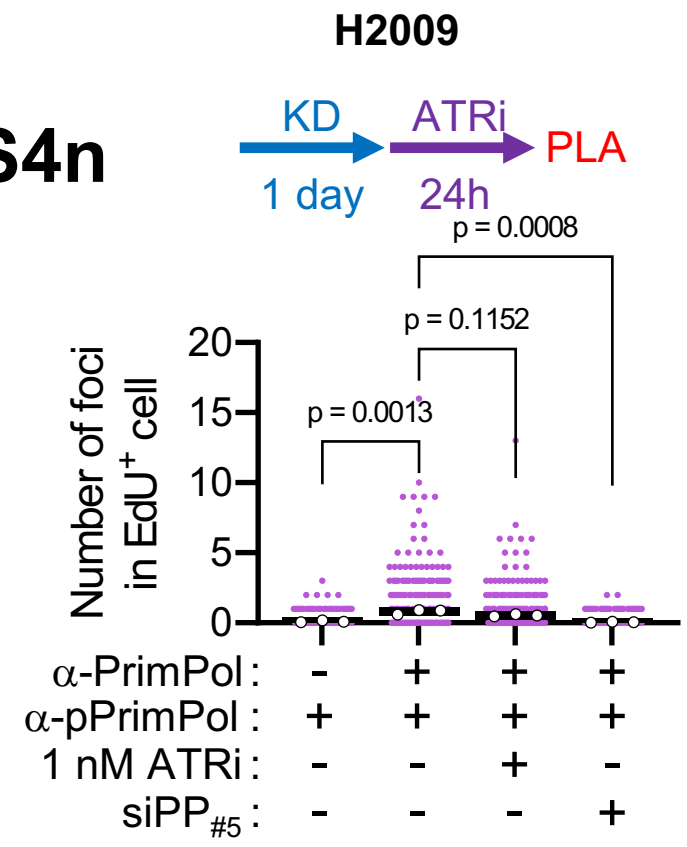

It is not clear whether only unphosphorylated PrimPol interacts with SMARCAL1. The authors show that S255A mutant of PrimPol interacts with SMARCAL1 and argue that this underlies the choice of fork slowing when ATR activity is low. However, it's not clear whether ATR activity regulates the interaction of PrimPol with SMARCAL1.

Fig. 2g, does PrimPol WT interact with SMARCAL1? Is the interaction subject to the regulation of ATR?

Thank you for raising this important point. To address this point, we added new co-immunoprecipitation analyses. In a 293FT expression system, the non-phosphorylatable PrimPol^{S255A} mutant associated with SMARCAL1 more efficiently than PrimPol^{WT} and PrimPol^{S255D} mutant (Revised Fig. 2j). Thus, SMARCAL1 binding is not restricted to the S255A mutant. WT PrimPol also interacts with SMARCAL1.
To further confirm this interaction at the endogenous level, we performed endogenous SMARCAL1 immunoprecipitation in SAECs and H358 cells. Endogenous PrimPol was co-immunoprecipitated with SMARCAL1 in SAECs undergoing acute K-HaRS (Revised Fig 2i), whereas this interaction was barely detectable in H358 cells under basal conditions (Revised Fig. 5b), suggesting that the PrimPol-SMARCAL1-associated fork-slowing configuration is available in normal epithelial cells but is suppressed or disengaged in repriming-dominant KRAS-driven cancer cells. Notably, ATR inhibition restored detectable PrimPol-SMARCAL1 association in H358 cells (Revised Fig. 5c). This finding is consistent with the functional re-engagement of SMARCAL1-dependent active fork slowing when ATR-dependent repriming is suppressed (Fig. 4i). Together with the enrichment of PrimPol Ser255 phosphorylation-associated PLA signals in repriming-active H358/H2009 cells (Revised Fig 4k and Revised Supplementary Fig. 4n), these data support a model in which the non-phosphorylated PrimPol state favors SMARCAL1 association, whereas Ser255 phosphorylation is associated with the transition toward the repriming-active state.

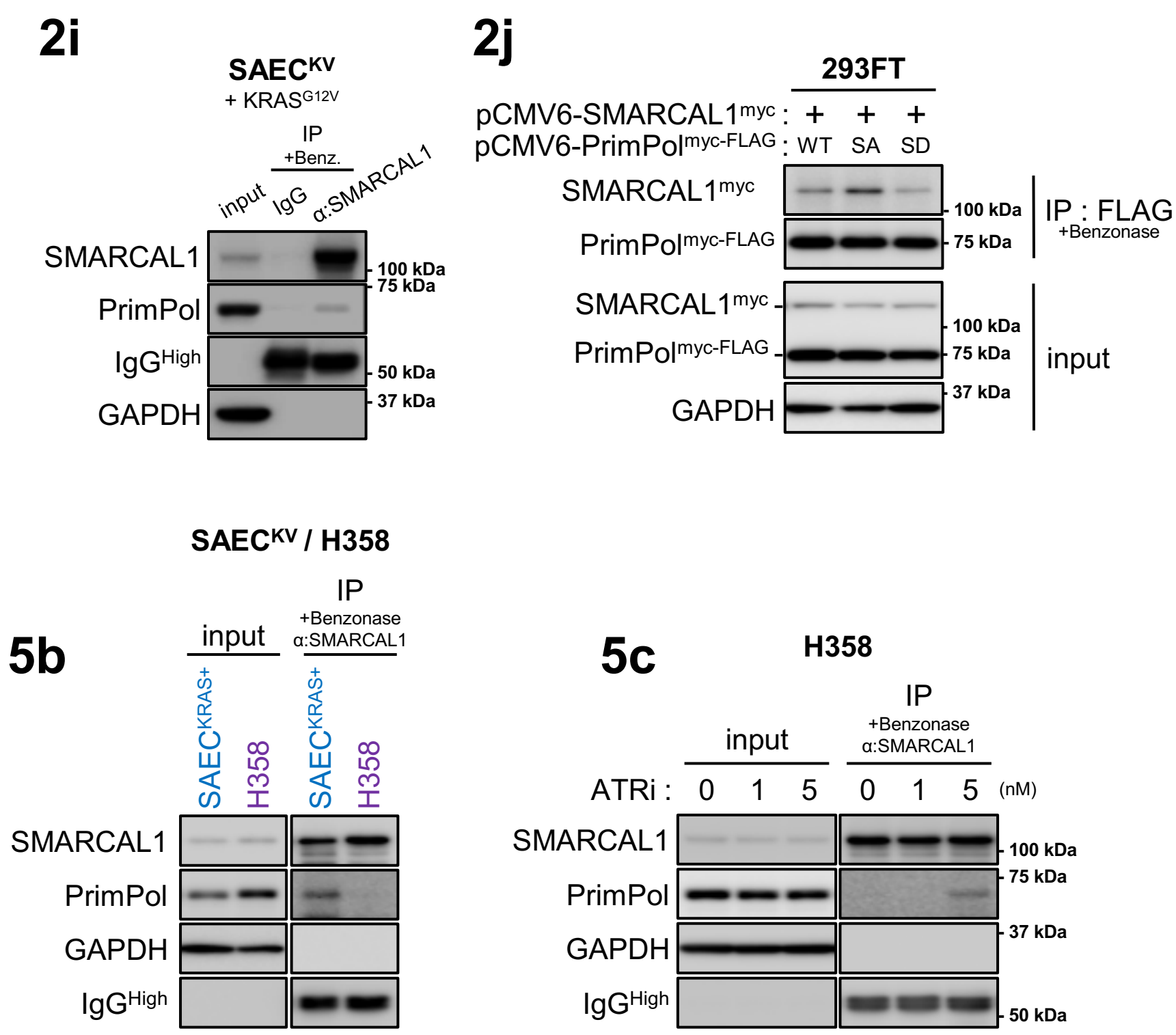

Fig. 2f, 5b, does PrimPol WT behave similarly as PrimPol S255A?

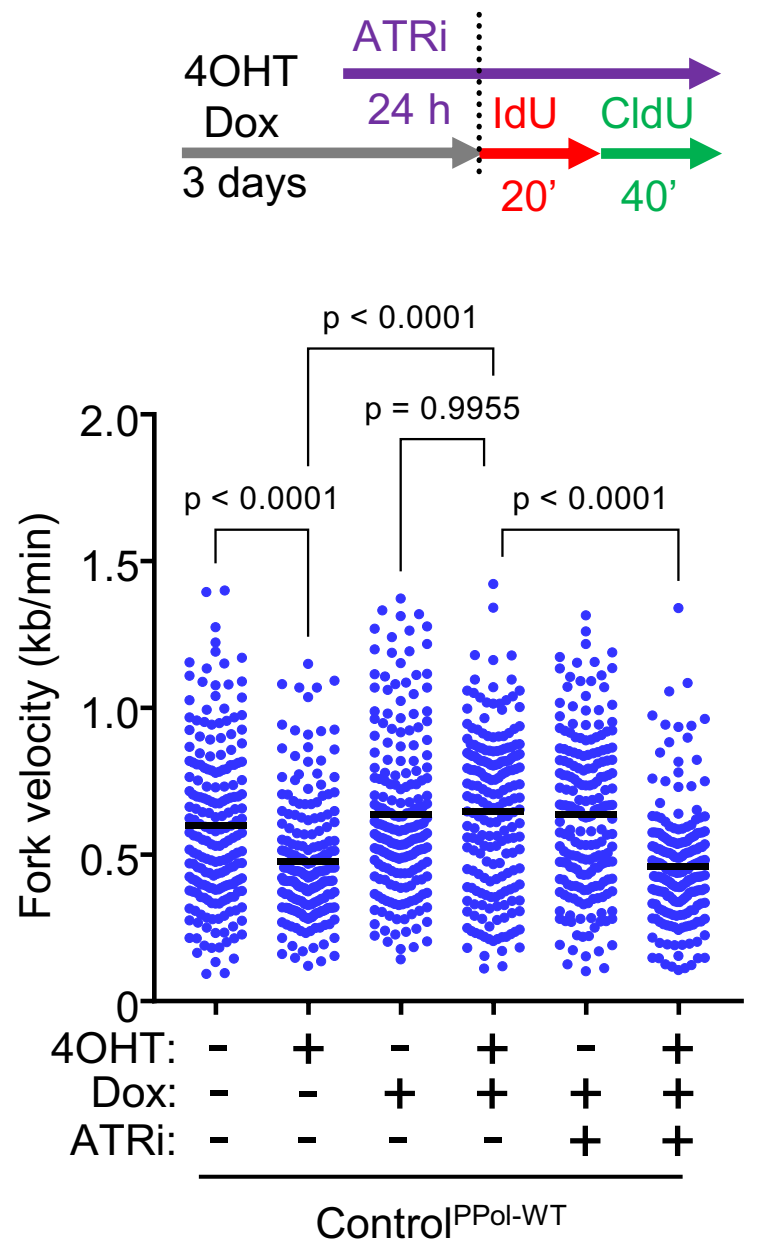

Fig. 3c from
Igarashi. et. al., ***Nat.Comm.*** (2023)
(PMID: 37591859)

PrimPol^{WT} and PrimPol^{S255A} should not be considered functionally identical under all conditions. PrimPol^{S255A} is a non-phosphorylatable mutant and was used to test the fork slowing-competent state of PrimPol. In Revised Fig. 2f, PrimPol^{S255A} rescued the active fork slowing defect caused by PrimPol depletion in acute K-HaRS. In Revised Fig. 5e, PrimPol^{S255A} did not induce active fork slowing in H358 cells under basal conditions, but supported active fork slowing when ATR-dependent repriming was suppressed by low-dose ATR inhibition. Thus, S255A reveals the fork slowing-competent function of PrimPol when the repriming branch is suppressed.
PrimPol^{WT}, in contrast, remains regulatable by ATR-Chk1 signaling. In our previous study, overexpression of PrimPol^{WT} in KRAS-induced SAECs promoted ATR-dependent fork progression with features consistent with repriming, rather than active fork slowing (Fig. 3c from PMID: 37591859). Thus, we would not expect PrimPol^{WT} and PrimPol^{S255A} to behave identically under all conditions. PrimPol^{WT} may support the fork-slowing mode when maintained in a non-phosphorylated or low-ATR context, but it can also shift toward the repriming mode depending on expression level and ATR pathway activity.

Fig.5 and S5, it lacks comparison between Kras wt and Kras mt tumor for the status of ATR, PrimPol and SMARCAL1?

We thank the reviewer for pointing this out. We added a comparison of genetic alterations in ATR, PRIMPOL, SMARCAL1, and KRAS in TCGA-LUAD tumors. This analysis showed that alterations in ATR, PRIMPOL, and SMARCAL1 are rare, with virtually no truncating alterations, and show no significant co-occurrence or mutual exclusivity with KRAS alterations. Thus, the status of these genes could not be meaningfully compared between

$KRAS^{WT}$ and $KRAS^{mut}$ tumors based on mutation status alone. Therefore, we used mRNA expression levels, rather than genetic alteration status, to define the repriming-prone and Polζ-prone groups.

S5a

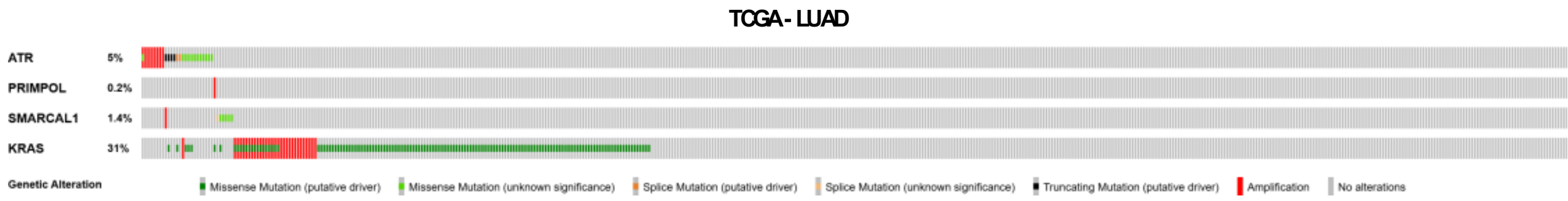

A	B	Neither	A Not B	B Not A	Both	Log2 Odds Ratio	p-Value	Tendency
PRIMPOL	KRAS	344	0	152	1	>3	0.308	N/A
ATR	KRAS	325	19	147	6	-0.518	0.513	N/A
SMARCAL1	KRAS	338	6	152	1	-1.432	0.682	N/A
